# Comparative Analyses of Transcriptional Profiles in Mouse Organs Using a Pneumonic Plague Model after Infection with Wild-Type *Yersinia pestis* CO92 and Its Braun Lipoprotein Mutant

**DOI:** 10.1155/2009/914762

**Published:** 2010-01-20

**Authors:** Cristi L. Galindo, Scott T. Moen, Elena V. Kozlova, Jian Sha, Harold R. Garner, Stacy L. Agar, Ashok K. Chopra

**Affiliations:** ^1^Department of Microbiology and Immunology, The University of Texas Medical Branch, Galveston, TX 77555-1070, USA; ^2^Virginia Bioinformatics Institute, Virginia Polytechnic and State University, Blacksburg, VA 24061, USA

## Abstract

We employed Murine GeneChips to delineate the global transcriptional profiles of the livers, lungs, and spleens in a mouse pneumonic plague infection model with wild-type (WT) *Y. pestis* CO92 and its Braun lipoprotein (Δ*l* *p* *p*) mutant with reduced virulence. These organs showed differential transcriptional responses to infection with WT *Y. pestis*, but the overall host functional processes affected were similar across all three tissues. Gene expression alterations were found in inflammation, cytokine signaling, and apoptotic cell death-associated genes. Comparison of WT and Δ*l* *p* *p* mutant-infected mice indicated significant overlap in lipopolysaccharide- (LPS-) associated gene expression, but the absence of Lpp perturbed host cell signaling at critical regulatory junctions resulting in altered immune response and possibly host cell apoptosis. We generated a putative signaling pathway including major inflammatory components that could account for the synergistic action of LPS and Lpp and provided the mechanistic basis of attenuation caused by deletion of the *lpp* gene from *Y. pestis* in a mouse model of pneumonic plague.

## 1. Introduction

The gram-negative bacterium *Yersinia pestis* is the etiological agent of plague. *Y. pestis* is transmitted to humans through the bite of an infected flea or inhalation of the organisms, resulting in bubonic, pneumonic, or septicemic forms of plague [1]. *Y. pestis* has attracted much interest recently because of its potential as a weapon of bioterrorism. Following entry within a host, *Y. pestis* evades the host immune system and replicates in the lymph nodes, ultimately leading to lymph node necrosis and death if untreated [2–4]. Histological evidence indicates that bacteria within neutrophils are killed, while bacteria within macrophages and dendritic cells survive and go on to express various virulence determinants, which allow bacterial growth and their eventual release from the macrophages [5–7]. For example, F1 (capsular) antigen [8] and type III secretion system (T3SS) effectors [9] are expressed only at 37°C and have been shown to modulate the host response so that *Y. pestis* becomes resistant to subsequent phagocytosis. The use of these antiphagocytic mechanisms has led researchers to suggest that *Y. pestis* is predominantly an extracellular pathogen in the mammalian host [9, 10]. However, a strong cell-mediated immune response to *Y. pestis* infection is seen in immunized mice, suggesting that immune cells are also needed to clear either intracellular bacteria or extracellular *Y. pestis* that have been opsonized. A T-cell component of protection against *Y. pestis*, in the absence of antibody, has been established [11, 12]. In unvaccinated individuals, low doses of *Y. pestis* can be resolved following combined treatment with the T helper1- (Th1-) associated cytokines interferon (IFN)-*γ* and tumor necrosis factor (TNF)-*α* [13]. These studies suggest that cell-mediated immune responses are important for protection against *Y. pestis*.

The ability of *Yersinia* species to infect and replicate within a host is primarily due to the bacterial expression and implementation of the T3SS [14]. T3SS is comprised of a molecular syringe-like complex that injects effector molecules into the target host cell enabling the bacteria to inhibit innate and acquired immune functions as well as to induce apoptosis. There are specific *Yersinia* outer membrane proteins (Yops) that have been studied extensively and characterized as inhibitors of specific biological processes that promote the survival of *Yersinia* species within the host. Specifically, the proteins YopE, -H, -J, -M, -O, -P, and -T disrupt cytoskeletal dynamics, inhibit innate and acquired immune functions, and promote apoptosis [15, 16].

The outer membrane of gram-negative bacteria is comprised of many different proteins that help maintain the structural integrity of the bacterial cell envelope. One particularly abundant lipoprotein, designated murein (or Braun) lipoprotein (Lpp), is associated with the outer membranes of bacteria within the family *Enterobacteriaceae* [17]. Earlier studies indicated that Lpp (6.3 kDa) from enteropathogenic bacteria not only synergized with lipopolysaccharide (LPS) to induce septic shock but also evoked the production of TNF-*α* and interleukin 6 (IL-6) in both LPS-responsive and LPS-nonresponsive mice and in mouse peritoneal exudate macrophages, suggesting an alternative signaling mechanism for Lpp [18]. In fact, a subsequent study showed that Lpp signals through Toll-like receptor-2 (TLR-2) and not TLR-4, which LPS utilizes for cell signaling [19]. Our more recent data provided evidence that Δ*lpp* mutants of *Y. pseudotuberculosis* and *Y. pestis* KIM/D27 were attenuated in mice, an effect that could be complemented [20]. In the latter strain of *Y. pestis* KIM/D27, a 102-kb pigmentation locus (*pgm*) was deleted, resulting in the attenuation of the WT bacteria [20]. Importantly, immunization of mice with this mutant provided protection to animals against pneumonic plague invoked by intranasal inoculation of *Y. pestis* CO92 [20].

Most bacterial virulence genes are regulated upon entering the host. Global regulators have the ability to modulate multiple operons that belong to different metabolic pathways and are important for bacteria to adapt to new conditions. Recently, several laboratories have established an intranasal mouse model of the pneumonic plague infection and have investigated the host-pathogen interaction by pathological survey and bacterial gene expression microarrays [3]. Liu et al. [21] examined the transcriptional profile of mice infected with *Y. pestis* strain 201, which is avirulent in humans, and reported upregulation of host cytokines that might mimic what would be observed during human infection [21]. In our study, we investigated the transcriptional profiles of mice challenged by the intranasal route with *Y. pestis* CO92, a clinical isolate that is virulent in both mice and humans that would presumably better model human disease. We also examined the transcriptional profile of a *Y. pestis* CO92 Δ*lpp* mutant and compared the results to mice infected with WT bacteria and found that *Y. pestis* CO92 Δ*lpp* mutant infection of the lung caused upregulation of many genes encoding major proteins of the host immune system. Interestingly, we found a number of unique genes which were expressed differently in all three tissues of mice infected with the Δ*lpp* mutant but were not altered by WT *Y. pestis* CO92 infection. This study provided new information on the dynamic of the liver, lung, and spleen host transcriptional responses to infection with WT *Y. pestis* CO92 and its Δ*lpp* mutant.

## 2. Materials and Methods

### 2.1. Bacterial Strains

WT *Y. pestis* CO92 was obtained from the Centers for Disease Control and Prevention (CDC, Atlanta, GA) and maintained in our restricted access biosafety level- (BSL-) 2 laboratory. The construction and characterization of the strain deficient in the expression of the *lpp* gene were previously described in detail [20]. All bacteria were grown in Brain Heart Infusion broth (BHI, Difco, Voigt Global Distribution Inc, Lawrence, KS) at 28°C prior to infection of mice.

### 2.2. Animal Studies

Swiss-Webster female mice (Charles River Laboratories, Wilmington, MA) 5-6 weeks of age were infected intranasally with 5 LD_50_ of either WT or Δ*lpp* mutant of *Y. pestis* CO92 [20]. Uninfected mice were used as controls. At either 12 or 48 hours post infection (p.i.), 3 mice per group were euthanized and the lungs, livers, and spleens were harvested and homogenized in 1 mL of RNALater (Ambion/Applied Biosystems, Austin, TX) using 50 mL tissue homogenizers (Kendell, Mansfield, MA). RNA was isolated from the tissue homogenates and purified using RNAqueous (Ambion). After an overnight precipitation, the RNA was resuspended in 20 *μ*L of diethylpyrocarbonate- (DEPC-) treated water and hybridized to Affymetrix GeneChip Mouse Genome 430 2.0 arrays, performed by the Molecular Genomics Core at UTMB Galveston, Texas, per manufacture protocols. The arrays had 45,000 probe sets representing more than 39,000 transcripts derived from ∼34,000 well-substantiated mouse genes. The experiments were performed in triplicate (biological replicates), generating a total of 45 arrays.

### 2.3. Normalization and Initial Characterization of Arrays

Data were Robust Multichip Average (RMA) normalized and log transformed using GeneSifter software (VizX Labs, Seattle, WA). Based on regression analysis of experimental replicates, there was an acceptable level of variation between each array (R^2^ = 0.96 ± 0.03). Raw and processed data (a total of 45 arrays) were deposited in the Gene Expression Omnibus (GEO) online (http://www.ncbi.nlm.nih.gov/geo) database (Accession GSE18293).

### 2.4. Analysis Methods

After normalization, data were considered separately for the three tissue types of mice. Data were further separated, based on time post infection and gene expression alterations that occurred in response to the WT *Y. pestis* CO92 and its Δ*lpp* mutant. This resulted in four analyses per tissue type (uninfected versus WT-infected animals and WT-infected versus mutant-infected animals at 12 hours and 48 hours p.i.). ANOVA was performed for each comparison, and only genes with *P* values of ≤.05 were considered for further analyses. Subsequent filtering was performed dependent upon group comparison types, as detailed below. Hierarchical clustering was employed on normalized and log transformed signals using GenSpring GX 10.0 (Agilent Technologies, Santa Clara, CA).

### 2.5. Data Analysis of Uninfected Controls versus WT-Infected Animals

For each time point, normalized signal values were averaged and pairwise comparisons were performed using GeneSifter. Only alterations (control versus WT-infected) of at least 2.0-fold were considered for further studies. Student's *t*-test with Benjamini and Hoshberg correction was also performed using GeneSifter. However, only the *P* value without correction was used to filter data (*P* ≤ .05), because natural biological variation was greater for some tissues than for others. All possible individual pairwise comparisons were performed using Spotfire DecisionSite 9.0 software (Spotfire, Inc., Sommerville, MA). An alteration of at least 1.5-fold was expected for each of the 9 possible comparisons between controls versus WT-infected samples (for each time point). Any alteration observed between uninfected and WT-infected animals was expected to be at least 50% greater than the fold change calculated for each uninfected control (C1 versus C2, C2 versus C3, and C1 versus C3).

### 2.6. Data Analysis of WT versus Δlpp Mutant-Infected Animals

For each time point, normalized signal values were averaged and pairwise comparisons were performed using GeneSifter. Only alterations (WT- versus Δ*lpp*-infected) of at least 1.5-fold were considered for further analysis. Student's *t* test was performed using GeneSifter, with the expectation of a *P* ≤ .05. All possible individual pairwise comparisons were performed using Spotfire DecisionSite 9.0 software (Spotfire, Inc.). An alteration of at least 1.5-fold was expected for each of the 9 possible comparisons between WT-infected and Δ*lpp*-infected samples for each time point. Any alterations observed between WT-infected and Δ*lpp* mutant-challenged animals were expected to be at least 50% greater than the fold change calculated for each uninfected control (C1 versus C2, C2 versus C3, and C1 versus C3). An alteration of at least 2.0-fold (on average) was expected between either uninfected versus WT-infected or uninfected versus Δ*lpp* mutant-infected samples. This step was intended to eliminate any presumably spurious alterations observed between WT-infected and Δ*lpp* mutant-challenged animals that was not normally affected by infection or altered in response to the Δ*lpp* mutant as compared to healthy animals.

## 3. Results

### 3.1. General Gene Expression Changes in All Tissues from WT *Y. pestis* CO92- and Its Δlpp Mutant-Infected Mice

The host transcriptional responses to infection with WT *Y. pestis* and its Δ*lpp* mutant in an inhalational mouse model of pneumonic plague were studied. Mice were infected for 12 or 48 hours, and RNA was isolated from livers, lungs, and spleens for processing and hybridization to Affymetrix GeneChip Mouse Genome 430 2.0 arrays. Uninfected animals served as controls, and experiments were performed in triplicate, which generated a total of 45 arrays (see summary in Supplementary Table 1 available online at doi:10.1155/2009/914762). A stringent data analysis method was employed, including analysis of replicate samples and subsequent elimination of naturally variable transcripts, to increase the reliability of the results and to greatly minimize false positives. Each experimental group was separately compared to appropriate uninfected control tissues, and the overall results of the analysis are shown in Table 1. In our initial GeneChip experiments, we also used times points of 24, 36, and 60 hours (data not shown); however, optimal transcriptional profiling changes were observed at 12 and 48 hours p.i.. Consequently, we focused only on these two time points.

Three general trends were apparent regarding the overall response of the host to *Y. pestis* infection: (1) host transcriptional responses increased dramatically between 12 hours and 48 hours p.i., (2) the liver transcriptome was more profoundly perturbed, compared to spleen or lung tissues, in WT *Y. pestis*-infected mice, and (3) there was a drastic difference in host transcriptional responses of mice infected with the Δ*lpp* mutant, dependent on time course and tissue. For example, many genes were differentially expressed in Δ*lpp* mutant-infected lungs (109 transcripts) and livers (256 transcripts) at 48 hours p.i., compared to WT *Y. pestis*-infected animals, whereas only modest differences were observed at the earlier time point (12 hours p.i.) in the spleens (25 transcripts) and livers (11 transcripts) of mice infected with the Δ*lpp* mutant compared to WT bacteria (Table 1).

Hierarchical clustering of normalized and log transformed signal values for genes that were differentially expressed between the various tissue and infection types likewise indicated that the majority of gene expression differences between uninfected animals and mice infected with WT *Y. pestis* CO92 occurred at 48 hours post infection (Figure 2(a)). Moreover, there were some transcripts that represented a “generalized” host response at this latter time point, which is demonstrated in Figure 2(a) by the clustering of all 9 samples representing liver, lung, and spleen replicate samples from mice infected with the WT bacteria (bright red, right-hand side of Figure 2(a)). These higher expressed transcripts were further separated based on tissue type, as expected (Figure 2(a)), indicating that there was a high correlation between replicate samples for these differentially expressed transcripts. A similar concordance was obtained when the signal intensity values from *Y. pestis* CO92 Δ*lpp* mutant-infected tissues were clustered. There were distinct transcriptional changes that characterized the livers (Figure 2(b)), lungs (Figure 2(c)), and spleens (Figure 2(d)) of mice infected with the Δ*lpp* mutant, compared to uninfected animals and animals infected with the WT *Y. pestis* CO92.

The complete list of gene expression alterations in response to infection with WT *Y. pestis* is provided as Supplementary Table 2. Altered genes were mainly associated with immune responses and inflammation. For instance, CD14, several chemokines, INF-*γ*, interleukin 1 receptor, serine peptidase inhibitor, members 3G and 3N of clade A, and genes encoding guanylate binding proteins were observed to increase across the liver, lung, and spleen of WT infected mice at 12 or 48 hours p.i. (Supplementary Table 2). There were 30 genes whose expression was increased in all three tissues (liver, lung, and spleen) in response to WT *Y. pestis* infection, compared to uninfected control animals at 48 hours p.i. (Table 2). These genes also represented mainly immune and stress response functions. Importantly, no genes were altered across all tissue types at 12 hours p.i. with WT *Y. pestis* CO92. Although there were substantial differences in individual gene expression changes observed in the three different tissue types (liver, lung, and spleen) in response to WT *Y. pestis* infection (Figure 2(a)), overall functional processes, as determined using Ingenuity pathway analysis software, were remarkably similar (Figure 3).

### 3.2. Gene Expression Profiling of the Liver from WT *Y. pestis* CO92-Infected Mice

A total of 72 genes were altered in expression (33 upregulated and 39 downregulated) in the livers of mice infected with WT *Y. pestis* at 12 hours p.i. (Table 1). Upregulated genes were mainly those involved in stress and acute-phase responses, signal transduction, and regulation of various metabolic processes, while downregulated genes included those involved in the regulation of cell proliferation and differentiation, apoptosis, and immune cell activation. Contrary to what was observed at the earlier time point, there were a substantial number (1,407) of genes altered by WT *Y. pestis* infection in the liver of mice at 48 hours p.i. (966 upregulated and 441 downregulated, Table 1). Based on the KEGG report obtained using GeneSifter (Supplementary Table 3), the signaling pathways with which upregulated genes were significantly associated included those important for immune response signaling, cell adhesion, apoptosis, and stress responses. Downregulated genes were mainly those involved in various metabolic processes.

### 3.3. Gene Expression Profiling of the Lung from WT *Y. pestis* CO92-Infected Mice

A total of 37 different genes were upregulated in response to WT *Y*. *pestis* in the lungs at 12 hours p.i. compared to uninfected mice. These genes included those that code for several chemokines (e.g., Ccl20, Ccl9, Cxcl1, Cxcl2, Cxcl5, and IL6), stress/acute-phase molecules (e.g., Orm2, Serpina3n, Saa1, Saa3, Gclm, Hspa1a, and Srxn1), and regulators of cell cycle progression and apoptosis (e.g., Cdkn1a, Nupr1, and MafF) (Supplementary Table 2). We noted 11 genes, including cysteine rich protein 61 and gene encoded D site albumin promoter binding protein (Dbp), whose expression was downregulated in response to WT *Y*. *pestis* in the lung at 12 hours p.i.. At 48 hours p.i., 192 genes were altered in the lungs of mice in response to infection with WT *Y. pestis* CO92 (Table 1). Similar to what was observed at 12 hours p.i., the vast majority of altered genes were upregulated (162 genes), and comparatively fewer genes were downregulated (30 genes). Upregulated genes were mainly those involved in immune and acute-phase responses, inflammation, cell cycle regulation, and apoptosis (Supplementary Table 2).

### 3.4. Gene Expression Profiling of the Spleen from WT *Y. pestis* CO92-Infected Mice

A total of 48 genes (44 upregulated and 4 downregulated) were significantly altered in the spleens of mice 12 hours p.i. with WT *Y. pestis*, compared to uninfected control animals. These genes were involved in the regulation of transcription, cell growth and differentiation, and immune-specific functions (e.g., CD69, Cxcr4, Igh-6, and Src-like adaptor protein, Supplementary Table 2). At 48 hours, 77 genes were significantly upregulated and only 1 downregulated in the spleens of mice infected with the WT *Y. pestis* CO92 compared to uninfected control animals (Supplementary Table 2). Many of the upregulated genes seen at 48 hours, compared to uninfected control, were due to the host INF-*γ* response, as evident by Ingenuity pathway analysis of upregulated genes (Figure 3). Specifically, there were 18 transcripts (e.g., CXCL6, IL-1*β*, and IL1R2), in addition to IFN-*γ* itself, that directly participate in IFN-*γ* signaling and were statistically upregulated in WT *Y. pestis*-infected mouse spleens. The mitogen-activated protein kinases ERK1/2 and JNK, transcription factors NF-*κ*B, CREB, and Akt, and apoptosis-associated caspases, all of which are typically regulated via nontranscriptional mechanism (e.g., phosphorylation), are integral components of IFN-*γ* signaling and were thus likely activated (Figure 3).

### 3.5. Comparison of WT *Y. pestis* CO92-Infected Mice to a Previous Study Utilizing a Strain (*Y. pestis* Strain 201) That Is Avirulent in Humans

**Figure 1:**
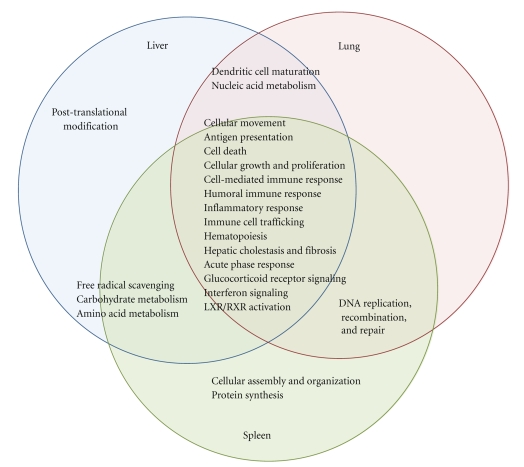
Venn diagram showing the overlap of major functions of genes identified as significantly altered in the liver, spleen, and lung of mice infected with WT *Y. pestis* CO92. Functions were obtained using Ingenuity software, with genes identified at 12 hours or 48 hours in each tissue type analyzed separately. Fisher's Exact Test was used as the scoring method for determining significance of overrepresented molecular functions and pathways.

**Figure 2:**
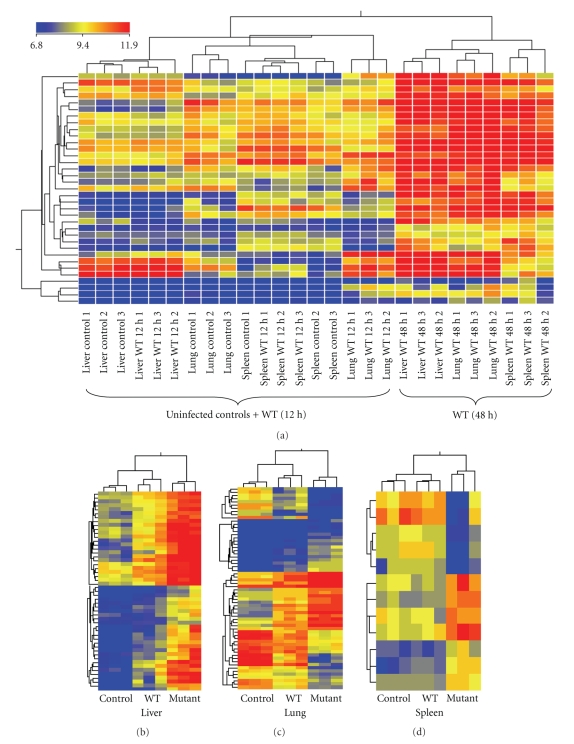
Hierarchical clustering of genes determined to be significantly altered in mice at 12 hours and 48 hours in response to WT *Y. pestis* CO92 infection (a) and in the livers (b), lungs (c), and spleens (d) of mice infected for 48 hours with a Δ*lpp* mutant of *Y. pestis* CO92, compared to WT *Y. pestis*. Clustering was performed using Genespring GX 10.0 on normalized and log transformed signal ratios. The three replicate samples representing the three experimental conditions (uninfected animals and mice infected with WT *Y. pestis* CO92 or its Δ*lpp* mutant) are labeled as control, WT, and mutant, respectively. Note that mice infected for 48 hours with WT *Y. pestis* CO92 exhibited a collection of altered gene expressions that were common to all three tissues examined (panel a). Most notably, the three replicates representing tissue (liver, lung, or spleen) infected with the mutant clustered together, and mutant-infected samples clustered apart from uninfected controls and mice infected with WT *Y. pestis* for each tissue examined (panels b–d). The vertical dendrograms indicate relative similarity between samples (columns), while the horizontal dendrograms indicate clusters for genes (rows). Bright red indicates the highest normalized intensity value, bright blue the lowest, and yellow represents median values.

**Figure 3:**
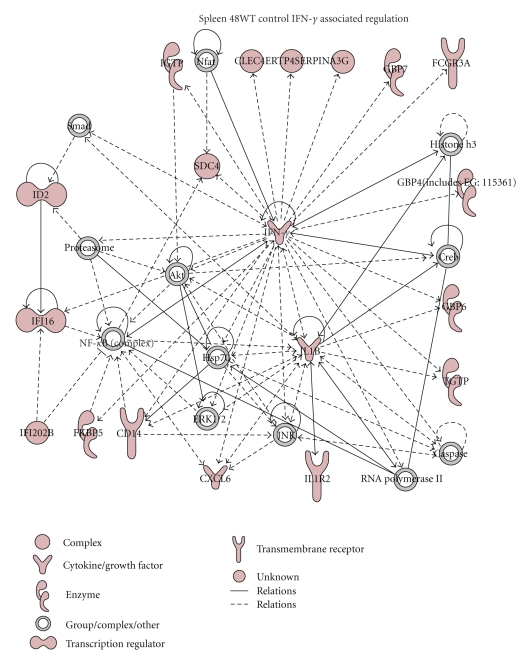
A graphical representation of the changes observed in transcriptional profiles of WT *Y. pestis* CO92-infected mouse spleens at 48 hours post infection. Genes or gene products are represented as nodes and the biological relationship is represented as a line. All lines are supported by at least one reference from literature, textbook, or from canonical information stored in the Ingenuity Pathways Knowledge Base. The red color indicates transcriptional upregulation, based on microarray results. Those signaling molecules which were not colored (e.g., NF-*κ*B complex) were not transcriptionally altered; however, microarray data suggested they were activated non-transcriptionally.

Our study is the first to examine host global transcriptional responses to *Y. pestis* CO92 using an inhalation mouse model. However, Liu et al. [21] performed a similar study in Balb/c mice using a strain that is highly virulent in mice but not humans (*Y. pestis* strain 201). Because the entire gene expression data sets were not made publically available, we performed a comparison of the published results, which consisted primarily of cytokines and cytokine-related signaling molecules in order to gain insight into the potential differences and similarities in most responses to these two different strains. Despite the fact that different mice, array platforms, and analysis methods were employed, the majority of genes reported by Liu et al. as altered in response to infection with *Y. pestis* stain 201 were also identified as altered in our experiments (Table 3). However, there were some fundamental differences that could be contributed by the differences in virulence of the two strains. Most notably, all three tissue types (liver, lung, and spleen) responded similarly to infection with WT *Y. pestis* CO92 (Figure 2), particularly when major functional processes were considered (Figure 1), rather than individual gene alterations. However, some genes were altered differently depending on tissue type in mice infected with *Y. pestis* strain 201. Cd9, for instance, which was upregulated (2.8-fold) in our study in the livers of WT *Y. pestis* CO92-infected mice was upregulated in the liver (2.4-fold) and downregulated (−2.6-fold) in the lung of mice infected with *Y. pestis* strain 201 [21]. Although Cd9 was not deemed significantly altered in our study in the lung or spleen, it was upregulated on average in both tissues (1.5-fold, and 2.1-fold, resp., data not shown). Likewise, Icam2 was reported as upregulated (1.9-fold) in the liver and downregulated (−3.1-fold) in the lung of mice infected with *Y. pestis* strain 201, whereas we found Icam2 to be upregulated (2.8-fold) in the liver (Table 3) and unaffected in the lung in response to infection with WT *Y. pestis* CO92 (data not shown). Most notably, IFN-*γ*, which we found to be upregulated in the liver, spleen, and lung of WT *Y. pestis* CO92-infected mice (Supplementary Tables 2 and 3) and also identified as a critical signaling pathway based on Ingenuity analysis of the entire gene expression dataset (Figure 3), was reported as downregulated in Balb/c mouse lungs in response to infection with the 201 strain [21].

While our study is the first to examine the entire host transcriptome in response to WT *Y. pestis* CO92, Lathem et al. previously performed a cytokine analysis of lung homogenates from C57BL/6 mice that were infected with *Y. pestis* CO92 via the intranasal route [3]. They found that WT *Y. pestis* infection induced upregulation of IL12p70, TNF, IFN-*γ*, MCP-1 (also called CCL2), and IL-6. As shown in Supplementary Table 2, we also detected statistically significant upregulation of IFN-*γ* (4.3-fold), MCP-1 (also called CCL2, 3.4-fold), and IL-6 (34.3-fold) in the lung of WT *Y. pestis*-infected mice. Likewise, we detected an increase in IL-12a (2.6-fold), IL12b (1.9-fold), and TNF-*α* (1.9-fold), although these differences were not deemed statistically significant (data not shown).

### 3.6. Gene Expression Profiling of the Liver, Lung, and Spleen of Mice Infected for 12 Hours with the Δlpp Mutant of *Y. pestis* CO92

For each experimental infection with WT *Y. pestis*, an experiment was also performed using the *Y. pestis* Δ*lpp* mutant to determine the contribution of bacterial Lpp to host transcriptional responses. Based on a direct comparison of WT *Y. pestis*-infected mice and animals challenged with the Δ*lpp* mutant, very few gene expression differences were observed at 12 hours p.i. (Table 1). In the liver of infected mice 12 hours p.i., the Δ*lpp* mutant induced upregulation of 5 transcripts and downregulation of 6 transcripts, most of which were bacterial metabolic genes included on the array as controls (i.e., 28 probe sets representing 6 different genes, Supplementary Table 4). These alterations most likely represented differences in bacterial load at 12 hours in the livers of mice infected with the Δ*lpp* mutant compared to WT *Y. pestis*, which is consistent with histopathological analysis of liver tissue [20]. On the host side, 4 genes were upregulated in response to infection with the Δ*lpp* mutant but not WT *Y. pestis*, including apoptosis inhibitor 5 (Api5), which suggests that Lpp might influence the host apoptotic response to infection (Table 4) and is consistent with our recently published data [22].

No genes were detected as differentially expressed in the lungs of mice infected for 12 hours with the Δ*lpp* mutant, compared to WT *Y. pestis* (Table 1). In contrast, transcriptional differences in the spleen between WT *Y. pestis*-infected and Δ*lpp*-challenged mice were limited to the earlier time point (i.e., differences were observed at 12 hours only). Although these alterations were few (only 25 genes were differential between WT *Y. pestis*-infected and Δ*lpp*-challenged mice), the differences were profound. For instance, 18 probe sets (Affymetrix repeated transcripts) representing 16 different genes that were upregulated in WT-infected mouse spleens were not recapitulated by infection with the Δ*lpp* mutant (Supplementary Table 5). Most of these genes are involved in the regulation of cell growth, including stress-associated cell proliferation (e.g., cyclin D3 and ERBB receptor feedback inhibitor 1). Only one gene was uniquely altered in the absence of Lpp (transthyretin that encodes a prealbumin carrier protein associated with acute phase response [23]), which was upregulated in Δ*lpp* mutant-infected mice, compared to control animals. Of greater interest, immune-specific transcriptional responses (e.g., antihuman CD37 antibody, immunoglobulin kappa chain variable 28, and nemo-like kinase [Nik]) were downregulated in WT Y. *pestis*-infected mice and upregulated in Δ*lpp* mutant-challenged animals, compared to uninfected controls (Table 4). Conversely, apoptosis-associated transcripts (e.g., Bak1 and BCL2L1) were downregulated in WT Y. *pestis*-infected mice and upregulated in Δ*lpp* mutant-challenged mice, compared to uninfected controls (Table 4).

### 3.7. Gene Expression Profiling of the Liver, Lung, and Spleen of Mice Infected for 48 Hours with the Δlpp Mutant of *Y. pestis* CO92

The majority of transcriptional differences in host response to WT *Y. pestis* and the Δ*lpp* mutant occurred at 48 hours p.i. in the liver and lung of infected animals. Most of these represented genes were commonly upregulated or downregulated in both WT *Y. pestis* and Δ*lpp* mutant-infected mice, but by differing magnitudes. For example, CD96 antigen, which is important for macrophage activation and phagocytosis, was upregulated 4.4-fold in the livers of mice infected for 48 hours with WT *Y. pestis* and 11.1-fold in Δ*lpp* mutant-infected mouse livers (Supplementary Table 6). However, there were also more profound differences, in which genes were altered uniquely by either WT *Y. pestis*- or Δ*lpp* mutant-infected mice (Figure 1).

In mouse livers at 48 hours p.i., there were 27 genes that were specifically upregulated in Δ*lpp* mutant-infected mice but not in animals challenged with WT bacteria. Induction of these genes, which included those involved in immune-specific signaling, inflammation, and the regulation of apoptosis, is therefore presumably repressed in the presence of Lpp. There were also 41 genes that were downregulated in the livers of Δ*lpp* mutant-infected mice but not in WT *Y. pestis*-infected animals, compared to control animals (Supplementary Table 6). Two of these genes (i.e., complement factor H-related 1 and V-set immunoglobulin domain containing 4) (Table 4) are involved in the regulation of host immune responses, but the majority are associated with various metabolic processes (e.g., amino acid metabolism and gluconeogenesis).

There were 109 genes that were differentially expressed in the lung between WT *Y. pestis*-infected mice and animals challenged with Δ*lpp* mutant bacteria (Table 1). Seventy of these genes were modestly upregulated (1.5-fold to 34.3-fold, mean = 3.9 ± 4.6) in response to WT *Y. pestis* infection but more profoundly upregulated (3-fold to 172-fold, mean = 19.8 ± 32.4) in response to infection with the Δ*lpp* mutant (Supplementary Table 7). In contrast to what was observed at 12 hours or at 48 hours in liver tissue, the majority of differentially expressed genes in lungs were those critical for immune and stress responses, inflammation, and apoptosis. For instance, IL-6 and CXCL2 (also called Mip-2*α*) were upregulated in the lungs of WT *Y. pestis*-infected mice 34.3-fold and 18.2-fold, respectively, compared to uninfected mice. In Δ*lpp* mutant-infected mice, on the other hand, IL-6 and CXCL2 were upregulated 172-fold and 169.1-fold, respectively (i.e., 5-fold and 9.3-fold larger inductions), compared to uninfected control mice.

A total of 39 genes were upregulated exclusively in the lungs of mutant-infected mice after 48 hours of infection (i.e., not altered in the lungs of the WT *Y. pestis* group of infected mice) (Supplementary Table 7). Most of these genes were associated with apoptosis, inflammation, immune responses, and signaling pathways critical for immune cell activation, including apoptosis regulators Birc3 and Bcl2a1a, CD53, CXCL14 (also called Mip-2*γ*), coagulation factors III and X, IL-22, early growth response 1, leukemia inhibitory factor, and prostaglandin E synthase (Supplementary Table 7).

**Figure 4:**
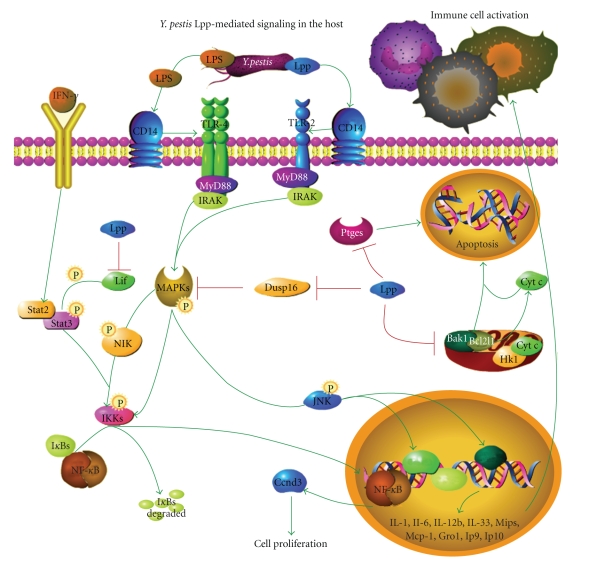
Putative host signaling pathway induced by *Y. pestis* bacterial effectors, LPS and Lpp. TLR-2, TLR-4, CD14, and INF-*γ* were transcriptionally upregulated in host tissues in response to WT *Y. pestis* infection. Binding of TLR-4 by LPS and TLR-2 by Lpp is inferred from literature and canonical pathway databases (e.g., Biocarta). Myd88 and IRAK, which are TLR adaptor molecules, were upregulated by WT. *Y. pestis*, resulting in the activation of mitogen-activated protein kinases (MAPKs), three of which (Map3k6, Map3k8, and Map4k5) were transcriptionally upregulated in WT *Y. pestis*-infected mice based on microarray analysis results. MAPKs are known to phsophorylate and activate nemo-like kinase (Nik), which was upregulated in WT bacteria-infected mice but not in animals infected with the Δ*lpp* mutant. Phosphorylation of Nik is known to cause activation of NF-*κ*B, which was also transcriptionally upregulated in WT *Y. pestis*-infected mice. Engagement of IFN-*γ* to its receptor also leads to NF-*κ*B activation via STAT 2 and 3, which were upregulated in WT *Y. pestis*-infected animals. NF-*κ*B activation results in transcription of proinflammatory cytokines, which were indeed upregulated based on microarray analysis (examples are listed in the diagram). The three MAPKs that were transcriptionally upregulated based on microarray analysis are known activators of c-jun N-terminal kinases (JNK) which leads to activation of Elk-1 and AP-1 transcription factors. AP-1 is composed of c-Jun and Fos subunits, both of which were upregulated in WT infected mice. Leukemia inhibitory factor (Lif) was uniquely upregulated in mice infected with the mutant and thus is likely inhibited in the presence of *lpp*, as shown. Cyclin D3 (Ccnd3) was uniquely upregulated in the spleen of WT-infected mice and the lung of Δ*lpp* mutant infected mice and leads to increased cell proliferation. Prostaglandin E synthase (Ptges), Bak1, Bcl2l1, and hexokinase 1 (Hk1) are all known to regulate apoptosis. Each of the genes encoding these proteins was differentially expressed in Δ*lpp* mutant-infected animals, compared to those infected with WT *Y. pestis*. Most likely, Lpp contributes to inhibition of host cell apoptosis and modulates inflammatory responses in coordination with LPS [22].

Based on the transcriptional profiles of liver, lung, and spleen of mice, the most profound differences between animals infected with WT *Y. pestis* versus the Δ*lpp* mutant, literature searches, and known signaling pathways available in various online databases (e.g., NCBI, Biocarta, and The Protein Lounge), we created a putative Lpp-associated signaling pathway (Figure 4). For instance, we inferred that the most likely pathway for the production of the multiple cytokines that were identified as increased based on microarray results is phosphorylation and activation of NF-*κ*B and JNK *via* TLR-2 and TLR-4 induced activation of mitogen-activated protein kinases (MAPKs) (Figure 4). IFN-*γ* signaling, which is peripherally associated with this same pathway, was also inferred to be activated in response to WT *Y. pestis* and would possibly complement LPS signaling *via* TLR-4 to explain the induction of cytokines, albeit somewhat blunted, in the absence of the *lpp* gene (Figure 4). Reduction in some host responses, identified as expression alterations in Δ*lpp* mutant-infected animals, could be partially explained by Lpp-mediated inhibition of leukemia inhibitory factor (Lif) and Dusp16 (Table 4), which downregulate activation of NF-*κ*B and JNK, respectively. The most directly affected process, based on WT versus Δ*lpp*-infected animals, was apoptosis, possibly *via* inhibition of prostaglandin E synthase (Ptges) (Table 4) [24, 25] and perturbation of relative ratios of mitochondrial factors (e.g., Bcl-2 family members) (Figure 4). Overall, the three main signaling pathways induced by WT *Y. pestis* were TLR-4, TLR-2, and INF-*γ* signaling, which culminated in the production of multiple inflammatory cytokines, also detected as upregulated in infected mice in all three tissues examined.

## 4. Discussion

In the present study, a transcriptional ontological assessment of significantly modulated genes in the liver, lung, and spleen from WT *Y. pestis* CO92-infected mice revealed a number of up- and downregulated transcripts that were associated with immune mechanisms. For example, an increase in CD14 transcript was observed across liver, lung, and spleen of mice infected with WT *Y. pestis* CO92 at 48 hours p.i., but the gene encoding IL-10 was not upregulated. CD14 exists as a membrane-bound or soluble form and serves as a coreceptor with TLRs or LPS-binding protein to associate with LPS from Gram-negative bacteria [26]. During *Y. enterocolitica* infection, CD14 complexes with TLR-2 on macrophages and subsequently binds low calcium response antigen V (LcrV), which leads to a reduction in TNF-*α* and an increase in IL-10 [27]. This IL-10 induction by LcrV through binding to TLR-2/CD14 plays a key role in *Y. enterocolitica* immune evasion and pathogenicity [27]. However, previous studies on *Y. pestis* indicated that IL-10 was not produced in the lungs of mice infected intranasally, and TLR-dependent IL-10 induction by LcrV did not contribute to the virulence of *Y. pestis* [28]. Our results are consistent with these findings and suggest that IL-10 suppression might be an important virulence mechanism for enteropathogenic yersiniae.

The majority of transcriptional alterations identified in the liver, lung, and spleen of WT *Y. pestis* mice were those important for host immune responses, as expected. In addition to CD14, TLR-4 and TLR-2 were upregulated p.i. (2.6- and 9.2-fold, resp., Supplementary Table 2), as were several downstream targets of these two TLR signaling pathways (Figure 4). We also identified the IFN-*γ* signaling pathway as a central player in the host response to WT *Y. pestis* infection (Figure 3). INF-*γ*, which was induced at 48 hours p.i. in all of the tissues, is produced by activated natural killer cells and T cells and is critical for a successful immune response to intracellular pathogens [29–31]. Also upregulated in WT *Y. pestis*-infected mice were the IFN-*γ*-regulated serine proteases Serpina3g and Serpina3n (Supplementary, Table 2), which can inhibit caspase-independent death [32] and assist in the development of memory CD8 T cells [33]. Likewise, we noted WT *Y. pestis*-induced upregulation of suppressor of cytokine signaling 1 (*socs1*, 8-fold in the liver) and *socs3* (4-15-fold in all three tissues), which regulate JAK-STAT signaling, and TNF-*α*-induced protein 3 (*tnfaip3b*, 2.7-15.4-fold in the liver and lung), which is essential for negative regulation of I-*κ*B kinase/NF-*κ*B cascade (Supplementary Table 2).

Other IFN-*γ*-regulated molecules that were induced in response to WT *Y. pestis* infection included several guanylate binding proteins (GBP2, 4, 7, and 6/10), which were upregulated in all of the tissues collected from WT-infected mice. This IFN-*γ*-induced family of proteins has been poorly characterized, but they have been shown to regulate endothelial cell proliferation during infection, possibly by slowing cell-to-cell spreading [34]. IGTP (IRGM3) and TGTP (IRGB6), members of the p47 GTPases family, were also increased in all of the tissues of WT bacteria-infected mice (Supplementary Table 2). These molecules are similar to the GBPs but do not require *de novo* synthesis of transcription factors [35]. Functionally, they have been shown to localize to infected vacuoles in a *Toxoplasma gondii* infection [36], which is followed by vesicle formation, disintegration of the vacuole, and the subsequent demise of the parasites [37]. Consequently, these guanylate binding proteins could perform a similar function during *Y. pestis* intracellular infection.

Consistent with a strong host inflammatory response to infection, multiple cytokines and chemokines were upregulated in WT *Y. pestis*-infected animals in all three tissues examined (Supplementary Tables 2 and 3). For instance, CXCL10 and CCL2, which were profoundly upregulated (6-30.5-fold) in response to WT *Y. pestis* infection, are chemottractants for monocytes, T cells, and dendritic cells. Likewise, neutrophils, important to the amelioration of early bacteremia, are attracted by CXCL6 [38], which was upregulated in the liver (23.1-fold), lung (6.4-17.5-fold), and spleen (14.6-fold) post infection (Supplementary Tables 2 and 3). Induction of some of these inflammatory chemokines (e.g., CCL3) would specifically attract monocytes, which may benefit *Yersinia* by providing a safe haven for replication [38, 39]. The compendium of host responses identified in this study supports a strong host inflammatory response that culminates in the activation of immune effectors downstream of TLR-2 and TLR-4 and subsequent amplification of the inflammatory responses *via* production of IFN-*γ*.

We noted an upregulation of Lipocalin 2 (Lcn2) from 6.5-fold at 12 hours to 67.3-fold at 48 hours and downregulation of the HFE2 gene (8.1-fold) in the livers of WT-infected mice at 48 hours p.i. **(**Supplementary Table 2). Both Lcn2 and HFE2 are associated with iron regulation, and mutation in the HFE2 gene is causative for hematochormasis, which is characterized by iron overload [40]. The increase in Lcn2 by WT bacteria in the liver might check bacterial growth by binding to siderophores and could be a mechanism of mediating innate immune response. No change in its level, as observed in the Δ*lpp* mutant, would cause normal bacterial growth in the liver. Based on our recent results (19), the Δ*lpp* mutant grew normally in liver but not in the spleen or blood.

In conjunction with our assessment of host transcriptional responses in WT *Y. pestis*-infected mice, we also investigated the effects of an Δ*lpp* mutant on gene expression. Recently, we demonstrated that in *Y. pestis*, deletion of the *lpp* gene from the *pgm*-locus KIM/D27 background strain further attenuated its virulence. However, minimal differences were noted in pathogenicity between the WT- and the Δ*lpp-*mutant strain of CO92 in a pneumonic plague mouse model, probably because* Y. pestis* CO92 strain is highly virulent and deletion of one gene causes only increases in mean time to death [20, 41]. Interestingly, when groups of mice infected with either the WT CO92 or its Δ*lpp* mutant were given a subinhibitory dose of levofloxacin, we observed a significantly higher survival rate, less severe histopathological changes, and reduced cytokine/chemokine levels in the Δ*lpp* mutant-infected group compared to WT-infected mice [20]. These data indicated that Lpp contributed to virulence of *Y. pestis* CO92 and was dependent on bacterial load. We used an intranasal mouse model of infection to study host gene expression alterations in the liver, lung, and spleen at 12 hours and 48 hours p.i. that demonstrates the distinctions of virulence and pathogenic mechanism(s) between WT and Δ*lpp* mutant strains of *Y. pestis* CO92 in a pneumonic plague model.

Our first observation of mice infected with the Δ*lpp*-mutant strain of *Y. pestis* CO92, compared to WT-infected animals, was that transcriptional responses that could be due to TLR-4 activation *via* LPS (e.g., chemokines, JAK-STAT signaling molecules, etc.) were blunted in the absence of *lpp* gene expression (Supplementary Tables 4–7), which supports a synergistic role for Lpp and LPS to induce septic shock as well as the LPS-like signaling previously observed in an LPS-nonresponsive background strain of mice [18]. More interesting were transcriptional responses that were completely perturbed in the absence of *lpp*, such as activation in WT *Y. pestis*-infected animals but not in those infected with the Δ*lpp*-mutant. These results provided much greater insight into Lpp-specific host signaling in the context of *Y. pestis* infection and allowed us to propose a putative signaling pathway (Figure 4) that could explain the intertwined roles of LPS and Lpp and also how *Y. pestis* might survive inside host cells.

As shown in Figure 4, WT *Y. pestis* induces the upregulation of TLR-4, TLR-2, and CD14 independently of Lpp (i.e., these molecules were also upregulated in mice infected with the Δ*lpp*-mutant). However, the LPS and Lpp share a common downstream signaling pathway, and even in the absence of Lpp, these intermediate inflammatory effectors (e.g., Myd88, IRAK, mitogen activated kinases, STATs, NF-*κ*B, c-Jun and Fos, and various proinflammatory cytokines) were increased during *Y. pestis* infection (Supplementary Table 2 and Figure 4). Nontranscriptional events (e.g., Nik-mediated phosphorylation of IKK and subsequent degradation of I*κ*Bs and nuclear location of NF-*κ*B) that are likely to have occurred based on the transcriptional profiles of *Y. pestis*-infected mice and classical signaling pathways are included for clarity. In the context of this WT model of infection, Lpp-specific signaling events were also apparent. Nik, for instance, is a crucial regulatory point downstream of TLR and cytokine receptor engagement, and its upregulation in WT *Y. pestis* infected mice was not recapitulated when the Δ*lpp*-mutant was used. Other mechanisms of I*κ*B phosporylation and degradation would presumably occur in the absence of Lpp, since proinflammatory cytokines are still produced in the absence of the *lpp* gene.

Cell death was a major process identified as statistically overrepresented in all three tissue types, based on Ingenuity pathway analysis of altered genes (Figure 3). The balance of proapoptotic and antiapoptotic factors often determines cell fate, and apoptosis regulators can also function differently depending on cell type. Its regulatory complexity makes apoptosis-related transcriptional responses difficult to interpret. However, the absence of the *lpp* gene clearly perturbed the effects of the WT *Y. pestis* infection by subtly altering some apoptotic related transcription responses and specifically inducing or depressing others. For instance, the expressions of two genes encoding for Bcl2 family proteins (Bak1 and Bcl2l1) that function to induce apoptosis [42] were suppressed in the spleen of WT-infected mice but not in animals infected with the Δ*lpp*-mutant (Table 4). Likewise, Hk1 was uniquely downregulated in only WT-infected mice, suggesting that its suppression requires the presence of bacterial Lpp. Whereas suppression of Bak1 and Bcl2l1 would likely be cytoprotective, cytochrome *c* release is inhibited by Hk1 [43], and therefore its decrease could lead to increased apoptosis [22].

## 5. Conclusions

This study provided the first comprehensive assessment of the host transcriptional profile in the lung, liver, and, spleen of mice intranasally infected with a highly virulent strain of *Y. pestis* CO92. We further investigated the contributions of bacterial Lpp to host transcriptional responses and presented a putative host signaling pathway that plausibly explained the synergistic actions of LPS and Lpp in the context of *Y. pestis* infection. Our results supported a model in which *Y. pestis* induced a strong inflammatory response, mediated by both LPS and Lpp, but evaded immune clearance, possibly by Lpp-induced inhibition of host cell apoptosis.

## Supplementary Material

Supplementary Table I shows “Experimental summary.”
Supplementary Table II shows “Genes statistically differentially expressed in the livers, lungs and spleens of mice infected with WT *Y. pestis* CO92, compared to uninfected mice.”
Supplementary Table III shows “KEGG pathways most significantly associated with genes altered in the livers of WT *Y. pestis*-infected mice at 48 hr post-infection.”
Supplementary Table IV shows “Genes statistically differentially expressed in the livers of mice infected for 12 hr with a Δ*lpp* mutant of *Y. pestis* CO92, compared to WT Bacteria.”
Supplementary Table V shows “Genes statistically differentially expressed in the spleens of mice infected for 12 hr with a Δ*lpp* mutant of *Y. pestis* CO92, compared to WT Bacteria.”
Supplementary Table VI shows “Genes statistically differentially expressed in the livers of mice infected for 48 hr with a Δ*lpp* mutant of *Y. pestis* CO92, compared to WT Bacteria.”
Supplementary Table VII shows “Genes statistically differentially expressed in the lungs of mice infected for 48 hr with a Δ*lpp* mutant of *Y. pestis* CO92, compared to WT Bacteria.”

## Figures and Tables

**Table 1:** Summary of altered gene expression in mice infected with WT *Y. pestis* CO92, or its Δ*lpp* mutant, compared to uninfected control animals.

Tissue	Control versus WT				WT versus Mutant			
	12 hours		48 hours		12 hours		48 hours	
	Up	Down	Up	Down	Up	Down	Up	Down
	Number of Genes							

Liver	33	39	966	441	5	6	120	136
Lung	37	10	162	30	—	—	109	—
Spleen	44	4	77	1	9	16	—	—

A dash (“—") indicates that no gene expression differences were deemed as statistically significant (fold-change ≥1.5, *P* value ≤.05). Note that ∼57% (109 out of 192) of WT *Y. pestis*-induced host transcriptional responses in the lungs of mice infected for 48 hours were perturbed in the absence of *lpp*.

**Table 2:** Genes that were commonly altered in the liver, lung, and spleen of mice infected with WT. *Y. pestis* CO92 for 48 hours.

GenBank ID	Gene Name	Function	Control versus WT *Y. pestis* CO92 at 48 hours		
			Liver	Lung	Spleen
			FC		
AV309418	N-myc downstream regulated 1 (Ndrg)	Regulation of progression through cell cycle; inflammatory response; immune response	23.1	17.5	14.6
BM234360	Fibronectin 1 (Fn1)	Acute-phase response	53	29.4	6.7
BC010337	Keratin 7 (Krt7)	Cytokine and chemokine mediated signaling pathway	2	7.3	6.5
AY061760	Nuclear factor, interleukin 3, regulated (Nfil3)	Electron transport; fat cell differentiation	7.5	3.7	6.1
AK013239	Inhibitor of DNA binding 2 (Id2)	Electron transport; cell motility; chemotaxis; inflammatory response; immune response; cell surface receptor linked signal transduction; cell-cell signaling; muscle development; circulation; positive regulation of cell proliferation	30.5	20	6
AK013765	Endothelial cell growth factor 1 (platelet-derived) (Ecgf1)	Immune response	22.4	4.6	5.5
BG064671	A disintegrin-like and metalloprotease (reprolysin type) with thrombospondin type 1 motif, 4 (ADAMTS4)	Regulation of cell growth; neutrophil apoptosis; inflammatory cell apoptosis; cell motility; immune response; response to virus; antigen processing and presentation; neutrophil chemotaxis; unfolded protein response; negative regulation of myelination; defense response to bacterium; positive regulation of chemokine biosynthesis	4.2	4.3	5.4
AK004893	Inter alpha-trypsin inhibitor, heavy chain 4 (Itih4)	Chemotaxis; inflammatory response; immune response; signal transduction	8.8	9.3	5.3
NM_031192	Renin 1 structural (Ren1)	Regulation of cell growth; regulation of protein amino acid phosphorylation; antiapoptosis; JAK-STAT cascade; negative regulation of insulin receptor signaling pathway	15	4	5.3
NM_008326	Immunity-related GTPase family, M (Irgm)	Immune response	6.1	7.9	5.1
AW546137	Bone morphogenic protein receptor, type II (serine/threonine kinase) (Bmpr2)	Response to stress; stress-activated protein kinase signaling pathway	2.8	2.2	5.6
AV328143	ADP-ribosylation factor-like 4A (Arl4a)	Unknown	23.7	10.4	4.3
NM_011722	Dynactin 6 (Dctn6)	Immune response; cell surface receptor linked signal transduction	3.7	5.2	4.2
BB667823	Ring finger protein 125 (Rnf125)	Immune response; response to virus	11.1	7.2	4.1
BB561053	Zinc finger, MYND domain containing 11 (Zmynd11)	Acute-phase response	3.6	11.9	4
AF004023	Cd200 antigen (Cd200)	Chemotaxis; inflammatory response; immune response; signal transduction	2.7	3.3	3.8
BC025819	Cytochrome P450, family 2, subfamily c, polypeptide 44 (Cyp2c44)	Immune response; cell adhesion; antimicrobial humoral response	2.5	2.3	3.8
AK006551	Coenzyme Q10 homolog B (Coq10b)	Apoptosis; immune response	20.9	11.6	3.7
NM_011016	Orosomucoid 2 (Orm2)	Immune response	7.4	2.9	3.5
AF022957	Acidic (leucine-rich) nuclear phosphoprotein 32 family, member A (Anp32a)	Somitogenesis; response to biotic stimulus; anterior/posterior pattern formation	3.3	2	3.1
NM_023462	Retinol binding protein 7, cellular (Rbp7)	Immune response	4	4.3	3
BB223018	Schlafen 1 (Slfn1)	Electron transport; lactation; regulation of epithelial cell differentiation	3.5	3.5	2.8
BG076338	F-box protein 21 (Fbxo21)	Immune response	11.1	4.2	2.7
NM_008458	Serine (or cysteine) peptidase inhibitor, clade A, member 3C (Serpina3c)	Regulation of progression through cell cycle; fever; apoptosis; inflammatory response; immune response; cell proliferation; regulation of cell proliferation; antimicrobial humoral response; neutrophil chemotaxis; positive regulation of chemokine biosynthesis; positive regulation of IL-6 biosynthesis; leukocyte migration	7.2	7.1	2.5
AF047725	Cytochrome P450, family 2, subfamily c, polypeptide 38 (Cyp2c38)	Unknown	2.5	2.6	2.5
BC018333	Aldo-keto reductase family 1, member D1 (Akr1d1)	NAD biosynthesis; pyridine nucleotide biosynthesis	4.1	2.4	2.5
BC021340	Poly (ADP-ribose) polymerase family, member 14 (Parp14)	Immune response	4	7.8	2.4
BB326709	Doublecortin and CaM kinase-like 3 (Dcamkl3)	Protein folding	2.4	8.1	2.4
AF030178	Phosphatidylinositol glycan anchor biosynthesis, class Q (Pigq)	Cytokine and chemokine mediated signaling pathway	3.8	5.4	2.3
NM_008262	One cut domain, family member 1 (Onecut1)	Phagocytosis; apoptosis; inflammatory response; immune response; cell surface receptor linked signal transduction	30.9	4.3	2.3

Fold changes (FCs) shown were statistically significant (i.e., Benjamini and Hochberg corrected *P* value <.05 and absolute consistency across all replicate experiments). A negative sign before values indicates downregulation.

**Table 3:** Comparison of transcriptional alterations in mice infected with WT *Y. pestis* CO92 (this study) and a strain that is avirulent in humans (*Y. pestis* strain 201, previous study reported by Liu et al. [21]).

This study			Liu et al.		
Gene	FC	Tissue	Gene	FC	Tissue
CD34	3.7	Liver	CD34	2.2	Liver
Ifitm1	3.3	All	Ifitm3	2.7	Liver
Ifitm6	4.4	Lung			
Tgfbr2	2.3	Liver	Tgfb3	2.5	Liver
				−3	Lung
Tnfsf10	2.3	Liver	Tnfrsf1a	2.3	Liver
			Tnfrsf13b	2.3	Lung
Ccl20	2.4	Lung	Ccl20	7.5	Lung
Cd14	12.5	All	Cd14	14.1	Lung & Liver
Csf3	6.6	All	Csf3	5	Lung
Cxcl2	39.8	Lung & Liver	Cxcl2	14.2	Lung & Liver
Cxcl5/6	9.5	All	Cxcl5/6	2.6	Lung
IL-1b	5.6	All	IL-1b	4.8	Lung
IL2rg	2.9	Liver	IL2rb	2.5	Lung
Tnfaip8	2	Liver	Tnfaip8	2.3	Lung
Cd9	2.8	Liver	Cd9	2.4	Liver
				−2.6	Lung
Icam2	2.8	Liver	Icam2	1.9	Liver
				−3.1	Lung
IFN-*γ*	4.6	All	IFN-*γ*	−2.2	Lung
Igfbp1	3.7	Liver	Igfbp1	3.2	Liver
			Igfbp4	2.1	
Lbp	4.2	Liver	Lbp	4.2	Liver
Ndrg	−2	Liver	Ndrg1	Up	?
Gadd45g	8.3	Liver	Gadd45g	Up	?
MT1	5.1	Lung & Liver	MT1	Up	Lung & Liver
MT2	9.2	Lung & Liver	MT2	Up	Lung & Liver

For this study, fold changes (FCs) shown are averages across affected tissues. Only alterations that were determined to be statistically significant (i.e., Benjamini and Hochberg corrected *P* value <.05 and absolute consistency across all replicate experiments) are included. A negative sign before values indicates downregulation.

**Table 4:** Selected gene expression alterations in the liver, lung, and spleen of mice infected with a Δ*lpp* mutant of *Y. pestis* CO92, compared to WT *Y. pestis*.

GenBank ID	Gene Name	WT versus Mut	C versus WT	C versus Mut
			FC	
Liver 12 hours				

K01391	B.subtilis tryptophan (trp) operon, complete cds.	25.9	—	25.9
BB449248	Apoptosis inhibitor 5 (Api5)	3.0	—	2.8
L38424	Bacillus subtilis dihydropicolinate reductase (jojE)	−167.8	—	−226.1

GenBank ID	Gene Name	WT versus Mut	C versus WT	C versus Mut

Spleen 12 hours				

BB167641	Cyclin D3 (Ccnd3)	−4.2	3.8	—
BC027056	Microtubule-associated protein, RP/EB family, member 2 (Mapre2)	−2.6	2.0	—
BB636266	Nemo like kinase (Nlk)	−2.3	2.1	—
NM_013743	Pyruvate dehydrogenase kinase, isoenzyme 4 (Pdk4)	−2.7	3.1	—
AF402617	BCL2-antagonist/killer 1 (Bak1)	3.1	−2.2	—
NM_009743	Bcl2-like 1 (Bcl2l1)	2.4	−2.3	—
NM_010438	Hexokinase 1 (Hk1)	4.8	−4.8	—
AY058908	Anti-human CD37 antibody WR17 kappa light chain variable region	4.0	−2.4	1.6
L41881	Immunoglobulin kappa chain variable 28 (V28) (Igk-V28)	3.8	−1.8	2.1
BG141874	Transthyretin (Ttr)	3.1		3.2

Liver 48 hours				

AK014526	Lipin 1 (Lpin1)	−1.8	2.3	
BE685667	Cyclin D3 (Ccnd3)	2		2.1
AF169388	Procollagen, type IV, alpha 4 (Col4a4)	2		3.0
BB305930	Protocadherin 17 (Pcdh17)	2.2		3.0
BM122301	Transforming growth factor, beta receptor III (Tgfbr3)	1.8		2.6
BC025105	V-set and immunoglobulin domain containing 4 (Vsig4)	−2.3	—	−2.8
NM_015780	Complement factor H-related 1 (Cfhr1)	−2.9		−3.4

Lung 48 hours				

BC011338	Baculoviral IAP repeat-containing 3 (Birc3)	1.83	—	2.2
L16462	B-cell leukemia/lymphoma 2 related protein A1a (Bcl2a1a)	2.34	—	3.5
NM_007651	CD53 antigen (Cd53)	1.99	—	2.1
AF252873	Chemokine (C-X-C motif) ligand 14 (Cxcl14)	2.71	—	2.7
BC024886	Coagulation factor III (F3)	2.18	—	2.4
NM_007972	Coagulation factor X (F10)	2.41	—	3
BM238701	Dual specificity phosphatase 16 (Dusp16)	2.63	—	2.7
NM_007913	Early growth response 1 (Egr1)	3.03	—	3
AF065917	Leukemia inhibitory factor (Lif)	3.36	—	4.7
BB491008	MARCKS-like 1	2.96	—	3.6
BB730139	Prostaglandin E synthase (Ptges)	2.55	—	3

Fold changes (FCs) shown were statistically significant (i.e., Benjamini and Hochberg corrected *P* value <.05 and absolute consistency across all replicate experiments). A negative sign before values indicates downregulation. A dash “—" indicates that no statistical difference was observed. WT and mutant (Mut) refer to wild-type *Y. pestis* CO92 and its Δ*lpp* mutant, respectively. C: control (uninfected) mice.
