# Supplementary material for: Comparative Analyses of Transcriptional Profiles in Mouse Organs Using a Pneumonic Plague Model after Infection with Wild-Type Yersinia pestis CO92 and Its Braun Lipoprotein Mutant
Source: Comp Funct Genomics. 2010 Jan 20;2009:914762. doi: 10.1155/2009/914762 (PMC2817383; doi:10.1155/2009/914762)
Supplement: Supplementary file 1 — Supplementary Table I shows “Experimental summary.” Supplementary Table II shows “Genes statistically differentially expressed in the livers, lungs and spleens of mice infected with WT Y. pestis CO92, compared to uninfected mice.” Supplementary Table III shows “KEGG pathways most significantly associated with genes altered in the livers of WT Y. pestis-infected mice at 48 hr post-infection.” Supplementary Table IV shows “Genes statistically differentially expressed in the livers of mice infected for 12 hr with a Δlpp mutant of Y. pestis CO92, compared to WT Bacteria.” Supplementary Table V shows “Genes statistically differentially expressed in the spleens of mice infected for 12 hr with a Δlpp mutant of Y. pestis CO92, compared to WT Bacteria.” Supplementary Table VI shows “Genes statistically differentially expressed in the livers of mice infected for 48 hr with a Δlpp mutant of Y. pestis CO92, compared to WT Bacteria.” Supplementary Table VII shows “Genes statistically differentially expressed in the lungs of mice infected for 48 hr with a Δlpp mutant of Y. pestis CO92, compared to WT Bacteria.” [file 914762.f1.pdf]

Supplementary Content:

Supplementary Table I. Experimental summary

Arrays (3 each)	Treatment	Length of infection	Tissue
Control (C)	Uninfected	-	Liver
WT	WT-infected	12 hours	
Mut	Mutant-infected		
WT	WT-infected	48 hours	
Mut	Mutant-infected		
Control (C)	Uninfected	-	Lung
WT	WT-infected	12 hours	
Mut	Mutant-infected		
WT	WT-infected	48 hours	
Mut	Mutant-infected		
Control (C)	Uninfected	-	Spleen
WT	WT-infected	12 hours	
Mut	Mutant-infected		
WT	WT-infected	48 hours	
Mut	Mutant-infected		

WT=wild-type *Y. pestis* CO92; Mut=Δ*lpp* mutant of *Y. pestis* CO92

Supplementary Table II. Genes statistically differentially expressed in the livers, lungs and spleens of mice infected with WT *Y. pestis* CO92, compared to uninfected mice

GenBank ID	Gene Name	Control vs WT *Y. pestis* CO92					
		Liver		Lung		Spleen	
		12 h	48 h	12 h	48 h	12 h	48 h
		FC					
BE952632	2-4-dienoyl-Coenzyme A reductase 2, peroxisomal (decr2)	-	-2.5	-	-	-	-
BC018470	2'-5' oligoadenylate synthetase 1A (Oas1a)	-	-	-	2.4	-	-
AB067534	2'-5' oligoadenylate synthetase 3 (Oas3)	-	-	-	2	-	-
BQ033138	2'-5' oligoadenylate synthetase-like 2 (Oasl2)	-	5.1	-	-	-	5.2
AK007603	3-hydroxybutyrate dehydrogenase, type 2 (Bdh2)	-	-2.7	-	-	-	-
AV213379	3-oxoacid CoA transferase 1 (Oxct1)	-	3.5	-	-	-	-
BC010837	4-nitrophenylphosphatase domain and non-neuronal SNAP25-like protein homolog 1 (ipsnap1)	-	-2.5	-	-	-	-
NM_133232	6-phosphofructo-2-kinase/fructose-2,6-biphosphatase 3 (Pfkfb3)	-	16.1	-	3.5	-	-
BG064671	A disintegrin-like and metalloprotease (reprolysin type) with thrombospondin type 1 motif, 4 (ADAMTS4)	-	2.4	-	2.6	-	-
NM_031185	A kinase (PRKA) anchor protein (gravin) 12 (Akap12)	-	6.8	-	-	-	-
AI649048	A kinase (PRKA) anchor protein 2 (Akap2)	-	6.4	-	-	-	-
BB317588	AarF domain containing kinase 5 (Adck5)	-	-2.4	-	-	-	-
AV009478	Abhydrolase domain containing 14A (Abhd14a)	-	-2.1	-	-	-	-
NM_025341	Abhydrolase domain containing 6 (Abhd6)	-	-2.5	-	-	-	-
NM_130864	Acetyl-Coenzyme A acyltransferase 1A (Acaa1a)	-	-2.8	-	-	-	-
BC019882	Acetyl-Coenzyme A acyltransferase 1B (Acaa1b)	-	-3.9	-	-	-	-
BB183525	Acid phosphatase-like 2 (Acpl2)	-	-3.4	-	-	-	-
AF022957	Acidic (leucine-rich) nuclear phosphoprotein 32 family, member A (Anp32a)	-	-2.3	-	-	-	-
NM_007392	Actin, alpha 2, smooth muscle, aorta (Acta2)	-	2	-	-	-	-
NM_013798	Actin, gamma, cytoplasmic 1 (Actg1)	-	2.2	-	-	-	-
BC003232	Actinin, alpha 1 (Actn1)	-	2.2	-	-	-	-
BC019946	Activating transcription factor 3 (Atf3)	-	3.2	-	2.5	-	-
BQ043509	Activin A receptor, type 1B (Acvr1b)	-	2.3	-	-	-	-
BC014291	Activin A receptor, type II-like 1 (Acvrl1)	-	2.9	-	-	-	-
BC006692	Acyl-CoA synthetase long-chain family member 1 (Acsl1)	-	-2.8	-	-	-	-
AB078618	Acyl-CoA thioesterase 12 (Acot12)	-	-3.2	-	-	-	-
NM_134246	Acyl-CoA thioesterase 3 (Acot3)	-	-3	-	-	-	-
AA571017	Acyl-CoA thioesterase 4 (Acot4)	-	-2.5	-	-	-	-

NM_022816	Acyl-CoA thioesterase 9 (Acot9)	-	2.6	-	-	-	-
BI790311	Acyl-Coenzyme A binding domain containing 3 (Acbd3)	-	2.1	-	-	-	-
BG068664	Acyltransferase like 2 (Aytl2)	-	2.4	-	-	-	-
BB185861	Adaptor-related protein complex 1, sigma 2 subunit (Ap1s2)	-	2.6	-	-	-	-
BB746807	Adenylate cyclase 7 (Adcy7)	-	2	-	-	-	-
BI653265	ADP-ribosylation factor 4 (Arf4)	-	2.1	-	-	-	-
BG067878	ADP-ribosylation factor GTPase activating protein 3 (Arfgap3)	-	2.2	-	-	-	-
AU043075	ADP-ribosylation factor interacting protein 2 (Arfip2)	-	2.1	-	-	-	-
NM_024191	ADP-ribosylation factor-like 2 binding protein (Arl2bp)	-	2.1	-	-	-	-
AV328143	ADP-ribosylation factor-like 4A (Arl4a)	-2.1	-	-	-	-	-
BB391602	Adrenergic receptor kinase, beta 2 (Adrbk2)	-	2	-	-	-	-
AV083350	Adrenergic receptor, beta 2 (Adrb2)	-	10	-	-	-	-
NM_009627	Adrenomedullin (Adm)	-	5.5	-	2.1	-	-
BB532535	Advanced glycosylation end product-specific receptor (Ager)	-	27.4	-	-	-	-
BG064927	AE binding protein 2 (Aebp2)	-	2	-	-	-	-
BC026681	Afamin (Afm)	-	-2.3	-	-	-	-
BE570050	AHNAK nucleoprotein (desmoyokin) (Ahnak)	-	7.4	-	-	-	-
NM_016702	Alanine-glyoxylate aminotransferase (Agxt)	-	-2.4	-	-	-	-
AK005060	Alanine-glyoxylate aminotransferase 2-like 1 (Agxt2l1)	-	-12.2	-	-	-	-
BC020001	Aldehyde dehydrogenase 1 family, member B1 (Aldh1b1)	-	-3.8	-	-	-	-
BB251523	Aldehyde dehydrogenase 18 family, member A1 (Aldh18a1)	-	3.7	-	-	-	-
NM_007436	Aldehyde dehydrogenase family 3, subfamily A1 (Aldh3a1)	-	-	3.2	-	-	-
BQ175320	Aldhehyde dehydrogenase family 5, subfamily A1 (Aldh5a1)	-	-3	-	-	-	-
AV021656	Aldo-keto reductase family 1, member B7 (Akr1b7)	2.1	-	-	-	-	-
NM_054080	Aldo-keto reductase family 1, member C20 (Akr1c20)	-	-3.5	-	-	-	-
BC018333	Aldo-keto reductase family 1, member D1 (Akr1d1)	-	-5	-	-	-	-
NM_025337	Aldo-keto reductase family 7, member A5 (aflatoxin aldehyde reductase) (Akr7a5)	-	-2	-	-	-	-
BG065457	Aldolase 1, A isoform (Aldoa)	-	5.8	-	-	-	-
BI903628	AlkB, alkylation repair homolog 7 (Alkbh7)	-	-2.2	-	-	-	-
BB185854	alpha-2-macroglobulin /// hypothetical protein LOC677369	-	21.5	-	-	-	-
NM_008537	Alpha-methylacyl-CoA racemase (Amacr)	-	-2.4	-	-	-	-
NM_013792	Alpha-N-acetylglucosaminidase (Sanfilippo disease IIIB) (Naglu)	-	-2.2	-	-	-	-

BB797326	Amidohydrolase domain containing 1 (Amdhd1)	-2.7	-	-	-	-	-
NM_025371	Aminoacylase 1 (Acy1)	-	-2.7	-	-	-	-
BC018236	Aminolevulinate, delta-, dehydratase (Alad)	-	-2.4	-	-	-	-
BC022110	Aminolevulinic acid synthase 1 (Alas1)	3.4	-	-	-	-	-
D85596	AMP deaminase 3 (Ampd3)	-	6.4	-	-	-	-
NM_007446	Amylase 1, salivary (Amy1)	-	-	-	-	3.3	-
BB006088	Amylo-1,6-glucosidase, 4-alpha-glucanotransferase (Agl)	-	-2	-	-	-	-
BC005490	Amyloid beta (A4) precursor protein (App)	-	2	-	-	-	-
AB053307	Amyotrophic lateral sclerosis 2 (juvenile) homolog (Als2)	-	-2	-	-	-	-
BF322051	Anaphase promoting complex subunit 10 (Anapc10)	-	2	-	-	-	-
BC019491	Angiopoietin-like 3 (Angptl3)	-	-2.4	-	-	-	-
NM_020581	Angiopoietin-like 4 (Angptl4)	-	-	-	3.9	-	-
BC025904	Angiopoietin-like 6 (Angptl6)	-	-2.1	-	-	-	-
M55333	Angiotensin I converting enzyme (peptidyl-dipeptidase A) 1 (Ace)	-	4.2	-	-	-	-
BB483357	Angiotensin receptor-like 1 (Agtrl1)	-	-	-	-2.4	-	-
BB621938	Ankyrin repeat and BTB (POZ) domain containing 2 (Abtb2)	-	-3	-	-	-	-
AK003441	Ankyrin repeat and KH domain containing 1 (Ankhd1)	-	3	-	-	-	-
AK009959	Ankyrin repeat domain 1 (cardiac muscle) (Ankrd1)	-	3	-	-	-	-
NM_133971	Ankyrin repeat domain 10 (Ankrd10)	-	-	-	-	2.2	-
BQ032773	Ankyrin repeat domain 15 (Ankrd15)	-	-2.3	-	-	-	-
AK017778	Ankyrin repeat domain 24 (Ankrd24)	-	-2.1	-	-	-	-
AV256780	Ankyrin repeat domain 44 (Ankrd44)	-	2.3	-	-	-	-
BM119297	Ankyrin repeat domain 57 (Ankrd57)	-	3.5	-	-	-	-
NM_010730	Annexin A1 (Anxa1)	-	5.5	-	-	-	-
NM_007585	Annexin A2 (Anxa2)	-	3.6	-	-	-	-
AW702161	Annexin A3 (Anxa3)	-	6.6	-	-	-	-
D63423	Annexin A5 (Anxa5)	-	2.3	-	-	-	-
AF378762	Anthrax toxin receptor 1 (Antxr1)	-	3.1	-	-	-	-
AK018646	Apolipoprotein L3 (Apol3)	-	-6.3	-	-	-	-
BB449248	Apoptosis inhibitor 5 (Api5)	-	2.8	-	-	-	-
BB229969	Aquaporin 11 (Aqp11)	-	-3	-	-	-	-
BC024105	Aquaporin 9 (Aqp9)	-	-2.2	-	-	-	-
BC026209	Arachidonate 5-lipoxygenase activating protein (Alox5ap)	-	2.6	-	-	-	-
AK014338	Arginine-rich, mutated in early stage tumors (Armet)	-	2	-	-	-	-
AK004598	Armadillo repeat containing, X-linked 3 (Armcx3)	-	2.6	-	-	-	-
BE865094	Armadillo repeat containing, X-linked 4 (Armcx4)	-	2.6	-	-	-	-
AW542672	Arrestin domain containing 2 (Arrdc2)	-	-	-	2	-	-
BG072824	Arrestin domain containing 3 (Arrdc3)	-3	-	-	-	-	-

BC011080	Aryl hydrocarbon receptor nuclear translocator-like (Arntl)	-2.1	-	-	-	-	-
AK004030	Arylformamidase (Afmid)	-	-3.7	-	-	-	-
AV212753	Asparagine synthetase (Asns)	-	5.1	-	-	-	-
AK013880	Asparaginyl-tRNA synthetase (Nars)	-	2	-	-	-	-
NM_027857	Aspartoacylase (aminoacylase) 3 (Acy3)	-	-4.6	-	-	-	-
BC027152	AT rich interactive domain 5A (Mrf1 like) (Arid5a)	-	2.7	-	-	-	-
BB303874	ATPase, aminophospholipid transporter (APLT), class I, type 8A, member 1 (Atp8a1)	-	4	-	-	-	-
BB016769	ATPase, Class V, type 10D (Atp10d)	-	3.5	-	-	-	-
AV378604	ATPase, class VI, type 11A (Atp11a)	-	8.2	-	-	-	-
BM237637	Atpase, class VI, type 11C (Atp11c)	-	-	-	-	2.1	-
BG072708	ATPase, Cu++ transporting, alpha polypeptide (Atp7a)	-	2.5	-	-	-	-
BB144704	ATP-binding cassette, sub-family A (ABC1), member 1 (Abca1)	-	2.2	-	-	-	-
BC026496	ATP-binding cassette, sub-family A (ABC1), member 8a (Abca8)	-	-4.8	-	-	-	-
NM_008830	ATP-binding cassette, sub-family B (MDR/TAP), member 4 (Abcb4)	-	-2	-	-	-	-
NM_023732	ATP-binding cassette, sub-family B (MDR/TAP), member 6 (Abcb6)	-	-2.9	-	-	-	-
NM_018795	ATP-binding cassette, sub-family C (CFTR/MRP), member 6 (Abcc6)	-	-4.3	-	-	-	-
AW456685	ATP-binding cassette, sub-family D (ALD), member 2 (Abcd2)	-	-2.6	-	-	-	-
NM_031884	ATP-binding cassette, sub-family G (WHITE), member 5 (Abcg5)	-	-4.2	-	-	-	-
AF324495	ATP-binding cassette, sub-family G (WHITE), member 8 (Abcg8)	-	-2.6	-	-	-	-
AV323441	Avian musculoaponeurotic fibrosarcoma (v-maf) AS42 oncogene homolog (Maf)	-	-	-	-	2.1	-
NM_009046	Avian reticuloendotheliosis viral (v-rel) oncogene related B (Relb)	-	3.7	-	-	-	-
BG070296	AXIN1 up-regulated 1 (Axud1)	-	2	-	3.2	-	-
NM_007465	Baculoviral IAP repeat-containing 2 (Birc2)	-	2.7	-	-	-	-
NM_007464	Baculoviral IAP repeat-containing 3 (Birc3)	-	2.4	-	-	-	-
BC004826	Basal cell adhesion molecule (Bcam)	-	3.2	-	-	-	-
BC011486	Basic helix-loop-helix domain containing, class B, 8 (Bhlhb8)	-	3.2	-	-	-	-
AK016990	Basic leucine zipper transcription factor, ATF-like 2 (Batf2)	-	2.7	-	4.5	-	-
BI697337	Basic, immunoglobulin-like variable motif containing (Bivm)	-	-2	-	-	-	-
AF100339	B-cell leukemia/lymphoma 10 (Bcl10)	-	2	-	-	-	-
L16462	B-cell leukemia/lymphoma 2 related protein A1a (Bcl2a1a)	-	5.4	-	-	-	-
NM_033601	B-cell leukemia/lymphoma 3 (Bcl3)	-	3.1	-	3.3	-	-

NM_007570	B-cell translocation gene 2, anti-proliferative (Btg2)	-	3.5	-	-	-	-
NM_009770	B-cell translocation gene 3 (Btg3)	-	6.4	-	-	-	-
AW489168	Bcl-2 binding component 3 (Bbc3)	-	-2.7	-	-	-	-
BB212341	Bcl2 modifying factor (Bmf)	-2.4	-	-	-	-	-
NM_007546	Bcl2-interacting kille (Bik)	-2.1	-3.2	-	-	-	-
BG075800	Beta galactoside alpha 2,6 sialyltransferase 1 (St3gal1)	-	2.3	-	-	-	-
NM_133217	Beta-carotene 9', 10'-dioxygenase 2 (Bcdo2)	-	-2.7	-	-	-	-
BM217996	Bicaudal C homolog 1 (Bicc1)	-	2.3	-	-	-	-
AK014221	BMP-binding endothelial regulator (Bmper)	-	3.4	-	-	-	-
AI647987	Bone marrow stromal cell antigen 1 (Bst1)	-	3.6	-	-	-	-
AW546137	Bone morphogenic protein receptor, type II (serine/threonine kinase) (Bmpr2)	-	2.5	-	-	-	-
AK011545	Brain abundant, membrane attached signal protein 1 (Basp1)	-	5	-	-	-	-
NM_009052	Brain expressed gene 1 (Bex1)	-	2	-	-	-	-
AF390178	Brain-specific angiogenesis inhibitor 1-associated protein 2 (Baiap2)	-	-2.1	-	-	-	-
BB363154	Bri3 binding protein (Bri3bp)	-	-2	-	-	-	-
AV357135	Bromodomain adjacent to zinc finger domain 1A (Baz1a)	-	3.2	-	-	-	-
BB380312	Bromodomain PHD finger transcription factor (Bptf)	-	-	-	-	2.5	-
AV324314	BTB and CNC homology 1 (Bach1)	-	2.2	-	-	-	-
NM_009866	Cadherin 11 (Cdh11)	-	5.6	-	-	-	-
NM_009868	Cadherin 5 (Cdh5)	-	3.6	-	-	-	-
AF209905	Calcitonin receptor-like (Calcr1)	-	3	-	-	-	-
BG071931	Calcium/calmodulin-dependent protein kinase ID (Camk1d)	-2.2	-2.8	-	-	-	-
BG862223	Calcium/calmodulin-dependent protein kinase II, beta (Camk2b)	-	8.8	-	3.5	-	-
BI663014	Calponin 2 (Cnn2)	-	2	-	-	-	-
NM_023160	Camello-like 1 (Cml1)	-	-5.2	-	-	-	-
BB230757	cAMP responsive element binding protein 3-like 2 (Creb3l2)	-	2	-	-	-	-
AK007209	Capping protein (actin filament) muscle Z-line, beta (Capzb)	-	2.1	-	-	-	-
NM_007599	Capping protein (actin filament), gelsolin-like (Capg)	-	2.5	-	-	-	-
NM_011797	Carbonic anhydrase 14 (Car14)	-	-3.3	-	-	-	-
BB213876	Carbonic anhydrase 3 (Car3)	-	-2.3	-	-	-	-
NM_007607	Carbonic anhydrase 4 (Car4)	-	7.1	-	-	-	-
NM_007608	Carbonic anhydrase 5a, mitochondrial (Car5a)	-	-4.1	-	-	-	-
BC010758	Carbonyl reductase 2 (Cbr2)	-	30.9	-	-	-	-
BC022148	Carboxylesterase 5 (Ces5)	-	-2.3	-	-	-	-
AW550842	Carboxypeptidase D (Cpd)	-	2	-	-	-	-
BC010197	Carboxypeptidase E (Cpe)	-	2.7	-	-	-	-
AK017670	Carboxypeptidase M (Cpm)	-	3.9	-	-	-	-
BF020441	Caseinolytic protease X (Clpx)	-2.4	-	-	-	-	-

BC008152	Caspase 1 (Casp1)	-	2	-	-	-	-
NM_009808	Caspase 12 (Casp12)	-	3.2	-	-	-	-
NM_007609	Caspase 4, apoptosis-related cysteine peptidase (Casp4)	-	7	-	-	-	-
NM_011997	Caspase 8 associated protein 2 (Casp8ap2)	-	2	-	-	-	-
BM247599	Catalase (Cat)	-	-2.7	-	-	-	-
NM_021281	Cathepsin S (Ctss)	-	2.1	-	-	-	-
NM_007674	Caudal type homeo box 4 (Cdx4)	-	-2.3	-	-	-	-
NM_016900	Caveolin 2 (Cav2)	-	6.1	-	-	-	-
AB029929	Caveolin, caveolae protein 1 (Cav1)	-	10.4	-	-	-	-
AB012278	CCAAT/enhancer binding protein (C/EBP), beta (Cebpb)	-	4.4	-	-	-	-
BB831146	CCAAT/enhancer binding protein (C/EBP), delta (Cebpd)	-	18.1	-	9.2	-	-
AF199491	CCR4 carbon catabolite repression 4-like (Ccrn4l)	14	3.9	-	-	-	-
NM_009841	CD14 antigen (Cd14)	-	30.9	-	4.3	-	2.3
BC027283	CD177 antigen (Cd177)	-	2.5	-	5.5	-	-
AF004023	Cd200 antigen (Cd200)	-	5.7	-	-	-	-
BB560574	CD24a antigen (Cd24a)	-	12.8	-	-	-	-
NM_021893	CD274 antigen (Cd274)	-	7.9	-	6.9	-	-
NM_133654	CD34 antigen (Cd34)	-	3.7	-	-	-	-
AI385482	CD40 antigen (Cd40)	-	2.6	-	-	-	-
AW146109	CD44 antigen (Cd44)	-	5.4	-	-	-	-
NM_013706	CD52 antigen (Cd52)	-	2.4	-	-	-	-
NM_007651	CD53 antigen (Cd53)	-	3.1	-	-	-	-
NM_010016	CD55 antigen (Cd55)	-	2.2	-	-	-	-
AK017979	CD69 antigen (Cd69)	-	2.7	-	-	2.5	-
BC003476	CD74 antigen (invariant polypeptide of major histocompatibility complex, class II antigen-associated) (Cd74)	-	2.6	-	-	-	-
NM_009856	CD83 antigen (Cd83)	-	3.1	-	-	-	-
NM_007657	CD9 antigen (Cd9)	-	2.8	-	-	-	-
BB039247	CD93 antigen (Cd93)	-	4.4	-	-	-	-
BB224362	CDC14 cell division cycle 14 homolog B (Cdc14b)	-	-2.1	-	-	-	-
NM_025415	CDC28 protein kinase regulatory subunit 2 (Cks2)	-	-2.8	-	-	-	-
AV081797	cDNA (similar to apolipoprotein L, 3, predicted)	-	-	-	-	2	-
NM_009894	Cell death-inducing DNA fragmentation factor, alpha subunit-like effector B (Cideb)	-	-2.1	-	-	-	-
BE688580	Centaurin, gamma 2 (Centg2)	-	3.2	-	-	-	-
BB667247	Centrosomal protein 170 (Cep170)	-	2.5	-	-	-	-
NM_007672	Cerebellar degeneration-related 2 (Cdr2)	-	2.7	-	-	-	-
BB009037	Ceruloplasmin (Cp)	-	6.6	-	-	-	4.7
NM_010216	c-fos induced growth factor (figf)	-	2.7	-	-	-	-
BC025169	ChaC, cation transport regulator-like 1 (Chac1)	-	2.5	-	-	-	-

AK014605	Chaperone, ABC1 activity of bc1 complex like (Cabc1)	-2.4	-5.4	-	-	-	-
U50712	Chemokine (C-C motif) ligand 12 (Ccl12)	-	2.7	-	3.3	-	3.8
NM_011888	Chemokine (C-C motif) ligand 19 (Ccl19)	-	3.4	-	2	-	-
AF065933	Chemokine (C-C motif) ligand 2 (Ccl2)	-	9.2	-	3.4	-	-
AF099052	Chemokine (C-C motif) ligand 20 (Ccl20)	-	-	2.4	-	-	-
NM_011337	Chemokine (C-C motif) ligand 3 (Ccl3)	-	6.5	-	4.1	-	-
AF128218	Chemokine (C-C motif) ligand 4 (Ccl4)	-	2.2	-	-	-	-
BC002073	Chemokine (C-C motif) ligand 6 (Ccl6)	-	4.2	-	-	-	-
AF128196	Chemokine (C-C motif) ligand 9 (Ccl9)	-	-	2.8	-	-	-
AV231648	Chemokine (C-C motif) receptor 1 (Ccr1)	-	6.3	-	2.9	-	-
AJ318863	Chemokine (C-C motif) receptor-like 2 (Ccrl2)	-	5.3	-	2.6	-	-
NM_008176	Chemokine (C-X-C motif) ligand 1 (Cxcl1)	-	23.1	6.4	17.5	-	14.6
BB554288	Chemokine (C-X-C motif) ligand 1 (Cxcl1)	-	7	-	3.6	-	-
NM_021274	Chemokine (C-X-C motif) ligand 10 (Cxcl10)	-	30.5	-	20	-	6
NM_019494	Chemokine (C-X-C motif) ligand 11 (Cxcl11)	-	2	-	10.9	-	-
AF030636	Chemokine (C-X-C motif) ligand 13 (Cxcl13)	-	17.1	-	-	-	2.3
AF252873	Chemokine (C-X-C motif) ligand 14 (Cxcl14)	-	2.7	-	-	-	-
NM_011339	Chemokine (C-X-C motif) ligand 15 (Cxcl15)	-	60.9	-	-	-	-
BC019961	Chemokine (C-X-C motif) ligand 16 (Cxcl16)	-	2	-	-	-	-
NM_009140	Chemokine (C-X-C motif) ligand 2 (Cxcl2)	-	53.7	7.7	18.2	-	-
NM_009141	Chemokine (C-X-C motif) ligand 5 (Cxcl5)	-	8.8	14.6	9.3	-	5.3
NM_008599	Chemokine (C-X-C motif) ligand 9 (Cxcl9)	-	11.8	-	11.8	-	-
D87747	Chemokine (C-X-C motif) receptor 4(Cxcr4)	-	-	-	-	2.2	-
BC015254	Chemokine (C-X-C motif) receptor 7 (Cxcl7)	-	7.6	-	-	-	-
AW228687	Chemokine-like receptor 1 (Cmklr1)	-	2.3	-	-	-	-
BC005611	Chitinase 3-like 1 (Chi3l1)	-	11.8	-	-	-	-
NM_009892	Chitinase 3-like 3 (Chi3l3)	-	51.8	-	-	-	-
BC011134	Chitinase, acidic (Chia)	-	4.1	-	-	-	-
AF108501	Chloride channel calcium activated 1 (Clca1)	-	2.7	-	5.2	-	-
AK009020	Chloride intracellular channel 3 (Clic3)	-	3.6	-	-	-	-
BB814844	Chloride intracellular channel 4 (mitochondrial) (Clic4)	-	2.8	-	-	-	-
BB236747	Chloride intracellular channel 5 (Clic5)	-	12.1	-	-	-	-
BQ176424	Chloride intracellular channel 6 (Clic6)	-	2	-	-	-	-
BC020534	Cholecystokinin A receptor (Cckar)	-	4.1	-	-	-	-
NM_009890	Cholesterol 25-hydroxylase (Ch25h)	-	5.4	2.7	4	-	-
BC023112	Chondroitin sulfate GalNAcT-2 (Galnact2)	-	2.5	-	-	-	-
BI693188	Chromobox homolog 2 (Cbx2)	-	-2	-	-	-	-
AF042158	Class II transactivator (C2ta)	-	-	-	2.3	-	-
BC021770	Claudin 10 (Cldn10)	-	2	-	-	-	-
NM_013805	Claudin 5 (Cldn5)	-	2.6	-	-	-	-
BC008104	Claudin 7 (Cldn7)	-	3	-	-	-	-
NM_019815	Claudin 8 (Cldn8)	-	8.9	-	-	-	-
AF349453	Claudin 8 (Cldn8)	-	8.1	-	-	-	-
BB736632	CLP1, cleavage and polyadenylation factor I subunit, homolog (Clp1)	-	2.7	-	-	-	-
BQ173958	Coagulation factor II (thrombin) receptor (F2r)	-	2.9	-	-	-	-
BC024886	Coagulation factor III (F3)	-	2.8	-	-	-	-

NM_007972	Coagulation factor X (F10)	-	-	-	2.5	-	-
NM_028784	Coagulation factor XIII, A1 subunit (F13a1)	-	4.7	-	-	-	-
BE653037	Coatomer protein complex, subunit gamma 2, antisense 2 (Copg2as2)	-	-	-	-2.5	-	-
AK006551	Coenzyme Q10 homolog B (Coq10b)	2	-	-	-	-	-
AV108872	Coiled-coil domain containing 117 (Ccdc117)	-	2.5	-	-	-	-
AW060797	Coiled-coil domain containing 85A (Ccdc85a)	-	3.5	-	-	-	-
BM941868	Colony stimulating factor 2 receptor, alpha, low-affinity (granulocyte-macrophage) (Csf2ra)	-	2.8	-	-	-	-
BB769628	Colony stimulating factor 2 receptor, beta 1, low-affinity (granulocyte-macrophage) (Csf2rb1)	-	5	-	-	-	2.4
NM_007781	Colony stimulating factor 2 receptor, beta 2, low-affinity (granulocyte-macrophage) (Csf2rb2)	-	3.5	-	-	-	-
NM_009971	Colony stimulating factor 3 (granulocyte) (Csf3)	-	3.8	-	2.1	-	-
NM_009777	Complement component 1, q subcomponent, beta polypeptide (C1qb)	-	-	-	2.4	-	-
NM_008198	Complement factor B (Cfb)	-	-	-	2.5	-	-
NM_010217	Connective tissue growth factor (Ctgf)	3.3	7.3	-	-	-	-
AW548480	Copine VIII (Cpne8)	-	5.9	-	-	-	-
BC002136	Coronin, actin binding protein 1A (Coro1a)	-	3.5	-	-	-	-
AV016515	Crystallin, alpha B (Cryab)	-	3.9	-	-	-	-
NM_021352	Crystallin, beta B3 (Crybb3)	-	6.8	-	-	-	-
NM_030004	Crystallin, lambda 1 (Cryl1)	-	-3	-	-	-	-
BG072903	C-terminal binding protein 2 (Ctbp2)	-	2.8	-	-	-	-
BB231897	CTTNBP2 N-terminal like (Cttnbp2nl)	-	2.4	-	-	-	2
BB035924	C-type lectin domain family 1, member a (Clec1a)	-	2.7	-	-	-	-
NM_010819	C-type lectin domain family 4, member d (Clec4d)	-	3.9	-	3.6	-	-
NM_019948	C-type lectin domain family 4, member e (Clec4e)	-	2.5	-	2.3	-	3.8
AF240358	C-type lectin domain family 4, member n (Clec4n)	-	5	-	-	-	-
NM_020008	C-type lectin domain family 7, member a (Celc7a)	-	3.2	-	-	-	-
BB129488	Cut-like 2 (Cutl2)	-2.2	-	-	-	-	-
BB666837	CWF19-like 1, cell cycle control (Cwf19l1)	-	-2	-	-	-	-
AK015150	CXXC finger 5 (Cxxc5)	-	-2.1	-	-	-	-
BB357590	Cyclin D binding myb-like transcription factor 1 (Dmtf1)	-	-	-	-	2.7	-
BE685667	Cyclin D3 (Ccnd3)	-	-	-	-	3.9	-
AK007630	Cyclin-dependent kinase inhibitor 1A (P21) (Cdkn1a)	-	8.2	2.3	5.2	-	-
BC026595	Cystathionine beta-synthase (Cbs)	-2.3	-	-	-	-	-
BF124540	Cysteine and glycine-rich protein 1 (Csrp1)	-	2.6	-	-	-	-
AK008165	Cysteine conjugate-beta lyase 1 (Ccbl1)	-	-2.1	-	-	-	-
BB533736	Cysteine rich protein 61 (Cyr61)	-	-	-3.8	-8	-	-

AK018666	Cysteine rich transmembrane BMP regulator 1 (chordin like) (Crim1)	-	2.1	-	-	-	-
BB558800	Cysteine-rich secretory protein LCCL domain containing 2 (Crispld2)	-	3.5	-	-	-	-
NM_016748	Cytidine 5'-triphosphate synthase (Ctps)	-	3.1	-	2.2	-	-
AK008793	Cytidine deaminase (Cda)	-	-2.8	-	-	-	-
AK018713	Cytochrome b-245, alpha polypeptide (Cyba)	-	3.2	-	-	-	-
AV373944	Cytochrome b-245, beta polypeptide (Cybb)	-	3.9	-	-	-	-
BC006732	Cytochrome b-561 (Cyb561)	-	10.4	-	-	-	-
NM_009998	Cytochrome P450, 2b10, phenobarbitol inducible, type b (Cyp2b10)	3.1	-	-	-	-	-
NM_009992	Cytochrome P450, family 1, subfamily a, polypeptide 1 (Cyp1a1)	-	-	-	-2.3	-	-
BI251808	Cytochrome P450, family 1, subfamily b, polypeptide 1 (Cyp1b1)	-	-	-	-	-	2.2
NM_007809	Cytochrome P450, family 17, subfamily a, polypeptide 1 (Cyp17a1)	2.3	-	-	-	-	-
NM_007812	Cytochrome P450, family 2, subfamily a, polypeptide 4 (Cyp2a4)	-	-3.3	-	-	-	-
NM_007813	Cytochrome P450, family 2, subfamily b, polypeptide 13 (Cyp2b13)	-	-11.1	-	-	-	-
AF047725	Cytochrome P450, family 2, subfamily c, polypeptide 38 (Cyp2c38)	-	-2.3	-	-	-	-
BC025819	Cytochrome P450, family 2, subfamily c, polypeptide 44 (Cyp2c44)	-	-2.7	-	-	-	-
AI256046	Cytochrome P450, family 2, subfamily c, polypeptide 50 (Cyp2c50)	-	-2.9	-	-	-	-
NM_028089	Cytochrome P450, family 2, subfamily c, polypeptide 55 (Cyp2c55)	2.9	-	-	-	-	-
BF683039	Cytochrome P450, family 2, subfamily d, polypeptide 22 (Cyp2d22)	-	-2.3	-	-	-	-
NM_013809	Cytochrome P450, family 2, subfamily g, polypeptide 1 (Cyp2g1)	-	-3.3	-	-	-	-
NM_010001	Cytochrome P450, family 2. subfamily c, polypeptide 37 (Cyp2c37)	-	-3.2	-	-	-	-
NM_007823	Cytochrome P450, family 4, subfamily b, polypeptide 1 (Cyp4b1)	-	3.2	-	-	-	-
BC011228	Cytochrome P450, family 4, subfamily f, polypeptide 14 (Cyp4f14)	-	-4.5	-	-	-	-
NM_134127	Cytochrome P450, family 4, subfamily f, polypeptide 15 (Cyp4f15)	-	-5.1	-	-	-	-
NM_007825	Cytochrome P450, family 7, subfamily b, polypeptide 1 (Cyp7b1)	-	-	2.8	-	-	-
BC010973	Cytochrome P450, family 8, subfamily b, polypeptide 1 (Cyp8b1)	-	-7.6	-	-	-	-
NM_009895	Cytokine inducible SH2-containing protein (Cish)	-	2.5	-	-	-	-
NM_007796	Cytotoxic T lymphocyte-associated protein 2 alpha (Ctla2a)	-	5.1	-	-	-	-

BG064656	Cytotoxic T lymphocyte-associated protein 2 beta (Ctla2b)	-	4.5	-	-	-	-
BB550183	D site albumin promoter binding protein (Dbp)	-	-	-4.8	-	-	-
AV349520	D-2-hydroxyglutarate dehydrogenase (D2hgdh)	-	-2.3	-	-	-	-
AV214253	DEAD (Asp-Glu-Ala-Asp) box polypeptide 39 (Ddx39)	-	2.1	-	-	-	-
BF715043	Dedicator of cytokinesis 10 (Protein zizimin 3) (Dock10)	-	2.5	-	-	-	-
BB045510	Dedicator of cytokinesis 4 (Dock4)	-	-	-	-2.2	-	-
NM_028785	Dedicator of cytokinesis 8 (Dock8)	-	-2.9	-	-	-	-
NM_011303	Dehydrogenase/reductase (SDR family) member 3 (Dhrs3)	-	-2.1	-	-	-	-
AK009385	Dehydrogenase/reductase (SDR family) member 7 (Dhrs7)	-	-2.1	-	-	-	-
BB705351	Deltex 3-like (Dtx3l)	-	-	-	2.3	-	-
NM_019957	Deoxyribonuclease II beta (Dnase2b)	-	-2.2	-	-	-	-
AK014624	DEP domain containing 6 (Depdc6)	-	-2.2	-	-	-	-
BI452905	Dermokine (Dmkn)	-	3.8	-	-	-	-
AB072269	Desmoglein 2 (Dsg2)	-	-2.5	-	-	-	-
AV297961	Desmoplakin (Dsp)	-	-2.8	-	-	-	-
AV077160	Development and differentiation enhancing factor 2 (Ddef2)	-2.1	-	-	-	-	-
NM_021428	Dexamethasone-induced transcript (Dexi)	-	-2.4	-	-	-	-
BC012247	Dicarbonyl L-xylulose reductase (Dcxr)	-	-2.3	-	-	-	-
AF021031	DiGeorge syndrome critical region gene 6 (Dgcr6)	-	-2.6	-	-	-	-
AK005050	Dihydrodiol dehydrogenase (dimeric) (Dhdh)	-	-3.4	-	-	-	-
AW490997	Dihydropyrimidinase (Dpys)	-	-4.1	-	-	-	-
BQ174209	Dihydropyrimidinase-like 2 (Dpysl2)	-	2.4	-	-	-	-
BB770857	Dimethylarginine dimethylaminohydrolase 1 (Ddah1)	-	-2.6	-	-	-	-
AI647687	Dipeptidase 1 (renal) (Dpep1)	-	3.4	-	-	-	-
BB667346	Dipeptidylpeptidase 9 (Dpp9)	-	2.1	-	-	-	-
BB234940	Discoidin domain receptor family, member 1 (Ddr1)	-	8.6	-	-	-	-
AF241240	DNA cross-link repair 1A, PSO2 homolog (Dclre1a)	2.5	-	-	-	-	-
AK017926	DNA-damage-inducible transcript 4 (Ddit4)	-	3.1	-	3.6	-	-
BM118858	DnaJ (Hsp40) homolog, subfamily B, member 6 (Dnajb6)	-	3.3	-	-	-	-
NM_013760	DnaJ (Hsp40) homolog, subfamily B, member 9 (Dnajb9)	-	2	-	-	-	-
NM_013888	DnaJ (Hsp40) homolog, subfamily C, member 12 (Dnajc12)	-	3.5	-	-	-	-
AF071068	Dopa decarboxylase (Ddc)	-	-6.8	-	-	-	-
BM117900	Double C2, beta (Doc2b)	-	-	-	2.9	-	-
BB326709	Doublecortin and CaM kinase-like 3 (Dcamkl3)	-	-2.4	-	-	-	-
BQ174742	Downstream neighbor of SON (Donson)	-	2.2	-	-	-	-

NM_013642	Dual specificity phosphatase 1 (Dusp1)	-	-	-	-	-	2.1
AK010883	Duffy blood group, chemokine receptor (Darc)	-	-	-	2	-	-
NM_011722	Dynactin 6 (Dctn6)	-	-	-	-	3.1	-
NM_133796	Dynein light chain LC8-type 2 (Dynll2)	-	-2.3	-	-	-	-
BB723867	Dysferlin (Dysf)	-	-	-	-	2.1	-
BB151715	E26 avian leukemia oncogene 1, 5' domain (Ets1)	-	2	-	-	-	-
BC005486	E26 avian leukemia oncogene 2, 3' domain (Ets2)	-	4	-	-	-	-
NM_007921	E74-like factor 3 (Eif3)	-	2.1	-	-	-	-
NM_007913	Early growth response 1 (Egr1)	-5.2	-	-	-	-	-
BM120053	Ectodermal-neural cortex 1 (Enc1)	-	2.3	-	-	-	2.2
BI151440	Ectonucleoside triphosphate diphosphohydrolase 1 (Entpd1)	-	2.4	-	-	-	-
AV381133	Ectonucleoside triphosphate diphosphohydrolase 7 (Entpd7)	-	2.3	-	-	-	-
AK008590	Ectonucleoside triphosphate diphosphohydrolase 8 (Entpd8)	-	-2.3	-	-	-	-
AV280361	Ectonucleotide pyrophosphatase/phosphodiesterase 4 (Enpp4)	-	2.4	-	-	-	-
NM_019397	EGF-like-domain, multiple 6 (Egfl6)	-	4.3	-	-	-	-
BC022961	EGL nine homolog 3 (Egln3)	-	-2.4	-	-	-	-
AV222756	EH-domain containing 2 /// similar to EH-domain containing 2 (Ehd2)	-	2.2	-	-	-	-
NM_133838	EH-domain containing 4 (Ehd4)	-	3.7	-	-	-	-
BC011218	Elastase 1, pancreatic (Ela1)	-	-3.9	-	-	-	-
BB811788	Elastin microfibril interfacer 2 (Emilin2)	-	2.7	-	-	-	-
NM_021292	Ellis van Creveld gene homolog (Evc)	-	-2.5	-	-	-	-
NM_019423	Elongation of very long chain fatty acids (FEN1/Elo2, SUR4/Elo3, yeast)-like 2 (Elovl2)	-	-2.2	-	-	-	-
BG064842	Embigin (Emb)	-	3.2	-	-	-	-
BQ044016	Enabled homolog (Enah)	-	3.2	-	-	-	-
AK013765	Endothelial cell growth factor 1 (platelet-derived) (Ecgf1)	-2.5	-2.6	-	-	-	-
AF361882	Endothelial cell-specific adhesion molecule (Esam1)	-	2.3	-	-	-	-
BC020038	Endothelial cell-specific molecule 1 (Esm1)	-	-	-	-3.3	-	-
U81983	Endothelial PAS domain protein 1 (Epas1)	-	2.8	-	-	-	-
BC008277	Endothelin receptor type A (Ednra)	-	2.4	-	-	-	-
NM_023737	Enoyl-Coenzyme A, hydratase/3-hydroxyacyl Coenzyme A dehydrogenase (Ehhadh)	-	-2.6	-	-	-	-
AF306665	Eosinophil-associated, ribonuclease A family, member 2 (Ear2)	-	17.3	-	-	-	-
NM_017388	Eosinophil-associated, ribonuclease A family, member 3 (Ear3)	-	25.6	-	-	-	-
D38146	Ephrin A1 (Efna1)	-2.1	-	-	-	-	-
U03425	Epidermal growth factor receptor (Egfr)	-	5.1	-	-	-	-
U25633	Epithelial membrane protein 1 (Emp1)	-	7.9	-	-	-	-

BE571790	Epithelial membrane protein 2 (Emp2)	-	4.3	-	-	-	-
BC015076	Epithelial V-like antigen 1 (Eva1)	-	-2.1	-	-	-	-
NM_133753	ERBB receptor feedback inhibitor 1 (Errfi1)	-	2.8	-	2.2	3.9	5.6
BM234652	ERO1-like (Ero1l)	-	2.4	-	-	-	-
NM_133660	Esterase 22 (Es22)	-	-2.6	-	-	-	-
BC010592	Ethylmalonic encephalopathy 1 (Ethe1)	-	-5.1	-	-	-	-
BC006789	Ets homologous factor (Ehf)	-	2.2	-	-	-	-
BI456953	Ets variant gene 3 (Etv3)	-	2.1	-	-	-	-
BM200591	Eukaryotic translation initiation factor 1A (Eif1a)	-	2.3	-	-	-	-
AV124537	Extra cellular link domain-containing 1 (Xlkd1)	-	3.8	-	-	-	-
BG070740	Far upstream element (FUSE) binding protein 1 (Fubp1)	-	2	-	-	-	-
NM_007987	Fas (TNF receptor superfamily member) (Fas)	-	2.1	-	2	-	-
BB189927	Fatty acid amide hydrolase (Faah)	-	-2.6	-	-	-	-
BC002148	Fatty acid binding protein 4, adipocyte (Fabp4)	-	2.1	-	-	-	-
NM_019699	Fatty acid desaturase 2 (Fadds2)	-	-3	-	-	-	-
AV026617	FBJ osteosarcoma oncogene (Fos)	-	3.5	-	-	-	2.1
BG076338	F-box protein 21 (Fbxo21)	-2	-	-	-	-	-
AK006369	F-box protein 30 (Fbxo30)	-	2.1	-	-	-	-
NM_133765	F-box protein 31 (Fbxo31)	-	2.7	-	-	-	-
BC027310	Fc fragment of IgG, low affinity IIIa, receptor (Fcgr3a)	-	2.1	-	-	-	3.1
NM_010185	Fc receptor, IgE, high affinity I, gamma polypeptide (Fcer1g)	-	2.3	-	-	-	-
NM_010188	Fc receptor, IgG, low affinity III (Fcgr3)	-	2.1	-	-	-	-
NM_007993	Fibrillin 1 (Fbn1)	-	2.6	-	-	-	-
BC021946	Fibrinogen-like protein 1 (Fgl1)	-	2.6	-	-	-	-
BF136544	Fibrinogen-like protein 2 (Fgl2)	-	3.1	-	-	-	-
BM234360	Fibronectin 1 (Fn1)	-2	-	-	-	-	-
BG064539	Fibronectin type III domain containing 3B (Fndc3b)	-	2.6	-	-	-	-
AK013203	Fibronectin type III domain containing 4 (Fndc4)	-	-2	-	-	-	-
BG070068	Filamin binding LIM protein 1 (Fblim1)	-	2.3	-	-	-	-
NM_024169	FK506 binding protein 11 (Fkbp11)	-	3	-	-	-	-
BB027759	FK506 binding protein 14 (Fkbp14)	-	-	-	2.2	-	-
BB456860	FK506 binding protein 4 (Fkbp4)	-	-2.7	-	-	-	-
U16959	FK506 binding protein 5 (Fkbp5)	-	2.4	2	8.1	-	2.4
NM_008030	Flavin containing monooxygenase 3 (Fmo3)	-	-7.9	-	-	-	-
NM_008027	Flotillin 1 (Flot1)	-	2	-	-	-	-
BI452727	Follistatin-like 1 (Fstl1)	-	3	-	-	-	-
BB437522	Forkhead box F1a (Foxf1a)	-	2.4	-	-	-	-
BB364488	Forkhead box O3a (Foxo3a)	-	-	-	2	-	-
NM_080845	Formiminotransferase cyclodeaminase (Ftcd)	-	-2.1	-	-	-	-
AK017338	Formin-like 2 (Fmnl2)	-	2.9	-	-	-	-
NM_013521	Formyl peptide receptor 1 (Fpr1)	-	2.8	-	-	-	-

NM_008039	Formyl peptide receptor, related sequence 2 (Fpr-rs2)	-	8.4	-	-	-	-
BM245170	Fos-like antigen 2 /// similar to fos-like antigen 2 (Fosl2)	-	4.8	-	-	-	-
AV345166	Frizzled homolog 8 (Fzd8)	-	-2.7	-	-	-	-
NM_022014	Fructosamine 3 kinase (Fn3k)	-	-3.2	-	-	-	-
BC026949	Fumarylacetoacetate hydrolase domain containing 1 (Fahd1)	-	-2.7	-	-	-	-
BB527078	Furry homolog (Fry)	-	2.5	-	-	-	-
NM_030701	G protein-coupled receptor 109A (Gpr109a)	-	4	-	-	-	-
AW547876	G protein-coupled receptor 116 (Gpr116)	-	4	-	-	-	-
BB726971	G protein-coupled receptor 137B (Gpr137b)	-	2.7	-	-	-	-
BC018381	G protein-coupled receptor 177 (Gpr177)	-	2.6	-	-	-	-
BC019379	G protein-coupled receptor kinase 5 (Gprk5)	-	2.4	-	-	-	-
BG064659	G protein-coupled receptor, family C, group 5, member A (Gprc5a)	-	4.8	-	-	-	-
BC008228	G protein-coupled receptor, family C, group 5, member C (Gprc5c)	-	-2.3	-	-	-	-
NM_008059	G0/G1 switch gene 2 (G0s2)	-	-3.4	-	-	-	-
AV307219	Galactose mutarotase (Galm)	-	-3.2	-	-	-	-
AF180518	Gamma-aminobutyric acid (GABA(A)) receptor-associated protein-like 1 (Gabarapl1)	-	-2.5	-	-	-	-
NM_010268	Ganglioside-induced differentiation-associated-protein 10 (Gdap10)	-	4.3	-	2.5	-	-
M63801	Gap junction membrane channel protein alpha 1 (Gja1)	-	7.2	-	-	-	-
AF216832	Gap junction membrane channel protein alpha 4 (Gja4)	-	2.7	-	-	-	-
AV224521	Gelsolin (Gsn)	-	24	-	-	-	-
BB829808	Gene model 1960 (Gm1960)	-	28.9	35.3	15.9	-	-
BB053232	Gene model 944, (Gm944)	-	2.2	-	-	-	-
BG075741	General transcription factor II A, 2 (Gtf2a2)	-	2.5	-	-	-	-
BC016637	General transcription factor IIB (Gtf2b)	-	2.1	-	-	-	-
AV251087	GLE1 RNA export mediator-like (Gle1l)	-	2.2	-	-	-	-
BC025083	GLI pathogenesis-related 1 (glioma) (Glipr1)	-	2.1	-	-	-	-
BM208214	GLI pathogenesis-related 2 (Glipr2)	-	2.3	-	-	-	-
NM_022024	Glia maturation factor, gamma (Gmfg)	-	2.1	-	-	-	-
BC011139	Glucokinase (Gck)	-	-2.4	-	-	-	-
BC012412	Glucokinase regulatory protein (Gckr)	-	-2.9	-	-	-	-
NM_011937	Glucosamine-6-phosphate deaminase 1 (Gnpda1)	-	-2.1	-	-	-	-
NM_008105	Glucosaminyl (N-acetyl) transferase 2, I-branching enzyme (Gcnt2)	-	2.7	-	-	-	-
NM_008061	Glucose-6-phosphatase, catalytic (G6pc)	-	-8.5	-	-	-	-
AA792094	Glutamate oxaloacetate transaminase 1, soluble (Got1)	-	-2.7	-	-	-	-
BC019374	Glutamate-cysteine ligase, catalytic subunit (Gclc)	-	-2.3	-	-	-	-
NM_008129	Glutamate-cysteine ligase, modifier subunit (Gclm)	-	-	2.2	-	-	-

AK008086	Glutamic pyruvic transaminase 1, soluble (Gpt1)	-	-2.9	-	-	-	-
BE373308	Glutaminase (Gls)	-	3.7	-	-	-	-
AI195532	Glutaminase 2 (liver, mitochondrial) (Gls2)	-	-2.9	-	-	-	-
AF334736	Glutamine fructose-6-phosphate transaminase 1 (Gfpt1)	-	2.1	-	-	-	-
NM_008161	Glutathione peroxidase 3 (Gpx3)	-	12.4	-	-	-	-
NM_010362	Glutathione S-transferase omega 1 (Gsto1)	-	-2.1	-	-	-	-
NM_008182	Glutathione S-transferase, alpha 2 (Yc2) (Gsta2)	-	-	3.6	-	-	-
AI172943	Glutathione S-transferase, alpha 3 (Gsta3)	-	-3.8	-	-	-	-
NM_010357	Glutathione S-transferase, alpha 4 (Gsta4)	-	-2.1	-	-	-	-
AF464943	Glutathione S-transferase, mu 4 (Gstm4)	-	-3.7	-	-	-	-
NM_026672	Glutathione S-transferase, mu 7 (Gstm7)	-	-2.4	-	-	-	-
NM_008149	Glycerol-3-phosphate acyltransferase, mitochondrial (Gpam)	-	-2.3	-	-	-	-
BC019391	Glycerol-3-phosphate dehydrogenase 1 (soluble) (Gpd1)	-	-3.1	-	-	-	-
BB727537	Glycerol-3-phosphate dehydrogenase 1-like (Gpd1l)	-	-2.4	-	-	-	-
BC021322	Glycogen synthase 2 (Gys2)	2	-3.2	-	-	-	-
NM_013755	Glycogenin Ggyg)	-	2.4	-	-	-	-
U05264	Glycoprotein 49 A (Gp49a); also called leukocyte immunoglobulin-like receptor, subfamily B, member 4 (Lilrb4)	-	5	-	-	-	2.9
AF297615	Glycoprotein galactosyltransferase alpha 1, 3 (Ggta1)	-	2.6	-	-	-	-
BF449735	Glycoprotein m6a (Gpm6a)	-	3	-	-	-	-
AK016567	Glycoprotein m6b (Gpm6b)	-	6.7	-	-	-	-
NM_008156	Glycosylphosphatidylinositol specific phospholipase D1 (Gpld1)	-	-2.6	-	-	-	-
BC004651	GM2 ganglioside activator protein (Gm2a)	-	-2	-	-	-	-
D78270	Golgi autoantigen, golgin subfamily a, 3 (Golga3)	-	2	-	-	-	-
BC021450	Grancalcin (Gca)	-	2	-	-	-	-
AI323528	Growth arrest and DNA-damage-inducible 45 beta (Gadd45b)	-	4.4	-	2.1	-	-
AK007410	Growth arrest and DNA-damage-inducible 45 gamma (Gadd45g)	-	8.3	-	-	-	-
BB550400	Growth arrest specific 1 (Gas1)	-	-3.9	-	-	-	-
BI650268	Growth arrest specific 5 (Gas5)	-	2.9	-	-	-	-
NM_019521	Growth arrest specific 6 (Gas6)	-	2.4	-	-	-	-
AW049055	Growth factor receptor bound protein 2-associated protein 2 (Gab2)	-	2.9	-	-	-	-
NM_010346	Growth factor receptor bound protein 7 (Grb7)	-	-3.2	-	-	-	-
NM_010284	Growth hormone receptor (Ghr)	-	-2.1	-	-	-	-
U10551	GTP binding protein (gene overexpressed in skeletal muscle) (Gem)	-	2.9	-	-	-	-
BC005577	GTPase, IMAP family member 4 (Gimap4)	-	2.1	-	-	-	-

BM243571	GTPase, very large interferon inducible 1 (Gvin1)	-	12.6	-	-	-	-
AF015887	Guanidinoacetate methyltransferase (Gamt)	-	-4	-	-	-	-
AV021455	Guanine nucleotide binding protein (G protein), gamma 2 subunit (Gng2)	-	2.2	-	-	-	-
BC022793	Guanine nucleotide binding protein, alpha inhibiting 1 (Gnai1)	-	2.2	-	-	-	-
BM199850	Guanine nucleotide binding protein-like 2 (nucleolar) (Gnl2)	-	2.4	-	-	-	-
BF021177	Guanine nucleotide binding protein-like 3 (nucleolar) (Gnl3)	-	2.2	-	-	-	-
BC010229	Guanylate binding protein 6 (Gbp6)	-	4	-	4.3	-	3
BE197524	Guanylate nucleotide binding protein 2 (Gbp2)	-	22.4	-	4.6	-	5.5
NM_018734	Guanylate nucleotide binding protein 3 (Gbp3)	-	11.1	-	4.2	-	2.7
BC019856	Gulonolactone (L-) oxidase (Gulo)	-	-2.8	-	-	-	-
AI326893	Hairy and enhancer of split 6 (Hes6)	-	-2.3	-	-	-	-
AF247040	Hairy and enhancer of split 6 (Hes6)	-	-2.3	-	-	-	-
BC003491	Haloacid dehalogenase-like hydrolase domain containing 3 (Hdhd3)	-	-3.1	-	-	-	-
U03561	Heat shock protein 1 (Hspb1)	-	3.6	-	-	-	-
AW763765	Heat shock protein 1A (Hspa1a)	-	-	2.9	-	-	-
M12573	Heat shock protein 1B (Hspa1b)	-	-	-	-	2.2	-
AK015214	Hect domain and RLD 5 (Herc5)	-	4.1	-	-	-	-
BI251153	Helicase with zinc finger domain (Helz)	-	-	-	-	2.1	-
NM_008225	Hematopoietic cell specific Lyn substrate 1 (Hcls1)	-	2.2	-	-	-	-
AK009239	HemK methyltransferase family member 1 (Hemk1)	-	-2.2	-	-	-	-
AK009636	Hemochromatosis type 2 (juvenile) (Hfe2)	-	-5.3	-	-	-	-
BC011246	Hemopexin (Hpxn)	-	-	5.4	2.3	-	-
NM_010407	Hemopoietic cell kinase (Hck)	-	4	-	-	-	-
AV378394	Hepatoma-derived growth factor, related protein 3 (Hdgfrp3)	-	2.4	-	-	-	-
BB713410	Hexokinase 1 (Hk1)	-	2.4	-	-	-	-
X67668	High mobility group box 2 (Hmgb2)	-	2	-	-	-	-
AW555814	Histidine acid phosphatase domain containing 1 (Hisppd1)	-	2.1	-	-	-	-
L07645	Histidine ammonia lyase (Hal)	-	-2.6	-	-	-	-
BG072171	Histidine decarboxylase (Hdc)	-	7.9	-	-	-	-
M15848	Histocompatibility 2, class II antigen A, beta 1 (H2-Ab1)	-	2.6	-	-	-	-
NM_010382	Histocompatibility 2, class II antigen E beta (H2-Eb1)	-	3.1	-	-	-	-
NM_010386	Histocompatibility 2, class II, locus Dma (H2-Dma)	-	2.5	-	-	-	-
NM_010388	Histocompatibility 2, class II, locus Mb1 (H2-DMb1)	-	2.2	-	-	-	-
M33151	Histocompatibility 2, D region locus 1 (H2-D1)	-	2.3	-	-	-	-

BC018402	Histocompatibility 2, K1, K region (H2-K1)	-	3	-	-	-	-
BC010602	Histocompatibility 2, Q region locus 1 (H2-Q1)	-	2.7	-	-	-	-
AV127319	Histone cluster 2, H2be (Hist2h2be)	-	-2.4	-	-	-	-
BB183559	Histone deacetylase 11 (Hdac11)	-	-2.5	-	-	-	-
BB130716	HIV-1 Rev binding protein (Hrb)	-	2.2	-	-	-	-
BB243375	HIV-1 Rev binding protein-like (Hrbl)	-	-2.2	-	-	-	-
BC024546	Homeobox only domain (Hod)	-	2.8	-	-	-	-
AB017136	Homer homolog 2 (Homer2)	-	-2.8	-	-	-	-
NM_022331	Homocysteine-inducible, endoplasmic reticulum stress-inducible, ubiquitin-like domain member 1 (Herpud1)	-	2.2	-	-	-	-
AK002584	Homogentisate 1, 2-dioxygenase (Hgd)	-	-4	-	-	-	-
BF580567	Host cell factor C1 regulator 1 (XPO1-dependent) (Hcfc1r1)	-	-2	-	-	-	-
NM_010437	Human immunodeficiency virus type I enhancer binding protein 2 (Hivep2)	-	2.9	-	-	-	-
BB164127	Human immunodeficiency virus type I enhancer binding protein 3 (Hivep3)	-	5.5	-	-	-	-
AF079222	Hyaluronan mediated motility receptor (RHAMM) (Hmmr)	-	-	-	-	-2.1	-
BC026757	Hydroxy-delta-5-steroid dehydrogenase, 3 beta- and steroid delta-isomerase 2 (Hsd3b2)	-	-4.9	-	-	-	-
M77015	Hydroxy-delta-5-steroid dehydrogenase, 3 beta- and steroid delta-isomerase 3 (Hsd3b3)	-	-3.9	-	-	-	-
BC024619	Hydroxypyruvate isomerase homolog (Hyi)	-	-2.3	-	-	-	-
BB546344	Hydroxysteroid (17-beta) dehydrogenase 11 (Hsd17b11)	-	-2	-	-	-	-
BC012682	Hydroxysteroid (17-beta) dehydrogenase 2 (Hsd17b2)	-	-2	-	-	-	-
X95580	Hypoxia inducible factor 1, alpha subunit (Hif1a)	-	2.5	-	-	-	-
AK015966	IBR domain containing 3 (Ibrdc3)	-	2.2	-	2	-	-
AK009564	IKAROS family zinc finger 5 (Ikzf5)	-	2.5	-	-	-	-
NM_133662	Immediate early response 3 (Ier3)	-	13.1	-	-	-	-
BF147705	Immediate early response 5 (Ier5)	-	3.5	-	-	-	-
NM_008326	Immunity-related GTPase family, M (Irgm)	-	2.8	-	4.2	-	-
BC018365	Immunoglobulin heavy chain 6 (heavy chain of IgM) (Igh-6)	-	-	-	-	51.4	31.9
AK008551	Immunoglobulin lambda chain, variable 1 (Igl-V1)	-	-	-	-2.2	-	-
NM_030691	Immunoglobulin superfamily, member 6 (Igsf6)	-	3.1	-	2.8	-	-
L38281	Immunoresponsive gene 1 (Irg1)	-	8.7	-	10.9	-	-
NM_008324	Indoleamine-pyrrole 2,3 dioxygenase (Indo)	-	3.3	-	6.4	-	-
BC026393	Indoleamine-pyrrole 2,3 dioxygenase-like 1 (Indol1)	-	-2.3	-	-	-	-
BB253137	Inhibin beta-B (Inhbb)	-	4.6	-	-	-	-
AK013239	Inhibitor of DNA binding 2 (Id2)	-3.8	-	-	-	2.1	2.3

NM_019777	Inhibitor of kappaB kinase epsilon (Ikbke)	-	2.8	-	-	-	-
BB081797	Inositol polyphosphate multikinase (Ipmk)	-	-2.1	-	-	-	-
BB345784	Insulin receptor substrate 1 (Irs1)	-	-2.6	-	-	-	-
NM_008341	Insulin-like growth factor binding protein 1 (Igfbp1)	-	3.7	-	-	-	-
NM_008344	Insulin-like growth factor binding protein 6 (Igfbp6)	-	3.2	-	-	-	-
NM_008340	Insulin-like growth factor binding protein, acid labile subunit (Igfals)	-	-7.4	-	-	-	-
AV251853	Integrin alpha FG-GAP repeat containing 3 (Itfg3)	-	-2.2	-	-	-	-
NM_021334	Integrin alpha X (Itgax)	-	2.3	-	-	-	-
BB443308	Integrin beta 1 (fibronectin receptor beta) (Itgb1)	-	3.8	-	-	-	-
NM_008404	Integrin beta 2 (Itgb2)	-	3.3	-	-	-	-
NM_021359	Integrin beta 6 (Itgb6)	-	2.2	-	-	-	-
AK004893	Inter alpha-trypsin inhibitor, heavy chain 4 (Itih4)	-	-	2.7	-	-	-
BC008626	Intercellular adhesion molecule (Icam1)	-	12.1	-	-	-	-
NM_010494	Intercellular adhesion molecule 2 (Icam2)	-	2.6	-	-	-	-
BB522265	Interferon (alpha and beta) receptor 2 (Ifnar2)	-	2.8	-	-	-	-
NM_011940	Interferon activated gene 202B (Ifi202b)	-	7.4	-	2.9	-	3.5
BC008167	Interferon activated gene 203 (Ifi203)	-	3.5	-	-	-	-
K00083	Interferon gamma (Ifng)	-	4.2	-	4.3	-	5.4
NM_018738	Interferon gamma induced GTPase (Igtp)	-	4	-	7.8	-	2.4
NM_008330	Interferon gamma inducible protein 47 (Ifi47)	-	6	-	4.2	-	-
BC027285	Interferon induced transmembrane protein 1 (Ifitm1)	-	3.3	-	2	-	3.1
BB193024	Interferon induced transmembrane protein 6 (Ifitm)	-	-	-	4.4	-	-
BM239828	Interferon inducible GTPase 1 (Iigp1)	-	2	-	7.3	-	6.5
NM_019440	Interferon inducible GTPase 2 (Iigp2)	-	3.8	-	5.4	-	2.3
NM_008390	Interferon regulatory factor 1 (Irf1)	-	3.3	-	3.6	-	-
NM_016851	Interferon regulatory factor 6 (Irf6)	-	-2.4	-	-	-	-
BG069095	Interferon regulatory factor 8 (Irf8)	-	3.6	-	-	-	-
NM_008332	Interferon-induced protein with tetratricopeptide repeats 2 (Ifit2)	-	3.4	-	-	-	-
NM_013562	Interferon-related developmental regulator 1 (Ifrd1)	-	4.9	-	-	-	-
BC022751	Interferon-stimulated protein (Isg20)	-	4.4	-	-	-	-
BC003727	Interleukin 1 alpha (IL1a)	-	3.1	-	-	-	-
BC011437	Interleukin 1 beta (IL-1b)	-	7.2	-	7.1	-	2.5
NM_031167	Interleukin 1 receptor antagonist (IL1rn)	-	8.1	-	-	-	-
M57525	Interleukin 1 receptor antagonist (Ilrn1)	-	5.4	-	3.8	-	-
NM_008362	Interleukin 1 receptor, type I (IL1r1)	-	10.4	-	-	-	-
NM_010555	Interleukin 1 receptor, type II (IL-1r2)	-	3.7	-	5.2	-	4.2
AF128214	Interleukin 12b (IL12b)	-	2.3	-	-	-	-
AK010040	Interleukin 17 receptor A (IL17ra)	-	2.6	-	-	-	-
AF110803	Interleukin 18 binding protein (Il18bp)	-	-	-	2.7	-	-
NM_008365	Interleukin 18 receptor 1 (IL18r1)	-	5.1	-	-	-	-

L20048	Interleukin 2 receptor, gamma chain (IL2rg)	-	2.9	-	-	-	-
NM_133775	Interleukin 33 (IL33)	-	5.8	-	-	-	-
NM_010557	Interleukin 4 receptor, alpha (IL4ra)	-	2	-	-	-	-
NM_031168	Interleukin 6 (IL-6)	-	51.6	3.8	34.3	-	-
AK017211	Interleukin 6 signal transducer (IL6st)	-	2.3	-	-	-	-
AI573431	Interleukin 7 receptor (IL7r)	-	-	-	-	2.2	-
NM_009909	Interleukin 8 receptor beta (ILRB)	-	-	-	2.9	-	-
AV228493	Interleukin-1 receptor-associated kinase 3 (Irak3)	-	9	-	-	-	-
NM_016721	IQ motif containing GTPase activating protein 1 (Iqgap1)	-	4	-	-	-	-
AF165984	Iroquois related homeobox 1 (Irx1)	-	2	-	-	-	-
NM_008393	Iroquois related homeobox 3 (Irx3)	-	4.2	-	-	-	-
AV221988	Isovaleryl coenzyme A dehydrogenase (Ivd)	-	-2	-	-	-	-
AV293164	Jumonji domain containing 1C (Jmjdc1)	-	2.3	-	-	-	-
BG228765	Jumonji domain containing 3 (Jmjd3)	-	-	-	-	2.2	-
NM_010591	Jun oncogene (Jun)	-	9.4	-	-	-	-
NM_008416	Jun-B oncogene (Junb)	-	5.1	-	-	-	-
BC026555	Kallikrein B, plasma 1 (Klkb1)	-	-4.2	-	-	-	-
BB469300	Kelch repeat and BTB (POZ) domain containing 8 (Kbtbd8)	-	2.4	-	-	-	-
BB428573	Kelch-like 2, Mayven (Klhl2)	-	2.7	-	-	-	2.9
BM124262	Kelch-like 24 (Klhl24)	-	-	-	-	2.1	-
NM_008469	Keratin 15 (Krt15)	-	-	-	-2.1	-	-
NM_008471	Keratin 19 (Krt19)	-	2.6	-	-	-	-
BC010337	Keratin 7 (Krt7)	-	6.6	-	-	-	-
NM_028770	Keratin 80 (Krt80)	-	2.2	-	-	-	-
NM_029550	Kidney expressed gene 1 (Keg1)	-	-6.7	-	-	-	-
BB342219	Kinesin family member 21A	-	2.2	-	-	-	-
BI328541	Kinesin family member 5B	-	2	-	-	-	-
BB815530	Kit ligand	-	3.9	-	-	-	-
X65997	Kit oncogene	-	2.6	-	-	-	-
NM_031180	Klotho beta	-	-2.6	-	-	-	-
BC013486	Kruppel-like factor 15 (Klf15)	-	-	-	2.4	-	-
NM_008452	Kruppel-like factor 2 (lung) (Kif21a)	-	2.8	-	-	-	-
BG069413	Kruppel-like factor 4 (gut))Kif4)	-	6.7	-	-	-	-
BI465857	Kruppel-like factor 5 (Kif5)	-	2.6	-	-	-	-
AV025472	Kruppel-like factor 6 (Kif6)	-	4.4	-	-	-	-
BB524597	Kruppel-like factor 7 (ubiquitous) (Kif7)	-	2.2	-	-	-	-
NM_010699	Lactate dehydrogenase A (Ldha)	-	2	-	-	-	-
AV219418	Lactate dehydrogenase B (Ldhb)	-	2.4	-	-	-	-
NM_008522	Lactotransferrin (Ltf)	-	3	-	-	-	-
C77434	Lanosterol synthase (Lss)	2.1	-	-	-	-	-
AV271979	Large tumor suppressor 2 (Lats2)	-	2.2	-	-	-	-
X16834	Lectin, galactose binding, soluble 3 (Lgals3)	-	4.4	-	-	-	-
BC011507	Lectin, galactose binding, soluble 7 (Lgals7)	-	2.4	-	-	-	-
BE283629	Leprecan 1 (Lepre1)	-	2.1	-	-	-	-
BM124366	Leptin receptor (Lepr)	-	-	-	-	2.1	-
BG069059	Leucine rich repeat (in FLII) interacting protein 1 (Lrrfip1)	-	2.3	-	-	-	-

AI413999	Leucine rich repeat containing 3 (Lrrc3)	-	-2.4	-	-	-	-
AW476171	Leucine rich repeat containing 59 (Lrrc59)	-	2.4	-	-	-	-
NM_029796	Leucine-rich alpha-2-glycoprotein 1 (Lrg1)	-	3.2	-	-	-	-
AK014938	Leucine-rich repeat kinase 2 (Lrrk2)	-	2.6	-	-	-	-
AV359781	Leucine-rich repeats and transmembrane domains 1 (Lrtm1)	-	-3.6	-	-	-	-
NM_010701	Leukocyte cell derived chemotaxin 1 (Lect1)	-	-2.3	-	-	-	-
NM_010702	Leukocyte cell-derived chemotaxin 2 (Lect2)	-	-3.7	-	-	-	-
BM231903	LIM domain only 7 (Lmo7)	-	2.8	-	-	-	-
AV281676	Lin-7 homolog A (Lin7a)	-	-2.6	-	-	-	-
BC020991	Lipase, endothelial (Lipg)	-	-	-	-	-	3.7
BB367422	Lipase, member H (Liph)	-	2.7	-	-	-	-
AK014526	Lipin 1 (Lpin1)	-	2.3	-	-	-	-
X14607	Lipocalin 2 (Lcn2)	6.5	67.3	5.7	6	-	-
AV149705	Lipoma HMGIC fusion partner (Lhfp)	-	2.4	-	-	-	-
**AW208574**	**Lipopolysaccharide binding protein (Lbp)**	**-**	**4.2**	**-**	**-**	**-**	**-**
AK017272	Lipoprotein lipase (Lpl)	-	2.9	-	-	-	-
NM_133198	Liver glycogen phosphorylase (Pygl)	-	-2.2	-	-	-	-
BQ176664	Longevity assurance homolog 6 (Lass6)	-	3.4	-	-	-	-
BC004727	Loss of heterozygosity, 11, chromosomal region 2, gene A homolog (Loh11cr2a)	-	2.2	-	-	-	-
AV360881	LPS-induced TN factor (Litaf)	-	2.6	-	-	-	-
BI662680	LSM14 homolog A (Lsm14a)	-	2	-	-	-	-
AV309996	Luc7 homolog (S. cerevisiae)-like (Luc7l)	-	-	-	-	2	-
BG070818	LUC7-like 2 (Luc7l2)	-	2.2	-	-	-	-
BC002070	Lymphocyte antigen 6 complex, locus A (Ly6a)	-	6	-	-	-	-
NM_010741	Lymphocyte antigen 6 complex, locus C (Ly6c)	-	13.1	-	2.7	-	-
AF232024	Lymphocyte antigen 6 complex, locus I (Ly6i)	-	2.9	-	2.6	-	-
NM_008879	Lymphocyte cytosolic protein 1 (Lcp1)	-	3	-	-	-	-
BC006948	Lymphocyte cytosolic protein 2 (Lcp2)	-	2.8	-	-	-	-
BB667084	LysM, putative peptidoglycan-binding, domain containing 3 (Lysmd3)	-	3.2	-	-	-	-
BE987427	Lysophosphatidylglycerol acyltransferase 1 (Lpgat1)	-	2.7	-	-	-	-
AI596237	Lysosomal acid lipase 1 (Lio1)	-	-2.3	-	-	-	-
AW208566	Lysozyme (Lyzs)	-	5.9	-	-	-	-
M65143	Lysyl oxidase (Lox)	-	5.9	-	2.8	-	-
AF357006	Lysyl oxidase-like 1 (Loxl1)	-	2.9	-	-	-	-
BG092512	Macrophage activation 2 like (Mpa2l)	-	6.1	-	7.9	-	5.1
L20315	Macrophage expressed gene 1 (Mpeg1)	-	2.3	-	-	-	-
NM_010766	Macrophage receptor with collagenous structure (Marco)	-	5.4	-	-	-	-
NM_010760	Mago-nashi homolog, proliferation-associated (Magoh)	-	2	-	-	-	-
AK006096	Major facilitator superfamily domain containing 2 (Mfsd2)	2.8	2.6	-	-	-	-
NM_080638	Major vault protein (Mvp)	-	2.7	-	-	-	-
BG094874	Male sterility domain containing 2 (Mlstd2)	-	2.3	-	-	-	-

NM_010807	MARCKS-like 1 (Marcksl1)	-	2.2	-	2.7	-	-
AY042192	MAS-related GPR, member A2 (Mrgpra2)	-	-	-	2.5	-	-
NM_008597	Matrix Gla protein (Mgp)	-	7.9	-	-	-	-
AF153199	Matrix metallopeptidase 19 (Mmp19)	-	-2.1	-	-	-	-
BF147716	Matrix metallopeptidase 2 (Mmp2)	-	2.3	-	-	-	-
NM_010809	Matrix metallopeptidase 3 (Mmp3)	-	2.5	-	-	-	-
NM_008611	Matrix metallopeptidase 8 (Mmp8)	-	4.5	-	5.6	-	-
AV228517	MAX dimerization protein 1 (Mxd1)	-	3.2	-	2.4	-	-
AV293368	Mcf.2 transforming sequence-like (Mcf2l)	-	2.8	-	-	-	-
NM_019453	Mediterranean fever (Mefv)	-	2.6	-	2.6	-	-
BB795191	Melanoma antigen (Mela)	-	2.8	-	-	-	-
NM_023061	Melanoma cell adhesion molecule (Mcam)	-	2.1	-	-	-	-
AV298358	Melanoregulin (Mreg)	-	-3	-	-	-	-
BB548141	Membrane targeting (tandem) C2 domain containing 1 (Mtac2d1)	-	2.9	-	-	-	-
NM_022429	Membrane-spanning 4-domains, subfamily A, member 4C (Ms4a4c)	-	10	-	-	-	-
NM_025658	Membrane-spanning 4-domains, subfamily A, member 4D (Ms4a4d)	-	-	-	-	-	4.4
NM_027209	Membrane-spanning 4-domains, subfamily A, member 6B (Ms4a6b)	-	7.5	-	-	-	-
NM_026835	Membrane-spanning 4-domains, subfamily A, member 6D (Ms4a6d)	-	5.8	-	-	-	4.5
NM_138670	Mercaptopyruvate sulfurtransferase (Mpst)	-	-2.3	-	-	-	-
NM_013602	Metallothionein 1 (Mt1)	-	3	-	7.2	-	-
AA796766	Metallothionein 2 (Mt2)	-	6.9	4.1	16.5	-	-
BB326749	Metastasis suppressor 1 (Mtss1)	-	2.3	-	-	-	-
BC021619	Methionine sulfoxide reductase B2 (Msrb2)	-	-2	-	-	-	-
BB277041	Methionine sulfoxide reductase B3 (Msrb3)	-	2	-	-	-	-
AK007371	Methyl-CpG binding domain protein 1 (Mbd1)	-	2.3	2.7	3.4	-	-
BG076333	Methylenetetrahydrofolate dehydrogenase (NAD+ dependent), methenyltetrahydrofolate cyclohydrolase (Mthfd2)	-	3.8	3.1	-	-	-
NM_138745	Methylenetetrahydrofolate dehydrogenase (NADP+ dependent), methenyltetrahydrofolate cyclohydrolase, formyltetrahydrofolate synthase (Mthfd1)	-	-2.5	-	-	-	-
BG075139	Methylthioadenosine phosphorylase (Mtap)	-	-2.7	-	-	-	-
NM_027853	Methyltransferase like 7B (Mettl7b)	-	-2.6	-	-	-	-
BC004636	Methyltransferase like 8 (Mettl8)	-	-2.3	-	-	-	-
BC022666	Microfibrillar-associated protein 4 (Mfap4)	-	2.9	-	-	-	-
BC026506	Microrchidia 3 (Morc3)	-	2.4	-	-	-	-
NM_025569	Microsomal glutathione S-transferase 3 (Mgst3)	-	-2.3	-	-	-	-
AF026565	Midline 1 (Mid1)	-	2.5	-	-	-	-
BC020137	MIT, microtubule interacting and transport, domain containing 1 (Mitd1)	-	2	-	-	-	-
NM_053162	Mitochondrial ribosomal protein L34 (Mrpl34)	-	-2.1	-	-	-	-
NM_007746	Mitogen activated protein kinase kinase kinase 8 (Map3k8)	-	3.3	-	-	-	-

NM_016693	Mitogen-activated protein kinase kinase kinase 6 (Map3k6)	-	-	-	3.5	-	-
BG067961	Mitogen-activated protein kinase kinase kinase kinase 5 (Map4k5)	-	2.1	-	-	-	-
BM196656	MKL (megakaryoblastic leukemia)/myocardin-like 1 (Mkl1)	-	2.4	-	-	-	-
AF265663	MLX interacting protein (Mlxip)	-	2.8	-	-	-	-
AF245479	MLX interacting protein-like (Mlxipl)	-	-2.7	-	-	-	-
BE288498	MOB1, Mps One Binder kinase activator-like 2C (Mobkl2c)	-	2	-	-	-	-
NM_010833	Moesin (Msn)	-	3.1	-	-	-	-
BC021914	Monocyte to macrophage differentiation-associated (Mmd)	-	-2.4	-	-	-	-
AV269411	Monocyte to macrophage differentiation-associated 2 (Mmd2)	-	-3	-	-	-	-
BB359043	MTERF domain containing 3 (Mterf3d)	-	-2	-	-	-	-
NM_013605	Mucin 1, transmembrane (Muc1)	-	5.1	-	-	-	-
BB485735	Mucosa associated lymphoid tissue lymphoma translocation gene 1 (Malt1)	-	2.7	-	-	-	-
BI649713	Muscleblind-like 2 (Mbnl2)	-	2.1	-	-	-	-
NM_139063	Muted homolog (Muted)	-	-2.2	-	-	-	-
AI642973	Myc target 1 (Myct1)	-	-	-	-2.7	-	-
NM_010762	Myelin and lymphocyte protein, T-cell differentiation protein (Mal)	-	2.5	-	-	-	-
BC003839	Myeloid cell leukemia sequence 1 (Mcl1)	-	2.3	-	-	-	-
NM_008654	Myeloid differentiation primary response gene 116 (Myd116)	-	2.1	-	-	-	-
BC005591	Myeloid differentiation primary response gene 88 (Myd88)	-	3.4	-	-	-	-
BF021054	Myeloid/lymphoid or mixed-lineage leukemia 5 (Mll5)	-	-	-	-	2	-
NM_023627	Myo-inositol 1-phosphate synthase A1 (Isyna1)	-	4.7	-	-	-	-
C80049	Myosin, heavy polypeptide 9, non-muscle (Myh9)	-	2	-	-	-	-
BB454540	Myristoylated alanine rich protein kinase C substrate (Marcks)	-	2.4	-	-	-	-
BI106821	N-acylsphingosine amidohydrolase (acid ceramidase)-like (Asahl)	-	2	-	-	-	-
AF282864	N-acylsphingosine amidohydrolase 3-like (Asah3l)	-	3.7	-	-	-	-
NM_020282	NAD(P)H dehydrogenase, quinone 2 (Nqo2)	-	-3.7	-	-	-	-
AW105774	Napsin A aspartic peptidase (Napsa)	-	7.4	-	-	-	-
BB311061	Nasal embryonic LHRH factor (Nelf)	-	-2.6	-	-	-	-
BM121794	Nebulette (Nebl)	-	7.5	-	-	-	-
AA223007	Nephronectin (Npnt)	-	8.1	-	-	-	-
NM_017464	Neural precursor cell expressed, developmentally down-regulated gene 9 (Nedd9)	-	4	-	-	-	-
BB219343	Neuregulin 4 (Nrg4)	-	4.1	-	-	-	-

NM_019867	Neuronal guanine nucleotide exchange factor (Ngef)	-	-2.6	-	-	-	-
BG070037	Neuronal PAS domain protein 2 (Npas2)	-2.1	-	-	-	-	-
AI844633	Neutrophil cytosolic factor 1 (Ncf1)	-	3.1	-	-	-	-
NM_008677	Neutrophil cytosolic factor 4 (Ncf4)	-	2.2	-	-	-	-
NM_008694	Neutrophilic granule protein (Ngp)	-	3.9	-	-	-	-
AV031928	Nicolin 1 (Nicn1)	-	-2.1	-	-	-	-
AK006371	Nicotinamide N-methyltransferase (Nnmt)	4.8	10	-	-	-	-
BB205930	Nicotinamide nucleotide transhydrogenase (Nnt)	-	-	-	-	2	-
X14480	Nidogen 1 (Nid1)	-	2.3	-	-	-	-
BB528391	NIMA (never in mitosis gene a)-related expressed kinase 6 (Nek6)	-	2	-	-	-	-
AK014427	NIPA-like domain containing 1 (Npal1)	-	-2.5	-	-	-	-
AV309418	N-myc downstream regulated 1 (Ndrg)	-2.1	-	-	-	-	-
NM_010878	Non-catalytic region of tyrosine kinase adaptor protein 1 (Nck1)	-	2.7	-	-	-	-
NM_010929	Notch gene homolog 4 (Notch4)	-	2.5	-	-	-	-
AK004737	NUAK family, SNF1-like kinase, 2 (Nuak2)	-	-	-	2.3	-	-
BB533448	Nuclear factor I/C (Nfic)	-	-2	-	-	-	-
AW049660	Nuclear factor I/X (Nfix)	-	-2	-	-	-	-
BB096843	Nuclear factor of kappa light chain gene enhancer in B-cells inhibitor, alpha (Nfkbia)	-	4.1	-	2.3	-	-
AF155372	Nuclear factor of kappa light polypeptide gene enhancer in B-cells 2, p49/p100 (Nfkb2)	-	2.3	-	-	-	-
BB820441	Nuclear factor of kappa light polypeptide gene enhancer in B-cells inhibitor, epsilon (Nfkbie)	-	5.3	-	-	-	-
AB026551	Nuclear factor of kappa light polypeptide gene enhancer in B-cells inhibitor, zeta (Nfkbiz)	-	4.3	-	2.1	-	-
AY061760	Nuclear factor, interleukin 3, regulated (Nfil3)	-	-	3.4	3	-	2
BC003972	Nuclear import 7 homolog (Nip7)	-	2.3	-	-	-	-
NM_019738	Nuclear protein 1 (Nupr1)	-	3.6	4.8	-	-	-
BC019540	Nuclear receptor subfamily 0, group B, member 2 (Nr0b2)	-	-5.7	-	-	-	-
W13191	Nuclear receptor subfamily 1, group D, member 1 (Nr1d1)	-3.7	-	-	-	-	-
NM_011584	Nuclear receptor subfamily 1, group D, member 2 (Nr1d2)	-	-	-2.8	-	-	-
AF009328	Nuclear receptor subfamily 1, group I, member 3 (Nrli3)	-	-4.6	-	-	-	-
NM_010150	Nuclear receptor subfamily 2, group F, member 6 (Nr2f6)	-	-2	-	-	-	-
BB096079	Nuclear receptor subfamily 3, group C, member 1 (Nr3c1)	-	-	-	-	2.4	-
NM_016773	Nucleobindin 2 (Nucb2)	-	3.3	-	-	-	-
BB729616	Nucleolar protein 5 (Nol5)	-	4	-	-	-	-
BC026743	Nucleoporin like 1 (Nupl1)	-	2	-	-	-	-

NM_008637	Nudix (nucleoside diphosphate linked moiety X)-type motif 1 (Nudt1)	-	-2.7	-	-	-	-
AK008824	Nudix (nucleoside diphosphate linked moiety X)-type motif 7 (Nudt7)	-	-2.6	-	-	-	-
NM_025529	Nudix (nucleoside diphosphate linked moiety X)-type motif 8 (Nudt8)	-	-2	-	-	-	-
AV290148	Olfactomedin 4 (Olfm4)	-	3.8	-	7.3	-	-
AB015978	Oncostatin M receptor (Osmr)	-	2.9	-	-	-	-
NM_008262	One cut domain, family member 1 (Onecut1)	2.2	-	-	-	-	-
NM_011014	Opioid receptor, sigma 1 (Oprs1)	-	-2.8	-	-	-	-
BC024893	Ornithine transcarbamylase (Otc)	-	-3.2	-	-	-	-
BE628912	Orosomucoid 1 (Orm1)	-	2.2	-	-	-	-
NM_011016	Orosomucoid 2 (Orm2)	-	13.3	3.6	3.2	-	-
BB143568	Orphan short chain dehydrogenase/reductase (Sdro)	-	-2.9	-	-	-	-
BB542051	Osteoglycin (Ogn)	-	5	-	-	-	-
BC022135	Oxidative stress induced growth inhibitor 1 (Osgin1)	-2.1	-4.9	-	-	-	-
NM_138648	Oxidized low density lipoprotein (lectin-like) receptor 1 (Olr1)	-	3.5	-	-	-	-
AK004768	Oxysterol binding protein-like 3 (Osbpl3)	-	2.1	-	-	-	-
AV058500	P lysozyme structural (L-zps)	-	7.9	-	-	-	-
AA185884	Paired box gene 5 (Pax5)	-	-	-	-	2.2	-
NM_011093	Paired-Ig-like receptor A1 (Pira1)	-	3.5	-	-	-	-
NM_011087	Paired-Ig-like receptor A2 (Pira2)	-	2.5	-	-	-	-
BC013446	Parathyroid hormone receptor 1 (Pthr1)	-	-2.9	-	-	-	-
NM_019999	Paroxysmal nonkinesiogenic dyskinesia (Pnkd)	-	-3.6	-	-	-	-
BC024556	PDZ and LIM domain 2 (Pdlim2)	-	3.7	-	-	-	-
AK006269	PDZ domain containing 1 (Pdzk1)	-	-2.2	-	-	-	-
AV376136	PDZ domain containing 2 (Pdzd2)	-	4.3	-	-	-	-
NM_018884	PDZ domain containing RING finger 3 (Pdzrn3)	-	3.1	-	-	-	-
NM_018832	PDZ domain containing, X chromosome (Pdzx)	-	-2.1	-	-	-	-
NM_008987	Pentraxin related gene (Ptx3)	-	3.2	-	3.3	-	-
NM_009402	Peptidoglycan recognition protein 1 (Pglyrp1)	-	5.4	-	-	-	-
NM_008908	Peptidylprolyl isomerase C (Ppic)	-	3.4	-	-	-	-
BC011499	Peptidylprolyl isomerase D (cyclophilin D) (Ppid)	-	2.8	-	-	-	-
AF022992	Period homolog 1 (Per1)	2.6	2.8	-	-	-	-
AF035830	Period homolog 2 (Per2)	4.1	-	-	-	-	-
NM_011067	Period homolog 3 (Per3)	2.5	-	-	-	-	-
NM_008885	Peripheral myelin protein (Pmp22)	-	6.9	-	-	-	-
NM_011068	Peroxisomal biogenesis factor 11a (Pex11a)	-	-	-	-	-	2.5
AF309644	Peroxisomal membrane protein 2 (Pxmp2)	-	-2.4	-	-	-	-
NM_133249	Peroxisome proliferative activated receptor, gamma, coactivator 1 beta (Ppargc1b)	-	-2.2	-	-	-	-
BM199989	Peroxisome proliferative activated receptor, gamma, coactivator-related 1 (Pprc1)	-	3.7	-	-	-	-

AI327038	PFTAIRE protein kinase 1 (Pftk1)	-	2.7	-	-	-	-
BF715219	PHD finger protein 20-like 1 (Phf20l1)	-	2.6	-	-	-	-
NM_021451	Phorbol-12-myristate-13-acetate-induced protein 1 (Pmaip1)	-	7	-	2.6	-	-
NM_008903	Phosphatidic acid phosphatase 2a (Ppap2a)	-	2.1	-	-	-	-
NM_011083	Phosphatidylinositol 3-kinase, C2 domain containing, alpha polypeptide (Pik3c2a)	-	2	-	-	-	-
NM_011084	Phosphatidylinositol 3-kinase, C2 domain containing, gamma polypeptide (Pik3c2g)	-	-2.9	-	-	-	-
AV340508	Phosphatidylinositol glycan anchor biosynthesis, class A (Piga)	-	2.2	-	-	-	-
AF030178	Phosphatidylinositol glycan anchor biosynthesis, class Q (Pigq)	-	-	-	-	-2.5	-
AK003713	Phosphatidylinositol glycan anchor biosynthesis, class Y (Pigy)	-	-2	-	-	-	-
BM246564	Phosphodiesterase 4B, cAMP specific (Pde4b)	-	2.3	-	-	-	-
BE370618	Phosphodiesterase 5A, cGMP-specific (Pde5a)	-	3.7	-	-	-	-
NM_008804	Phosphodiesterase 9A (Pde9a)	-	-2.4	-	-	-	-
BB076574	Phosphofructokinase, platelet (Pfkp)	-	2	-	-	-	-
BI684288	Phosphoinositide-3-kinase adaptor protein 1 (Pik3ap1)	-	2.5	-	-	-	-
BM730668	Phospholipase C, beta 4 (Plcb4)	-	3.2	-	-	-	-
BF319989	Phospholipid scramblase 1 (Plscr1)	-	5.9	-	-	-	-
BB826296	Phospholipid scramblase 4 (Plscr4)	-	-	-	-2.1	-	-
BB307726	Phosphorylase kinase alpha 2 (Phka2)	-	-2	-	-	-	-
BC004827	Phosphoserine aminotransferase 1 (Psat1)	-	4.3	-	-	-	-
AF263458	Placenta-specific 8 (Plac8)	-	5.5	-	-	-	-
AV286396	Plakophilin 4 (Pkp4)	-	-2.1	-	-	-	-
NM_008872	Plasminogen activator, tissue (Plat)	-	4.1	-	-	-	-
X62701	Plasminogen activator, urokinase receptor (Plaur)	-	2.3	-	-	-	-
AW537708	Platelet derived growth factor receptor, alpha polypeptide (Pdgfra)	-	5.1	-	-	-	-
BC023427	Platelet derived growth factor, B polypeptide (Pdgfb)	-	2.5	-	-	-	-
NM_008816	Platelet/endothelial cell adhesion molecule 1 (Pecam1)	-	3	-	-	-	-
NM_019971	Platelet-derived growth factor, C polypeptide (Pdgfc)	-2.4	-	-	-	-	-
AF181829	Pleckstrin (Plek)	-	2.5	-	-	-	-
NM_133244	Pleckstrin homology domain containing, family K member 1 (Plekhk1)	-	3.1	-	-	-	-
NM_011182	Pleckstrin homology, Sec7 and coiled-coil domains 3 (Pscd3)	-	2.1	-	-	-	-
BB503614	Pleckstrin homology, Sec7 and coiled-coil domains, binding protein (Pscdcp)	-	5.3	-	-	-	-
BB476707	Plexin C1 (Plxnc1)	-	-	-	-	2.4	-
BB559706	Plexin domain containing 2 (Plxdc2)	-	2.6	-	-	-	-

AF290209	Podocalyxin-like (Pdxl)	-	2.5	-	-	-	-
NM_010329	Podoplanin (Pdpn)	-	5.1	-	-	-	-
NM_009310	Poliovirus receptor (Pvr)	-	2.8	-	-	-	-
BM230222	Poly (A) polymerase alpha (Papola)	-	2	-	-	-	-
BM227980	Poly (ADP-ribose) polymerase family, member 12 (Parp12)	-	-	-	-	-	2.9
BC021340	Poly (ADP-ribose) polymerase family, member 14 (Parp14)	-	3.5	-	2.7	-	-
BC012674	Polymerase I and transcript release factor (Ptrf)	-	2.4	-	-	-	-
BM195033	Polypyrimidine tract binding protein 2 (Ptbp2)	-	-	-2.2	-	-	-
BC006935	Potassium channel tetramerisation domain containing 10 (Kctd10)	-	2.2	-	-	-	-
AF319542	Potassium channel, subfamily K, member 5 (Kcmk5)	-	-2.3	-	-	-	-
BB533892	Potassium inwardly-rectifying channel, subfamily J, member 15 (Kcnj15)	-	5.3	-	-	-	-
NM_021342	Potassium voltage-gated channel, Isk-related subfamily, gene 4 (Kcne4)	-	2.2	-	-	-	-
BM250850	Potassium voltage-gated channel, subfamily Q, member 1 (Kcnq1)	-	2.3	-	-	-	-
AW061073	PQ loop repeat containing 1 (Pqlc1)	-	-2.3	-	-	-	-
AV349236	PR domain containing 8 (Prdm8)	-	-	-	-2.7	-	-
AW989410	Pre-B-cell colony-enhancing factor 1 (Pbef1)	2.2	4.1	-	2.4	-	2.5
AF408412	Premature ovarian failure 1B (Pof1b)	-	2.6	-	-	-	-
BB170718	Prickle-like 2 (Prickle2)	-	-	-	-2.9	-	-
AF352788	Procollagen C-endopeptidase enhancer 2 (Pcocle2)	-	3.7	-	-	-	-
BC021352	Procollagen lysine, 2-oxoglutarate 5-dioxygenase 2 (Plod2)	-	4.2	-	-	-	-
BF158638	Procollagen, type IV, alpha 1 (Col4a1)	-	3.5	-	-	-	2.1
BC013560	Procollagen, type IV, alpha 2 (Col4a2)	-	3.7	-	-	-	-
AV366831	Procollagen, type IV, alpha 3 (Col4a3)	-	3.7	-	-	-	-
BB530633	Procollagen, type IV, alpha 4 (Col4a4)	-	3.3	-	-	-	-
NM_009933	Procollagen, type VI, alpha 1 (Col6a1)	-	2	-	-	-	-
AK018174	Progestin and adipoQ receptor family member III (Paqr3)	-	2.1	-	-	-	-
AK010619	Prohibitin (Phb)	-	-2	-	-	-	-
BE651535	Prokineticin 1 (Prok1)	-	-3.5	-	-	-	-
NM_015768	Prokineticin 2 (Prok2)	-	-	-	2.8	-	2.6
NM_008932	Prolactin receptor (Prlr)	-	-2.7	-	-	-	-
AK011039	Proline dehydrogenase (oxidase) 2 (Prodh2)	-	-2	-	-	-	-
NM_011172	Proline dehydrogenase (Prodh)	-	-2.4	-	-	-	-
BI410130	Proline-rich nuclear receptor coactivator 1 (Pnrc1)	-	2.1	-	-	-	-
BB241731	Proprotein convertase subtilisin/kexin type 5 (Pcsk5)	-	2	-	-	-	-
BB427703	ProSAPiP1 protein (Prosapip1)	-	-2.4	-	-	-	-
BC011193	Prostaglandin E receptor 4 (subtype EP4) (Ptger4)	-	2.5	-	-	-	-

AV253087	Prostaglandin F2 receptor negative regulator (Ptgfrn)	-	3.9	-	-	-	-
M94967	Prostaglandin-endoperoxide synthase 2 (Ptgs2)	-	20.8	-	-	-	-
BB378796	Protease, serine, 23 (Prss23)	-	4.7	-	-	-	-
NM_011171	Protein C receptor, endothelial (Procr)	-	2.2	-	-	-	-
AF253414	Protein kinase C, epsilon (Prkce)	-	2.4	-	-	-	-
NM_008856	Protein kinase C, eta (Prkch)	-	2.1	-	-	-	-
AV047342	Protein kinase inhibitor beta, cAMP dependent, testis specific (Pkib)	-	-	-	-	-	2.1
BB216074	Protein kinase, cAMP dependent regulatory, type II beta (Prkar2b)	-	2.4	-	-	-	-
BB823350	Protein kinase, cGMP-dependent, type II (Prkg2)	-	2	-	-	-	-
BC006875	Protein kinase, X-linked (Prkx)	-	2.1	-	-	-	-
U89924	Protein phosphatase 1, regulatory (inhibitor) subunit 3C (Ppp1r3c)	-	-2.5	-	-	-	-
AJ271833	Protein phosphatase 1B, magnesium dependent, beta isoform (Ppm1b)	-	-2.1	-	-	-	-
AV290622	Protein phosphatase 2C, magnesium dependent, catalytic subunit (Ppm2c)	-	3	-	-	-	-
BB467915	Protein phosphatase 3, catalytic subunit, alpha isoform (Pp3ca)	-	-	-2	-	-	-
AV331223	Protein tyrosine phosphatase 4a1 (Ptp4a1)	-	2.7	-	-	-	-
BC010191	Protein tyrosine phosphatase, non-receptor type 1 (Ptpn1)	-	3.1	-	-	-	-
AV117227	Protein tyrosine phosphatase, non-receptor type 12 (Ptpn12)	-	2.1	-	-	-	-
NM_008977	Protein tyrosine phosphatase, non-receptor type 2 (Ptpn2)	-	2.2	-	-	-	-
NM_008979	Protein tyrosine phosphatase, non-receptor type 22 (lymphoid) (Ptpn22)	-	-	-	-	-	2.5
NM_011210	Protein tyrosine phosphatase, receptor type, C (Ptprc)	-	3.4	-	-	-	-
NM_011157	Proteoglycan 1, secretory granule (Prg1)	-	6.1	-	-	-	-
NM_021400	Proteoglycan 4 (megakaryocyte stimulating factor, articular superficial zone protein) (Prg4)	-	4.4	-	-	-	-
AK012816	Proteolipid protein 2 (Plp2)	-	3.2	-	-	-	-
NM_010724	Proteosome (prosome, macropain) subunit, beta type 8 (large multifunctional peptidase 7) (Psmb8)	-	2	-	-	-	-
NM_013585	Proteosome (prosome, macropain) subunit, beta type 9 (large multifunctional peptidase 2) (Psmb9)	-	2.2	-	3.1	-	-
AI323550	Proviral integration site 1 (Pim1)	-	3	-	2.5	-	-
BB206220	Proviral integration site 3 (Pim3)	-	3.2	-	2.5	-	-
AK003895	PRP38 pre-mRNA processing factor 38 (yeast) domain containing B (Prpf38b)	-	2	-	-	-	-

NM_025273	Pterin 4 alpha carbinolamine dehydratase/dimerization cofactor of hepatocyte nuclear factor 1 alpha (TCF1) 1 (Pcbd1)	-	-2	-	-	-	-
BB314559	Pumilio 1 (Pum1)	-	-	-	-	2.3	-
AK020001	Purinergic receptor P2Y, G-protein coupled 10 (P2ry10)	-	-	-	-	2	-
BC016131	PX domain containing serine/threonine kinase (Pvx)	-	2	-	-	-	-
AV086243	Pyruvate dehydrogenase kinase, isoenzyme 3 (Pdk3)	-	2.7	-	-	-	-
NM_013743	Pyruvate dehydrogenase kinase, isoenzyme 4 (Pdk4)	-	-	-	-	3.1	4.1
BB667651	Pyruvate kinase liver and red blood cell (Pklr)	-	-2.7	-	-	-	-
NM_011099	Pyruvate kinase, muscle (Pkm2)	-	3.5	-	-	-	-
NM_021881	Quaking (Qk)	-	2.2	-	-	-	-
BC017628	Queuine tRNA-ribosyltransferase domain containing 1 (Qtrtd1)	-	2.5	-	-	-	-
AK004880	Quiescin Q6 (Qscn6)	-	2	-	-	-	-
AI835553	Rab interacting lysosomal protein (Rilp)	-	-2.7	-	-	-	-
AW542340	RAB18, member RAS oncogene family (Rab18)	-	2	-	-	-	-
BC010248	RAB43, member RAS oncogene family (Rab43)	-	-2.3	-	-	-	-
BM214169	RAB8B, member RAS oncogene family (Rab8b)	-	3.6	-	-	-	-
NM_011233	RAD17 homolog (Rad17)	-	2.1	-	-	-	-
BB741897	Radical S-adenosyl methionine domain containing 2 (Rsad2)	-	2.4	-	-	-	-
NM_009058	Ral guanine nucleotide dissociation stimulator (Ralgds)	-	2.2	-	-	-	-
BM507707	RAN binding protein 2 (Ranbp2)	-	2.4	-	-	-	-
BB106402	RAN binding protein 9 (Ranbp9)	-	2.2	-	-	-	-
BM230524	Rap guanine nucleotide exchange factor (GEF) 5 (Rapgef5)	-	3.5	-	-	-	-
AV307311	Ras association (RalGDS/AF-6) and pleckstrin homology domains 1 (Raph1)	-	2.3	-	-	-	-
BC018275	Ras homolog gene family, member B (Rhob)	-	5.3	-	-	-	-
AI503490	Ras homolog gene family, member C (Rhoc)	-	2.1	-	-	-	-
AF309564	Ras homolog gene family, member J (Rhoj)	-	2.1	-	-	-	-
BC028444	Ras interacting protein 1 (Rasip1)	-	2.8	-	-	-	-
BM246972	RAS related protein 1b (Rap1b)	-	2.2	-	-	-	-
BB217136	RAS, dexamethasone-induced 1 (Rasd1)	-	2.3	-	2	-	-
BI134758	RasGEF domain family, member 1A (Rasgef1a)	-	2.9	-	-	-	-
BB003229	RasGEF domain family, member 1B (Rasgef1b)	-	2.4	-	-	-	-
BF020283	Rearranged L-myc fusion sequence (Rlf)	-	2.1	-	-	-	-
AF146523	Receptor (calcitonin) activity modifying protein 2 (Ramp2)	-	2.7	-	-	-	-

NM_138952	Receptor (TNFRSF)-interacting serine-threonine kinase 2 (Ripk2)	-	3.1	-	-	-	-
AK002562	Receptor accessory protein 6 (Reep6)	-	-3.4	-	-	-	-
BC024872	Receptor transporter protein 4 (Rtp4)	-	2.8	-	-	-	3.3
AV255458	Regulatory factor X, 4 (influences HLA class II expression) (Rfx4)	-2.6	-3	-	-	-	-
NM_031192	Renin 1 structural (Ren1)	2.9	-	-	-	-	-
NM_020509	Resistin like alpha (Retnla)	-	11.2	-	-	-	-
BG969810	Restin (Reed-Steinberg cell-expressed intermediate filament-associated protein) (Rsn)	-	-	-	-	2.3	-
BE996483	Reticulon 4 receptor-like 1 (Rtn4rl1)	-	-3.1	-	-	-	-
U27177	Retinoblastoma-like 1 (p107) (Rbl1)	-	2.3	-	-	-	-
NM_023462	Retinol binding protein 7, cellular (Rbp7)	-	-	-	-2.2	-	-
NM_009040	Retinol dehydrogenase 16 (Rdh16)	-	-2.9	-	-	-	-
NM_134006	Retinol dehydrogenase 5 (Rdh5)	-	-2	-	-	-	-
BE979765	Retinol dehydrogenase 9 (Rdh9)	-	-2.8	-	-	-	-
BE852181	Rho family GTPase 1 (Rnd1)	-	4	-	-	-	-
BC012262	Rho guanine nucleotide exchange factor (GEF) 3 (Arhgef3)	-	2	-	-	-	-
C76675	Ribosomal protein S6 (Rps6)	-	3.7	-	-	-	-
W91617	Ring finger (C3HC4 type) and KH domain containing 1 (Rkhd1)	-	2.2	-	-	-	-
NM_013916	Ring finger protein 12 (Rnf12)	-	2	-	-	-	-
BB667823	Ring finger protein 125 (Rnf125)	-2.3	-	-	-	-	-
BC004781	Ring finger protein 43 (Rnf43)	-	-2.4	-	-	-	-
BC021788	RNA binding protein with multiple splicing 2 (Rbpms2)	-	-2	-	-	-	-
AV304745	RRS1 ribosome biogenesis regulator homolog (Rrs1)	-	2	-	-	-	-
AV228630	RUN and FYVE domain containing 3 (Rufy3)	-	-2.1	-	-	-	-
NM_009821	Runt related transcription factor 1 (Runx1)	-	2	-	-	-	-
BC021916	S100 calcium binding protein A11 (calizzarin) (S100a11)	-	8.9	-	-	-	-
D00208	S100 calcium binding protein A4 (S100a4)	-	3.2	-	-	-	-
NM_011313	S100 calcium binding protein A6 (calcyclin) (S100a6)	-	16.7	-	-	-	-
NM_013650	S100 calcium binding protein A8 (calgranulin A) (S100a8)	-	39.9	-	4.6	-	-
NM_009114	S100 calcium binding protein A9 (calgranulin A) (S100a9)	-	37.5	-	5	-	-
BF148012	SAM domain and HD domain, 1 (Samhd1)	-	3.1	-	2	-	-
NM_023380	SAM domain, SH3 domain and nuclear localization signals, 1 (Samsn1)	-	4.4	-	-	-	-
BC005549	SAR1 gene homolog A (Sar1a)	-	2	-	-	-	-
BI217574	Sarcosine dehydrogenase (Sardh)	-	-2.9	-	-	-	-
BC016096	Scavenger receptor class A, member 5 (putative) (Scara5)	-	3.8	-	-	-	-
BB223018	Schlafen 1 (Slfn1)	-	-	-	-	-	2.1
NM_011408	Schlafen 2 (Slfn2)	-	2.2	-	2.7	-	-

NM_011409	Schlafen 3 (Slfn3)	-	2.8	-	3.1	-	-
AF099975	Schlafen 4 (Slfn4)	-	23.7	-	10.4	-	4.3
BB134615	Schlafen 5 (Slfn5)	-	2.6	-	-	-	-
NM_022886	Sciellin (Scel)	-	3.5	-	-	-	-
BC010238	Scotin gene (Scotin)	-	2	-	-	-	-
X67702	Secretoglobin, family 1A, member 1 (uteroglobin) (Scgb1a1)	-	115.5	-	-	-	-
AF274959	Secretoglobin, family 3A, member 2 (Scgb3a2)	-	21	-2.5	-	-	-
NM_011414	Secretory leukocyte peptidase inhibitor (Slpi)	-	3.5	2.3	-	-	-
M36005	Selectin, lymphocyte (Sell)	-	2.5	-	-	-	-
BB224329	Selectin, platelet (Selp)	-	-	-	-	-	2.7
M72332	Selectin, platelet (Selp)	-	2.2	-	-	-	-
AW122690	Selenoprotein K (Selk)	-	2.2	-	-	-	-
AA144045	Sema domain, immunoglobulin domain (Ig), and GPI membrane anchor, (semaphorin) 7A (Sema7a)	-	2.7	-	-	-	-
AF080090	Sema domain, immunoglobulin domain (Ig), short basic domain, secreted, (semaphorin) 3 F (Sema3f)	-	2.4	-	-	-	-
AK004119	Sema domain, immunoglobulin domain (Ig), short basic domain, secreted, (semaphorin) 3C (Sema3c)	-	7.7	-	-	-	-
NM_011348	Sema domain, immunoglobulin domain (Ig), short basic domain, secreted, (semaphorin) 3E (Sema3e)	-	3.9	-	-	-	-
NM_011976	Sema domain, immunoglobulin domain (Ig), transmembrane domain (TM) and short cytoplasmic domain, (semaphorin) 4G (Sema4g)	-	-3.7	-	-	-	-
AV375653	Sema domain, seven thrombospondin repeats (type 1 and type 1-like), transmembrane domain (TM) and short cytoplasmic domain, (semaphorin) 5A (Sema5a)	-	5.2	-	-	-	-
NM_009154	Semaphorin 5A (Sema5a)	-	-	-	2.3	-	-
AK007282	Seminal vesicle secretion 5 (Svs5)	-	-3.5	-	-	-	-
NM_017380	Septin 9 (Sept9)	-	-2.1	-	-	-	-
BC018416	Serine (or cysteine) peptidase inhibitor, clade A (alpha-1 antiproteinase, antitrypsin), member 10 (Serpina10)	-	2.4	-	-	-	-
BB222737	Serine (or cysteine) peptidase inhibitor, clade A (alpha-1 antiproteinase, antitrypsin), member 7 (Serpina7)	-	8.4	-	-	-	-
AK009343	Serine (or cysteine) peptidase inhibitor, clade A (alpha-1 antiproteinase, antitrypsin), member 9 (Serpina9)	-	-2.8	-	-	-	-
NM_008458	Serine (or cysteine) peptidase inhibitor, clade A, member 3C (Serpina3c)	-	4	-	2.9	-	-
BC002065	Serine (or cysteine) peptidase inhibitor, clade A, member 3G (Serpina3g)	-	20.9	-	11.6	-	3.7

BC011158	Serine (or cysteine) peptidase inhibitor, clade A, member 3M (Serpina3m)	-	-	-	4.2	-	-
NM_009252	Serine (or cysteine) peptidase inhibitor, clade A, member 3N (Serpina3n)	-	3.6	6.4	11.9	-	4
NM_007618	Serine (or cysteine) peptidase inhibitor, clade A, member 6 (Serpina6)	-	-6.3	-	-	-	-
NM_011454	Serine (or cysteine) peptidase inhibitor, clade B, member 6b (Serpinb6b)	-	9.3	-	-	-	-
NM_009256	Serine (or cysteine) peptidase inhibitor, clade B, member 9 (Serpinb9)	-	2.1	-	-	-	-
NM_008223	Serine (or cysteine) peptidase inhibitor, clade D, member 1 (Serpind1)	-	-2.1	-	4	-	-
NM_009255	Serine (or cysteine) peptidase inhibitor, clade E, member 2 (Serpine2)	-	6.5	-	-	-	-
AK012411	Serine (or cysteine) peptidase inhibitor, clade F, member 1 (Serpinf1)	-	-2	-	-	-	-
BI220012	Serine (or cysteine) peptidase inhibitor, clade H, member 1 (Serpinh1)	-	2.5	-	-	-	-
NM_009171	Serine hydroxymethyl transferase 1 (soluble) (Shmt1)	-	-2.9	-	-	-	-
BM222403	Serine hydroxymethyl transferase 2 (mitochondrial) (Shmt2)	-	-2.1	-	-	-	-
BM239368	Serine incorporator 3 (Serinc3)	-	2.8	-	-	-	-
BC003227	Serine palmitoyltransferase, long chain base subunit 2 (Sptlc2)	-	2.1	-	-	-	-
NM_016907	Serine protease inhibitor, Kunitz type 1 (Spint1)	-	2.1	-	-	-	-
BQ173986	Serine racemase (Srr)	-	-2	-	-	-	-
AV173139	Serine/threonine kinase 17b (apoptosis-inducing) (Stk17b)	-	2.2	-	-	-	-
NM_011314	Serum amyloid A 1 (Saa1)	5.2	43	-	5.2	-	-
NM_011315	Serum amyloid A 3 (Saa3)	-	53	13.7	29.4	-	6.7
NM_011316	Serum amyloid A 4 (Saa4)	-	2	-	-	-	-
NM_011318	Serum amyloid P-component (Apcs)	-	2.7	-	-	-	-
NM_013731	Serum/glucocorticoid regulated kinase 2 (Sgk2)	-	-4.9	-	-	-	-
AV016566	Sestrin 1 (Sesn1)	-	-	-	-	2.4	-
BC023119	SET and MYND domain containing 2 (Smyd2)	-	-2.2	-	-	-	-
NM_021423	SH3/ankyrin domain gene 3 (Shank3)	-	2	-	-	-	-
NM_015756	Shroom family member 3 (Shroom3)	-	2.1	-	-	-	-
BG066686	Sideroflexin 2 (Sfxn2)	-	-2	-	-	-	-
BB379739	Sideroflexin 5 (Sfxn5)	-	-2.3	-	-	-	-
W08076	Signal recognition particle 19 (Srp19)	-	2.2	-	-	-	-
BC005543	Signal recognition particle 54 (Srp54)	-	2.2	-	-	-	-
NM_009275	Signal recognition particle receptor, B subunit (Sprb)	-	2	-	-	-	-
AW214029	Signal transducer and activator of transcription 1 (Stat1)	-	3.5	-	2.7	-	-
AF088862	Signal transducer and activator of transcription 2 (Stat2)	-	2.3	-	-	-	-

AK004083	Signal transducer and activator of transcription 3 (Stat3)	-	2.1	-	-	-	-
BC024319	Signal transducer and activator of transcription 5B (Stat5b)	-	-2.2	-	-	-	-
AV245241	similar to EH-domain containing 2 (Ehd2)	-	2	-	-	-	-
BI666155	Similar to guanine nucleotide binding protein-like 2 (nucleolar) (Gnl2)	-	2.3	-	-	-	-
W91024	Similar to histone 2a (H2A)	-	2	-	-	-	-
NM_008329	Similar to Interferon-activatable protein 204 (Ifi-204)	-	4.3	-	-	-	3.3
AY052560	Similar to RNA-binding motif protein 3 (Rbm3)	-	2.8	-	-	-	-
AI662854	Similar to SIRP beta 1 isoform 2	-	-	-	-	-	2
BG083485	Similar to Transcription factor SOX-4	-	-	-	-3.1	-	-
AF170906	Single-stranded DNA binding protein 3 (Ssbp3)	-	-2.1	-	-	-	-
BC026403	Sirtuin 7 (silent mating type information regulation 2, homolog) 7 (Sirt7)	-	-2	-	-	-	-
BB704967	SLAIN motif family, member 1 (Slain1)	-	-	-	-	2.1	-
BB493265	Small nucleolar RNA, C/D box 22 (Snord22)	-	2.4	-	-	-	-
BG807990	Small nucleolar RNA, H/ACA box 65 (Snora65)	-	2.4	-	-	-	-
AV299469	Smith-Magenis syndrome chromosome region, candidate 7 homolog (Smcr7)	-	-2.1	-	-	-	-
NM_011326	Sodium channel, nonvoltage-gated 1 gamma (Scnn1g)	-	2.9	-	-	-	-
BB096886	Sodium channel, voltage-gated, type III, alpha (Scn3a)	-	-	-2.7	-2.7	-	-
BE951842	Sodium channel, voltage-gated, type III, beta (Scn3b)	-	3.2	-	-	-	-
BB452990	Sodium channel, voltage-gated, type VII, alpha (Scn7a)	-	4	-	-2.3	-	-
NM_009201	Solute carrier family 1 (neutral amino acid transporter), member 5 (Slc1a5)	-	2.1	-	-	-	-
AV244484	Solute carrier family 10 (sodium/bile acid cotransporter family), member 6 (Slc10a6)	-	4.6	-	2.2	-	-
BG069505	Solute carrier family 12, member 2 (Slc12a2)	-	2.1	-	-	-	-
BE979238	Solute carrier family 13 (sodium-dependent citrate transporter), member 5 (Slc13a5)	-	3.8	-	-	-	-
NM_023044	Solute carrier family 15, member 3 (Slc15a3)	-	3.3	-	-	-	-
AW105741	Solute carrier family 16 (monocarboxylic acid transporters), member 2 (Slc16a2)	-	-2.5	-	-	-	-
BB449198	Solute carrier family 19 (thiamine transporter), member 2 (Slc19a2)	-	-4.2	-	-	-	-
AB008453	Solute carrier family 2 (facilitated glucose transporter), member 4 (Slc2a4)	-	-	-	-	-2.3	-
BB148652	Solute carrier family 2 (facilitated glucose transporter), member 9 (Slc2a9)	-	-2.8	-	-	-	-
BC026598	Solute carrier family 22 (organic anion transporter), member 7 (Slc22a7)	-	-2.6	-	-	-	-

BF577497	Solute carrier family 22 (organic cation transporter), member 18 (Slc22a18)	-	-2.1	-	-	-	-
NM_008767	Solute carrier family 22 (organic cation transporter), member 18 (Slc22a18)	-	-2.2	-	-	-	-
AV327862	Solute carrier family 25 (mitochondrial carrier, adenine nucleotide translocator), member 4 (Slc25a4)	-	5.1	-	-	-	-
BC003222	Solute carrier family 25 (mitochondrial carrier, dicarboxylate transporter), member 10 (Slc25a10)	-	-2.3	-	-	-	-
NM_025877	Solute carrier family 25 (mitochondrial carrier; phosphate carrier), member 23 (Slc25a23)	-	-2.9	-	-	-	-
BB503267	Solute carrier family 25, member 36 (Slc25a36)	-	2.4	-	-	-	-
BC025937	Solute carrier family 25, member 42 (Slc25a42)	-	-2.6	-	-	-	-
BC022130	Solute carrier family 26 (sulfate transporter), member 1 (Slc26a1)	-	-3.4	-	-	-	-
NM_011867	Solute carrier family 26, member 4 (Slc26a4)	-	-	2.8	-	-	-
AF305501	Solute carrier family 29 (nucleoside transporters), member 1 (Slc29a1)	-	-3.3	-	-	-	-
NM_022885	Solute carrier family 30 (zinc transporter), member 5 (Slc30a5)	-	2.2	-	-	-	-
BB736474	Solute carrier family 30, member 10 (Slc30a10)	-2	-	-	-	-	-
NM_011402	Solute carrier family 34 (sodium phosphate), member 2 (Slc34a2)	-	9	-	-	-	-
AV376428	Solute carrier family 37 (glycerol-3-phosphate transporter), member 1 (Slc37a1)	-	2.6	-	-	-	-
BM248527	Solute carrier family 38, member 2 (Slc38a2)	-	2.8	-	-	-	-
BB825002	Solute carrier family 39 (metal ion transporter), member 6 (Slc39a6)	-	2	-	-	-	-
NM_026228	Solute carrier family 39 (metal ion transporter), member 8 (Slc39a8)	-	2.6	-	-	-	-
BM250411	Solute carrier family 39 (zinc transporter), member 10 (Slc39a10)	-	3.1	-	-	-	-
BB399837	Solute carrier family 39 (zinc transporter), member 14 (Slc39a14)	-	2.9	-	2.4	-	-
BE655147	Solute carrier family 4 (anion exchanger), member 4 (Slc4a4)	-2.3	-	-	-	-	-
BF134253	Solute carrier family 41, member 1 (Slc41a1)	-	3.2	-	-	-	-
BC026874	Solute carrier family 41, member 2 (Slc41a2)	-	9.9	-	-	-	-
NM_021398	Solute carrier family 43, member 3 (Slc43a3)	-	3	-	-	-	-
BB145101	Solute carrier family 44, member 2 (Slc44a2)	-	3.4	-	-	-	-
AF320226	Solute carrier family 6 (neurotransmitter transporter), member 14 (Slc6a14)	-	14.1	-	-	-	-
NM_133661	Solute carrier family 6 (neurotransmitter transporter, betaine/GABA), member 12 (Slc6a12)	-	-2.1	-	-	-	-

BC023117	Solute carrier family 6 (neurotransmitter transporter, GABA), member 13 (Slc6a13)	-	-2.4	-	-	-	-
BB057781	Solute carrier family 6 (neurotransmitter transporter, noradrenalin), member 2 (Slc6a2)	-	2	-	-	-	-
BB453858	Solute carrier family 7 (cationic amino acid transporter, y+ system), member 11 (Slc7a11)	-	-	2.2	-	-	-
NM_030687	Solute carrier organic anion transporter family, member 1a4 (Slco1a4)	-	-2.7	-	-	-	-
BB553107	Solute carrier organic anion transporter family, member 2b1 (Slco2b1)	-	-2.2	-	-	-	-
NM_023908	Solute carrier organic anion transporter family, member 3a1 (Slco3a1)	-	2.7	-	-	-	-
AV024403	Solute carrier organic anion transporter family, member 4C1 (Slco4c1)	-	4.1	-	-	-	-
BI143942	Sorbitol dehydrogenase (Sord)	-	-4.7	-	-	-	-
BI648081	Sortilin-related receptor, LDLR class A repeats-containing (Sorl1)	-	2.8	-	-	2	-
AK010399	Sorting nexin 10 (Snx10)	-	6.8	-	-	-	-
BM117672	Sparc/osteonectin, cwcv and kazal-like domains proteoglycan 2 (Spock2)	-	3.2	-	-	-	-
NM_010097	SPARC-like 1 (mast9, hevin) (Sparcl1)	-	2.8	-	-	-	-
BQ174069	Spectrin beta 2 (Spnb2)	-	2.2	-	-	-	-
AF068749	Sphingosine kinase 1 (Sphk1)	-	3.1	-	-	-	-
BC023083	SplA/ryanodine receptor domain and SOCS box containing 4 (Spsb4)	-	-3.2	-	-	-	-
U36776	Spleen tyrosine kinase (Syk)	-	2.1	-	-	-	-
BC027391	Splicing factor, arginine/serine-rich 7 (Sfrs7)	-	2	-	-	-	-
BC020531	Spondin 1, (f-spondin) extracellular matrix protein (Spon1)	-	8.7	-	-	-	-
BB529691	Sprouty homolog 2 (Spry2)	-	2.1	-	-	-	-
BM242524	SPT2, Suppressor of Ty, domain containing 1 (Spty2d1)	-	3	-	-	-	-
NM_009270	Squalene epoxidase (Sqle)	-	2.1	-	-	-	-
AV247013	Src-like adaptor (Sla)	-	-	-	-	2	-
AK004781	SRY-box containing gene 17 (Sox17)	-	2.2	-	-	-	-
AI428101	SRY-box containing gene 4 (Sox4)	-	-	-	-2.2	-	-
NM_018784	ST3 beta-galactoside alpha-2,3-sialyltransferase 6 (St3gal3)	-	2.1	-	-	-	-
NM_054098	STEAP family member 4 (Steap4)	-	7.5	-	3.7	-	6.1
BG060909	Stearoyl-Coenzyme A desaturase 2 (Scd2)	-	2.9	-	-	-	-
BB667930	Stefin A2 like 1 (Stfa2l1)	-	2.2	-	3.4	-	-
BB145092	Sterile alpha motif domain containing 9-like (Samd9l)	-	2.5	-	-	-	-
AV003635	Steroid 5 alpha-reductase 1 (Srd5a1)	-	-2.3	-	-	-	-
BG064396	Sterol O-acyltransferase 1 (Soat1)	-	3	-	-	2.4	-
AI326423	Sterol regulatory element binding factor 1 (Srebf1)	-	-4	-	-	-	-
AB016248	Sterol-C5-desaturase (fungal ERG3, delta-5-desaturase) homolog (Sc5d)	-	-2.5	-	-	-	-

BF148215	Strawberry notch homolog 2 (Sbno2)	-	2.8	-	-	-	-
BE533039	Stress 70 protein chaperone, microsome-associated, human homolog (Stch)	-	2.7	-	-	-	-
BF148627	Striatin, calmodulin binding protein 3 (Strn3)	-	2	-	-	-	-
NM_022324	Stromal cell-derived factor 2-like 1 (Sdf2l1)	-	-	2	-	-	-
NM_032400	Succinate receptor 1 (Sucnr1)	-	-7.9	-	-	-	-
BM210600	Sulfiredoxin 1 homolog (Srxn1)	-	-	2.7	-	-	-
BC027197	Sulfite oxidase (Suox)	-	-2.5	-	-	-	-
NM_023135	Sulfotransferase family 1E, member 1 (Sult1e1)	2.6	-	-	-	-	-
NM_026935	Sulfotransferase family, cytosolic, 1C, member 2 (Sult1c2)	-	-3.6	-	-	-	-
BQ174944	Superoxide dismutase 2, mitochondrial (Sod2)	-	2.3	-	-	-	-
AB000710	Suppressor of cytokine signaling 1 (Socs1)	-	8	-	-	-	-
BB241535	Suppressor of cytokine signaling 3 (Socs3)	-	15	-	4	-	5.3
NM_023134	Surfactant associated protein A1 (Sftpa1)	-	67.6	-	-	-	-
AV025094	Surfactant associated protein B (Sftpb)	-	15.6	-	-	-	-
NM_011359	Surfactant associated protein C (Sftpc)	-	119.4	-	-	-	-
BC003705	Surfactant associated protein D (Sftpd)	-	34.3	-	-	-	-
BI788645	Syndecan 1 (Sdc1)	-	-2.2	-	-	-	-
BC005679	Syndecan 4 (Sdc4)	-	-	-	-	-	3.9
AV227603	Syndecan binding protein (Sdcbp)	-	2.1	-	-	-	-
AK017897	Syntaxin 11 (Stx11)	-	2.1	-	-	-	-
AK016910	Syntaxin 18 (Stx18)	-	2	-	-	-	-
AF326545	Syntaxin binding protein 1 (Stxbp1)	-	2	-	-	-	-
BI646094	Syntrophin, basic 2 (Sntb2)	-	2.2	-	-	-	-
NM_133742	Syntrophin, gamma 2 (Sntg2)	-	-2.7	-	-	-	-
AW555571	TAF9B RNA polymerase II, TATA box binding protein (TBP)-associated factor (Taf9b)	-	2	-	-	-	-
NM_019786	TANK-binding kinase 1 (Tbk1)	-	2.4	-	-	-	-
BG070463	Taxilin alpha (Txlna)	-	2.1	-	-	-	-
AA270038	TBC1 domain family, member 24 (Tbc1d24)	-	2.2	-	-	-	-
AK014817	TBC1 domain family, member 8B (Tbc1d8b)	-	2	-	-	-	-
AV226212	T-box 4 (Tbx4)	-	2.9	-	-	-	-
BB707122	TCDD-inducible poly(ADP-ribose) polymerase (Tiparp)	-	3	-	-	-	2.1
BC028829	T-cell immunoglobulin and mucin domain containing 2 (Timd2)	-	-2.6	-	-	-	-
NM_011579	T-cell specific GTPase (Tgtp)	-	11.1	-	7.2	-	4.1
NM_027884	Tensin 1 (Tns1)	-	2.7	-	-	-	-
BC010465	Testis derived transcript (Tes)	-	2.5	-	-	-	-
BB807707	Tetraspanin 13 (Tspan13)	-	5.6	-	-	-	-
BC007185	Tetraspanin 2 (Tspan2)	-	3	-	-	-	-
BC025461	Tetraspanin 8 (Tspan8)	-	12.8	-	-	-	-
BG073155	THAP domain containing, apoptosis associated protein 2 (Thap2)	-	2.2	-	-	-	-
NM_025790	Thioesterase superfamily member 2 (Them2)	-	-2.5	-	-	-	-
BB744467	Thiopurine methyltransferase (Tpmt)	-	-2.2	-	-	-	-
BC025604	Threonine synthase-like 2 (Thnsl2)	-2.1	-3	-	-	-	-

BQ176328	Threonyl-tRNA synthetase-like 2 (Tarsl2)	-	-2	-	-	-	-
NM_009378	Thrombomodulin (Thbd)	-	9.5	-	-	-	-
AI385532	Thrombospondin 1 (Thbs1)	-	4.1	-	-	-	-
NM_009387	Thymidine kinase 1 (Tk1)	-	-3.1	-	-	-	-
BB521695	Thymoma viral proto-oncogene 3 (Akt3)	-	2.7	-	-	-	-
BB096368	Thymosin, beta 10 (Tmsb10)	-	2.9	-	-	-	-
NM_021278	Thymosin, beta 4, X chromosome (Tmsb4x)	-	2.2	-	-	-	-
NM_009381	Thyroid hormone responsive SPOT14 homolog (Thrsp)	3	-	-	-	-	-
BC008107	Tissue inhibitor of metalloproteinase 1 (Timp1)	-	5.6	3.2	7.1	-	-
BI111620	Tissue inhibitor of metalloproteinase 3 (Timp3)	-	5.9	-	-	-	-
BI788452	Tissue inhibitor of metalloproteinase 4 (Timp4)	-	-	2.3	2.1	-	-
BB718785	TM2 domain containing 2 (Tm2d2)	-	-2.4	-	-	-	-
AJ242777	TNFAIP3 interacting protein 1 (Tnip1)	-	2.3	-	-	-	-
NM_139064	TNFAIP3 interacting protein 2 (Tnip2)	-	2.2	-	-	-	-
BI655907	Toll-like receptor 13 (Tlr13)	-	-	-	2	-	-
NM_011905	Toll-like receptor 2 (TLR2)	-	9.2	-	-	-	-
AF185285	Toll-like receptor 4 (TLR4)	-	2.6	-	-	-	-
BB277065	Traf2 binding protein (T2bp)	-	14.4	-	2.7	-	-
BB795263	Trafficking protein, kinesin binding 2 (Trak2)	-	2.5	-	-	-	-
BC010807	Transcription elongation factor A (SII), 3 (Tcea3)	-	-5.2	-	-	-	-
AI987804	Transcription factor 2 (Tcf2)	-	-2.2	-	-	-	-
BB364520	Transcription factor 4 (Tcf4)	-	2.2	-	-	-	-
AV174616	Transducer of ERBB2, 2 (Tob2)	-	2	-	-	-	-
NM_009389	Transducin-like enhancer of split 3, homolog of Drosophila E(spl) (Tle3)	-	2	-	-	-	-
BM237750	Transferrin (Trf)	-	3.6	-	-	-	-
AW495711	Transformation related protein 53 inducible nuclear protein 1 (Trp53inp1)	-	2	-	-	-	-
BG793483	Transforming growth factor, beta receptor II (Tgfbr2)	-	2.3	-	-	-	-
BC004057	Transforming, acidic coiled-coil containing protein 2 (Tacc2)	-	6.8	-	-	-	-
BB550124	Transglutaminase 2, C polypeptide (Tgm2)	-	2.5	-	-	-	2.1
BQ177170	Transmembrane 4 superfamily member 1 (Tm4sf1)	-	2.7	-	-	-	-
BC024498	Transmembrane 6 superfamily member 2 (Tm6sf2)	-	-2.2	-	-	-	-
AK004359	Transmembrane and coiled-coil domains 2 (Tmcc2)	-	5	-	-	-	-
BB667469	Transmembrane channel-like gene family 4 (Tmc4)	-	2.8	-	-	-	-
NM_025458	Transmembrane emp24 protein transport domain containing 6 (Tmed6)	-	-2.2	-	-	-	-
NM_026433	Transmembrane protein 100 (Tmem100)	-	7.5	-	-	-	-
AW551717	Transmembrane protein 141 (Tmem141)	-	-2	-	-	-	-
BC014685	Transmembrane protein 150 (Tmem150)	-	-3	-	-	-	-

BC006049	Transmembrane protein 176A (Tmem176a)	-	2.4	-	-	-	-
BB400326	Transmembrane protein 180 (Tmem180)	-	-3	-	-	-	-
BC019745	Transmembrane protein 2 (Tmem2)	-	2.3	-	-	-	-
AW985925	Transmembrane protein 23 (Tmem23)	-	5.3	-	-	-	-
AK002841	Transmembrane protein 25 (Tmem25)	-	-2.3	-	-	-	-
BB469903	Transmembrane protein 26 (Tmem26)	-	-	-	-	2.3	-
AK017817	Transmembrane protein 39a (Tmem35a)	-	2.2	-	-	-	-
BC004841	Transmembrane protein 50B (Tmem50b)	-	2.6	-	-	-	-
BG070008	Transmembrane protein 87B (Tmem87b)	-	3.6	-	-	-	-
AK004939	Transmembrane serine protease 6 (Tmprss6)	-	-2.2	-	-	-	-
AW048052	Transporter 1, ATP-binding cassette, sub-family B (MDR/TAP) (Tap1)	-	3.5	-	3.5	-	-
BE691515	Transporter 2, ATP-binding cassette, sub-family B (MDR/TAP) (Tap2)	-	2.1	-	-	-	-
BB354684	Tribbles homolog 2 (Trib2)	-	2.8	-	-	-	-
NM_021406	Triggering receptor expressed on myeloid cells 1 (Trem1)	-	2.3	-	2.5	-	-
BC010580	Tripartite motif protein 21 (Trim21)	-	2.3	-	-	-	-
AF220019	Tripartite motif protein 3 (Trim3)	-	2.7	-	-	-	-
BM240719	Tripartite motif protein 30 (Trim30)	-	3.1	-	-	-	2.1
BB825733	Tripartite motif protein 7 (Trim7)	-	-2.7	-	-	-	-
BB484264	Tripeptidyl peptidase II (Tpp2)	-	2.6	-	-	-	-
AV122663	Tropomyosin 4 (Tpm4)	-	2.8	-	-	-	-
NM_011619	Troponin T2, cardiac (Tnnt2)	-	6.1	-	-	-	-
AI528863	Tryptophanyl-tRNA synthetase (Wars)	-	4.7	-	7	-	-
BB045401	TSC22 domain family 2 (Tsc22d2)	-	3.1	-	-	-	-
NM_010286	TSC22 domain family 3 (Tsc22d3)	-	-	3.4	3.5	-	-
BF228294	TSR1, 20S rRNA accumulation, homolog (Tsr1)	-	2.5	-	-	-	-
BB795572	Tubulin tyrosine ligase-like family, member 7 (Ttll7)	-	2.4	-	-	-	-
NM_011653	Tubulin, alpha 1 (Tuba1)	-	2.5	-	-	-	-
AW491660	Tubulin, alpha 4 (Tuba4)	-	-2.1	-	-	-	-
AA986082	Tubulin, beta 2b (Tubb2b)	-	-	-	-	2.2	-
BB187486	Tumor necrosis factor (ligand) superfamily, member 10 (Tnfsf10)	-	2.3	-	-	-	-
NM_009396	Tumor necrosis factor, alpha-induced protein 2 (Tnfaip2)	-	2	-	2.9	-	-
BM241351	Tumor necrosis factor, alpha-induced protein 3 (Tnfaip3)	-	15.4	-	2.7	-	-
NM_134131	Tumor necrosis factor, alpha-induced protein 8 (Tnfaip8)	-	2	-	-	-	-
BC002036	Tumor protein D52 (Tpd52)	-	2	-	-	-	-
BC005618	Tumor-associated calcium signal transducer 1 (Tacstd1)	-	2.1	-	-	-	-
AV241768	Tumor-associated calcium signal transducer 2 (Tacstd2)	-	2.3	-	-	-	-
BI904914	Two pore channel 1 (Tpcn1)	-	-2	-	-	-	-
NM_011662	TYRO protein tyrosine kinase binding protein (Tyrobp)	-	2.3	-	-	-	-

NM_138721	U7 snRNP-specific Sm-like protein LSM10 (Lsm10)	-	-2.6	-	-	-	-
AK013437	Ubiquitin specific peptidase 16 (Usp16)	-	2	-	-	-	-
BG076275	Ubiquitin specific peptidase 53 (Usp53)	-	2.8	-	-	-	-
AI553394	Ubiquitin specific protease 2 (Usp2)	3	-	-	-	-	-
BB795733	UDP-Gal:betaGlcNAc beta 1,3-galactosyltransferase, polypeptide 1 (B3galt1)	-	19.8	-	-	-	-
BF682223	UDP-glucose ceramide glucosyltransferase (Ugcg)	-	2.4	-	-	-	-
AI788759	UDP-glucose pyrophosphorylase 2 (Ugp2)	-	-3.7	-	-	-	-
AV302406	UDP-N-acetyl-alpha-D-galactosamine: polypeptide N-acetylgalactosaminyltransferase 7 (Galnt7)	-	2.3	-	-	-	-
AV238718	UDP-N-acetyl-alpha-D-galactosamine:polypeptide N-acetylgalactosaminyltransferase-like 4 (Galntl4)	-	3.4	-	-	-	-
AV220213	Unc-45 homolog B (Unc45b)	-	2	-	-	-	-
BC012697	Uncoupling protein 2 (mitochondrial, proton carrier) (Ucp2)	-	2.9	-	-	-	-
NM_009477	Uridine phosphorylase 1 (Upp1)	-	3.2	-	2.8	-	-
BQ084786	Uroplakin 3B (Upk3b)	-	-	-	-	-2.9	-
AI788797	Utrophin (Utrn)	-	-	-	-2.2	-	-
BB250384	Vascular cell adhesion molecule 1 (Vcam1)	-	3.6	-	-	-	-
BC015289	Vasodilator-stimulated phosphoprotein (Vasp)	-	2.3	-	-	-	-
BB530515	Vasorin (Vasn)	-	2.4	-	-	-	-
BB552111	Vesicle-associated membrane protein 3 (Vamp3)	-	-	-	-	2.3	-
BM119387	Villin 2 (Vil2)	-	4.8	-	-	-	-
AV147875	Vimentin (Vim)	-	5	-	-	-	-
BC022952	v-maf musculoaponeurotic fibrosarcoma oncogene family, protein F (MafF)	-	2.8	2.1	2.4	-	-
BB212004	Von Willebrand factor C and EGF domains (Vwce)	-	-2.3	-	-	-	-
BB667216	Von Willebrand factor homolog (Vwf)	-	4.1	-	-	-	-
AF334269	WAP four-disulfide core domain 2 (Wfdc2)	-	5.3	-	-	-	-
C76969	WAS/WASL interacting protein family, member 1 (Wipf1)	-	2.2	-	-	-	-
NM_009516	Wee 1 homolog (Wee1)	2	-	-	-	-	-
BC007478	WW domain binding protein 5 (Wbp5)	-	2.3	-	-	-	-
BC014727	WW domain containing transcription regulator 1 (Wwtr1)	-	2	-	-	-	-
AV286265	Xanthine dehydrogenase (Xdh)	-	3.5	-	3.5	-	2.8
NM_013842	X-box binding protein 1 (Xbp1)	-	2.2	-	-	-	-
NM_021365	X-linked lymphocyte-regulated 4B (Xlr4b)	-	2.6	-	-	-	-
BC003317	Yip1 domain family, member 5 (Yipf5)	-	2.2	-	-	-	-
BB201861	YOD1 OTU deubiquitinating enzyme 1 homologue (Yod1)	-	2.3	-	-	-	-
NM_021394	Z-DNA binding protein 1 (Zbp1)	-	2.5	-	2.6	-	2.5

BQ174973	Zinc finger and BTB domain containing 16 (Zbtb16)	-	3.6	-	5.9	-	-
BC006817	Zinc finger CCCH type containing 12A (Zc3h12a)	-	4.3	-	-	-	-
AW556219	Zinc finger CCCH type containing 7 A (Zc3h7a)	-	2.3	-	-	-	-
BG075140	Zinc finger protein 131 (Zfp131)	-	2.1	-	-	-	-
X14678	Zinc finger protein 36 (Zfp36)	-	2.5	-	-	-	-
M58566	Zinc finger protein 36, C3H type-like 1 (Zfp36l1)	-2	-	-	-	-	-
BE628275	Zinc finger protein 467 (Zfp467)	-	-2.3	-	-	-	-
BB176857	Zinc finger protein 53 (Zfp53)	-	-	-	-	2.5	-
BB008902	Zinc finger protein 9 (Zfp9)	-	2.4	-	-	-	-
NM_134007	Zinc finger, CDGSH-type domain 1 (Zcd1)	-	-2.6	-	-	-	-
BC019521	Zinc finger, FYVE domain containing 21 (Zfyve21)	-	-2.5	-	-	-	-
BB561053	Zinc finger, MYND domain containing 11 (Zmynd11)	-	-	-	-	2	-

Fold-changes (FC) shown were statistically significant (i.e., Benjamini and Hochberg corrected p value <0.05 and absolute consistency across all replicate experiments). A negative sign before values indicates down-regulation. A dash “-“ indicates no statistical difference was observed.

Supplementary Table III: KEGG pathways most significantly associated with genes altered in the livers of WT *Y. pesits*-infected mice at 48 hr post-infection.

KEGG Pathway	Number of Genes				Z scores	
	List	*Up*	*Down*	*Array*	*Up*	*Down*
Pathways Associated with Up-regulated Genes						
Cytokine-cytokine receptor interaction	49	47	2	218	7.74	-2.1
Leukocyte transendothelial migration	25	25	0	111	5.82	-2.01
Jak-STAT signaling pathway	24	21	3	135	4.33	-1.59
Toll-like receptor signaling pathway	23	23	0	84	6.72	-1.75
Cell adhesion molecules (CAMs)	23	22	1	120	4.33	-1.59
Focal adhesion	22	21	1	178	2	-2.15
Hematopoietic cell lineage	17	17	0	79	4.55	-1.69
Apoptosis	16	16	0	81	4.02	-1.71
Natural killer cell mediated cytotoxicity	14	14	0	110	1.92	-2.01
T cell receptor signaling pathway	13	13	0	93	2.22	-1.84
B cell receptor signaling pathway	11	11	0	64	2.79	-1.52
Complement and coagulation cascades	11	10	1	63	2.38	-0.81
Pathways Associated with Down-regulated Genes						
Insulin signaling pathway	18	9	9	132	-0.46	2.18
Metabolism of xenotbiotics by cytochrome P450	12	0	12	52	-2.12	7.85
Glycolysis/Gluconeogenesis	11	6	5	54	0.89	2.38
ABC transporters	10	3	7	40	-0.09	4.92
Arachidnoic acid	10	3	7	65	-0.98	3.29
Tryptophan metabolism	10	3	7	70	-1.12	3.05

Pathways with Z scores values of 2 and greater are considered as significantly over-represented, and Z scores with values of -2 and lower are considered as significantly under-represented, compared to what would be expected by chance.

Supplementary Table IV. Genes statistically differentially expressed in the livers of mice infected for 12 hr with a Δ*lpp* mutant of *Y. pestis* CO92, compared to WT bacteria

GenBank ID	Gene Name	Function	WT vs Mut	C vs WT	C vs Mut
			Liver 12 h		
			FC		
BG065457	Aldolase 1, A isoform (Aldoa)	Glycolysis	3.0	-	4.0
BB449248	Apoptosis inhibitor 5 (Api5)	Transport; regulation of apoptosis	3.0	-	2.8
X17013	B subtilis lys gene for diaminopimelate decarboxylase (EC 4.1.1.20).	Amino acid metabolism	-4.3	-	-4.5
X17013	B subtilis lys gene for diaminopimelate decarboxylase (EC 4.1.1.20).	Amino acid metabolism	-6.2	-	-6.6
X17013	B subtilis lys gene for diaminopimelate decarboxylase (EC 4.1.1.20).	Amino acid metabolism	-6.7	-	-6.8
X17013	B subtilis lys gene for diaminopimelate decarboxylase (EC 4.1.1.20).	Amino acid metabolism	-7.0	-	-7.2
X17013	B subtilis lys gene for diaminopimelate decarboxylase (EC 4.1.1.20).	Amino acid metabolism	-7.5	-	-7.6
X17013	B subtilis lys gene for diaminopimelate decarboxylase (EC 4.1.1.20).	Amino acid metabolism	-10.5	-	-11.0
X04603	B. subtilis thrB and thrC genes for homoserine kinase and threonine synthase.	Amino acid metabolism	-35.0	-	-35.8
X04603	B. subtilis thrB and thrC genes for homoserine kinase and threonine synthase.	Amino acid metabolism	-36.9	-	-39.7
X04603	B. subtilis thrB and thrC genes for homoserine kinase and threonine synthase.	Amino acid metabolism	-38.7	-	-39.7
X04603	B. subtilis thrB and thrC genes for homoserine kinase and threonine synthase.	Amino acid metabolism	-50.3	-	-55.4

X04603	B. subtilis thrB and thrC genes for homoserine kinase and threonine synthase.	Amino acid metabolism	-51.9	-	-53.7
X04603	B. subtilis thrB and thrC genes for homoserine kinase and threonine synthase.	Amino acid metabolism	-64.9	-	-69.9
K01391	B.subtilis tryptophan (trp) operon, complete cds.	Amino acid metabolism	25.9	-	25.9
K01391	B.subtilis tryptophan (trp) operon, complete cds.	Amino acid metabolism	12.2	-	13.0
K01391	B.subtilis tryptophan (trp) operon, complete cds.	Amino acid metabolism	7.8	-	7.7
L38424	Bacillus subtilis dihydropicolinate reductase (jojE) gene, complete cds; poly(A) polymerase (jojI) gene, complete cds; biotin acetyl-CoA-carboxylase ligase (birA) gene, complete cds; jojC, jojD, jojF, jojG, jojH genes, complete cds s.	Transcription regulation; protein modification; amino acid biosynthesis; RNA processing; methylglyoxal biosynthesis	-40.7	-	-51.5
L38424	Bacillus subtilis dihydropicolinate reductase (jojE) gene, complete cds; poly(A) polymerase (jojI) gene, complete cds; biotin acetyl-CoA-carboxylase ligase (birA) gene, complete cds; jojC, jojD, jojF, jojG, jojH genes, complete cds s.	Transcription regulation; protein modification; amino acid biosynthesis; RNA processing; methylglyoxal biosynthesis	-71.7	-	-89.8
L38424	Bacillus subtilis dihydropicolinate reductase (jojE) gene, complete cds; poly(A) polymerase (jojI) gene, complete cds; biotin acetyl-CoA-carboxylase ligase (birA) gene, complete cds; jojC, jojD, jojF, jojG, jojH genes, complete cds s.	Transcription regulation; protein modification; amino acid biosynthesis; RNA processing; methylglyoxal biosynthesis	-95.9	-	-126.1
L38424	Bacillus subtilis dihydropicolinate reductase (jojE) gene, complete cds; poly(A) polymerase (jojI) gene, complete cds; biotin acetyl-CoA-carboxylase ligase (birA) gene, complete cds; jojC, jojD, jojF, jojG, jojH genes, complete cds s.	Transcription regulation; protein modification; amino acid biosynthesis; RNA processing; methylglyoxal biosynthesis	-97.8	-	-119.3

L38424	Bacillus subtilis dihydropicolinate reductase (jojE) gene, complete cds; poly(A) polymerase (jojI) gene, complete cds; biotin acetyl-CoA-carboxylase ligase (birA) gene, complete cds; jojC, jojD, jojF, jojG, jojH genes, complete cds s.	Transcription regulation; protein modification; amino acid biosynthesis; RNA processing; methylglyoxal biosynthesis	-116.0	-	-159.1
L38424	Bacillus subtilis dihydropicolinate reductase (jojE) gene, complete cds; poly(A) polymerase (jojI) gene, complete cds; biotin acetyl-CoA-carboxylase ligase (birA) gene, complete cds; jojC, jojD, jojF, jojG, jojH genes, complete cds s.	Transcription regulation; protein modification; amino acid biosynthesis; RNA processing; methylglyoxal biosynthesis	-167.8	-	-226.1
M24537	Bacillus subtillis sporulation protein (spoOB), GTP-binding protein (obg), phenylalanine biosynthesis associated protein (pheB), and monofunctional prephenate dehydratase (pheA) genes, complete cds.	Sporulation; response to stimulus; amino acid biosynthesis	-2.4	-	-2.4
M24537	Bacillus subtillis sporulation protein (spoOB), GTP-binding protein (obg), phenylalanine biosynthesis associated protein (pheB), and monofunctional prephenate dehydratase (pheA) genes, complete cds.	Sporulation; response to stimulus; amino acid biosynthesis	-3.3	-	-3.4
M24537	Bacillus subtillis sporulation protein (spoOB), GTP-binding protein (obg), phenylalanine biosynthesis associated protein (pheB), and monofunctional prephenate dehydratase (pheA) genes, complete cds.	Sporulation; response to stimulus; amino acid biosynthesis	-3.5	-	-3.5

M24537	Bacillus subtillis sporulation protein (spoOB), GTP-binding protein (obg), phenylalanine biosynthesis associated protein (pheB), and monofunctional prephenate dehydratase (pheA) genes, complete cds.	Sporulation; response to stimulus; amino acid biosynthesis	-3.9	-	-4.0
M24537	Bacillus subtillis sporulation protein (spoOB), GTP-binding protein (obg), phenylalanine biosynthesis associated protein (pheB), and monofunctional prephenate dehydratase (pheA) genes, complete cds.	Sporulation; response to stimulus; amino acid biosynthesis	-4.2	-	-4.1
M24537	Bacillus subtillis sporulation protein (spoOB), GTP-binding protein (obg), phenylalanine biosynthesis associated protein (pheB), and monofunctional prephenate dehydratase (pheA) genes, complete cds.	Sporulation; response to stimulus; amino acid biosynthesis	-5.2	-	-5.3
BB213876	Carbonic anhydrase 3 (Car3)	One-carbon compound metabolism	2.7	-	3.3
AK018466	cDNA	Unknown	-2.4	-	-2.0
C76675	Ribosomal protein S6 (Rps6)	Protein biosynthesis; ribosome biogenesis; glucose homeostasis	3.0	-	3.6
AK007282	Seminal vesicle secretion 5 (Svs5)	Hormone signaling	-3.1	-	-3.3

Fold-changes (FC) shown were statistically significant (i.e., Benjamini and Hochberg corrected p value <0.05 and absolute consistency across all replicate experiments). A negative sign before values indicates down-regulation. A dash "-" indicates no statistical difference was observed. C = control (uninfected) mice; WT=wild-type *Y. pestis* CO92 infected mice; Mut=Δ*lpp* mutant of *Y. pestis* CO92 infected mice.

Supplementary Table V. Genes statistically differentially expressed in the spleens of mice infected for 12 hr with a Δ*lpp* mutant of *Y. pestis* CO92, compared to WT bacteria

GenBank ID	Gene Name	Function	WT vs Mut	C vs WT	C vs Mut
Up-regulated in WT-infected Mice Compared to Uninfected Control Animals) But NOT in Mutant-infected Mice					
				FC	
BM237637	Atpase, class VI, type 11C (Atp11c)	Cation transport; metabolism	-2.4	2.1	-
AV316469	cDNA	Unknown	-3.9	4.2	-
AI098139	cDNA	Unknown	-2.7	2.3	-
BB229264	cDNA	Unknown	-2.2	2.2	-
BB357590	Cyclin D binding myb-like transcription factor 1 (Dmtf1)	Transcription regulation	-2.8	2.7	-
BB167641	Cyclin D3 (Ccnd3)	Regulation of progression through cell cycle; signal transduction; T cell proliferation; cell division	-4.2	3.8	-
BE685667	Cyclin D3 (Ccnd3)	Regulation of progression through cell cycle; signal transduction; T cell proliferation; cell division	-3.8	3.9	-
BB043576	Cyclin D3 (Ccnd3)	Regulation of progression through cell cycle; signal transduction; T cell proliferation; cell division	-3.7	3.1	-
BB723867	Dysferlin (Dysf)	Plasma membrane repair	-1.9	2.1	-
NM_133753	ERBB receptor feedback inhibitor 1 (Errfi1)	Response to stress; stress-activated protein kinase signaling pathway	-2.7	3.9	-
BG228765	Jumonji domain containing 3 (Jmjd3)	Unknown	-2.0	2.2	-
AV309996	Luc7 homolog (S. cerevisiae)-like (Luc7l)	Negative regulation of striated muscle development	-2.0	2.0	-

BC027056	Microtubule-associated protein, RP/EB family, member 2 (Mapre2)	Cellular defense response; cell cycle; mitosis; signal transduction; cell proliferation/division	-2.6	2.0	-
BB636266	Nemo like kinase (Nik)	Transcription regulation; protein amino acid phosphorylation/autophsophorylation; protein kinase cascade; negative regulation of Wnt receptor signaling pathway	-2.3	2.1	-
BB521034	Phosphomannomutase 2 (Pmm2)	Protein amino acid N-linked glycosylation; metabolism; GDP-mannose biosynthesis; mannose biosynthesis	-2.6	2.5	-
BB214530	Presenilin associated, rhomboid-like (Parl)	Serine-type endopeptidase activity	-3.5	2.5	-
NM_013743	Pyruvate dehydrogenase kinase, isoenzyme 4 (Pdk4)	Carbohydrate metabolism; glucose metabolism; acetyl-CoA biosynthesis from pyruvate	-2.7	3.1	-
BB469903	Transmembrane protein 26 (Tmem26)	Unknown	-2.3	2.3	-
Down-regulated in WT-infected Mice Compared to Uninfected Control Animals) But NOT in Mutant-infected Mice					
AF402617	BCL2-antagonist/killer 1 (Bak1)	Regulation of apoptosis; mitochondrial fusion; caspase activation via cytochrome c; response to fungus; response to mycotoxin	3.1	-2.2	-
NM_009743	Bcl2-like 1 (Bcl2l1)	Response to radiation; regulation of apoptosis	2.4	-2.3	-
NM_010438	Hexokinase 1 (Hk1)	Glycolysis	4.8	-4.8	-
BG063064	Tripartite motif protein 8 (Trim8)	Unknown	3.6	-3.0	-
AA014267	Zinc finger protein, multitype 1 (Zfpm1)	Transcription regulation; heart development	5.2	-4.3	-
Down-regulated in WT-infected Mice Compared to Uninfected Controls) But Up-regulated in Mutant-infected Mice					

AY058908	Anti-human CD37 antibody WR17 kappa light chain variable region	Immune functions	4.0	-2.4	1.6
L41881	Immunoglobulin kappa chain variable 28 (V28) (Igk-V28)	Antigen processing and presentation of peptide antigen via MHC class I	3.8	-1.8	2.1
NM_009043	Regenerating islet-derived 2 (Reg2)	Might act as an inhibitor of spontaneous calcium carbonate precipitation	3.9	-1.7	6.7
Up-regulated in Mutant-infected Mice Compared to Uninfected Control Animals) But NOT in WT-infected Mice					
BG141874	Transthyretin (Ttr)	Thyroid hormone generation; transport	3.1		3.2

Fold-changes (FC) shown were statistically significant (i.e., Benjamini and Hochberg corrected p value <0.05 and absolute consistency across all replicate experiments). A negative sign before values indicates down-regulation. A dash "-" indicates no statistical difference was observed. C = control (uninfected) mice; WT=wild-type *Y. pestis* CO92 infected mice; Mut=Δ*lpp* mutant of *Y. pestis* CO92 infected mice.

Supplementary Table VI. Genes statistically differentially expressed in the livers of mice infected for 48 hr with a Δ*lpp* mutant of *Y. pestis* CO92, compared to WT bacteria

GenBank ID	Gene Name	Function	WT vs Mut	C vs WT	C vs Mut
			FC		
Up-regulated in Both WT- and Mutant-infected Mice					
NM_009616	A disintegrin and metallopeptidase domain 19 (meltrin beta) (Adam19)	Proteolysis; membrane protein ectodomain proteolysis; integrin-mediated signaling pathway; heart development	1.9	1.5	3.0
AI649048	A kinase (PRKA) anchor protein 2 (Akap2)	Actin filament organization; transmembrane receptor protein serine/threonine kinase signaling pathway; protein localization; regulation of cell shape	1.9	6.4	12.0
BC003735	A kinase (PRKA) anchor protein 2 (Akap2)	Actin filament organization; transmembrane receptor protein serine/threonine kinase signaling pathway; protein localization; regulation of cell shape	1.9	4.7	8.7
NM_080575	Acyl-CoA synthetase short-chain family member 1 (Acss1)	Acetyl-CoA biosynthesis; metabolism	2.6	1.8	4.6
BB185861	Adaptor-related protein complex 1, sigma 2 subunit (Ap1s2)	Intracellular protein transport; receptor-mediated endocytosis	1.9	2.6	5.0
AK005223	Adaptor-related protein complex 1, sigma 2 subunit (Ap1s2)	Intracellular protein transport; receptor-mediated endocytosis	1.9	2.0	3.7
NM_024191	ADP-ribosylation factor-like 2 binding protein (Arl2bp)	Unknown	2	2.1	4.1
AK006418	ADP-ribosylation factor-like 2 binding protein (Arl2bp)	Unknown	1.8	1.8	3.3
BE570050	AHNAK nucleoprotein (desmoyokin) (Ahnak)	Intracellular signaling cascade	2.5	7.4	18.8
AV256780	Ankyrin repeat domain 44 (Ankrd44)	Transcription regulation	2.2	2.3	5.1
NM_030697	Ankyrin repeat domain 47 (Ankrd47)	Unknown	2.4	1.7	4.1
NM_007585	Annexin A2 (Anxa2)	Angiogenesis; collagen fibril organization; fibrinolysis	1.8	3.6	6.3
AW702161	Annexin A3 (Anxa3)	Signal transduction	2	6.6	13.0
BE865094	Armadillo repeat containing, X-linked 4 (Armcx4)	Proteolysis	2	2.6	5.3
AW610650	ATPase, aminophospholipid transporter (APLT), class I, type 8A, member 1 (Atp8a1)	Cation transport	2.3	5.0	11.3

BB303874	ATPase, aminophospholipid transporter (APLT), class I, type 8A, member 1 (Atp8a1)	Cation transport	1.9	4.0	7.4
BQ176779	ATPase, aminophospholipid transporter (APLT), class I, type 8A, member 1 (Atp8a1)	Cation transport	2.4	3.3	7.8
AW610650	ATPase, aminophospholipid transporter (APLT), class I, type 8A, member 1 (Atp8a1)	Cation transport	2.1	2.1	4.3
NM_009052	Brain expressed gene 1 (Bex1)	Positive regulation of neuroblast proliferation; nervous system development; negative regulation of neuron differentiation; nerve growth factor receptor signaling pathway	2.6	2.0	5.1
AK010400	Brain expressed gene 4 (Bex4)	Unknown	3.1	1.8	5.4
BB039247	CD93 antigen (Cd93)	Phagocytosis; defense response; cell adhesion; macrophage activation	2.5	4.4	11.1
AV319144	CD93 antigen (Cd93)	Phagocytosis; defense response; cell adhesion; macrophage activation	2.5	4.3	10.9
BB479310	CDC14 cell division cycle 14 homolog A (Cdc14a)	Protein amino acid dephosphorylation; cell cycle; cell division	2	1.7	3.4
BB332449	Ceruloplasmin (Cp)	Copper and iron transport and homeostasis	1.7	2.1	3.6
AA210377	Chloride intracellular channel 5 (Clic5)	Chloride transport; pregnancy	2.5	10.6	26.8
NM_013805	Claudin 5 (Cldn5)	Calcium-independent cell-cell adhesion	2.8	2.6	7.3
AV024285	Coagulation factor II (thrombin) receptor (F2r)	Regulation of progression through cell cycle; apoptosis; caspase activation; cell motility; G-protein coupled receptor protein signaling pathway; tyrosine phosphorylation of STAT protein; STAT protein nuclear translocation; blood coagulation; response to wounding; morphogenesis; positive regulation of I-kappaB kinase/NF-kappaB cascade	1.8	2.7	5.1
AW060797	Coiled-coil domain containing 85A (Ccdc85a)	Unknown	3	3.5	10.2
BB740218	Coronin, actin binding protein 1A (Coro1a)	Transport; cell motility; mitosis	1.9	2.9	5.4
BB558800	Cysteine-rich secretory protein LCCL domain containing 2 (Crispld2)	Unknown	1.9	3.5	6.6

NM_007823	Cytochrome P450, family 4, subfamily b, polypeptide 1 (Cyp4b1)	Arachidonic acid or fatty acid metabolism	2	3.2	6.5
BB795072	Dedicator of cytokinesis 9 (Dock9)	Guanyl nucleotide exchange factor activity	2	1.8	3.5
NM_007876	Dipeptidase 1 (renal) (Dpep1)	Proteolysis	2.4	3.3	8.0
NM_133838	EH-domain containing 4 (Ehd4)	Unknown	2.3	3.7	8.3
BB229377	Elastin (Eln)	Striated muscle development; respiratory gaseous exchange; circulation; cell proliferation; organ morphogenesis; regulation of actin filament polymerization; stress fiber formation	2.4	1.7	4.0
AF361882	Endothelial cell-specific adhesion molecule (Esam1)	Cell adhesion	1.9	2.3	4.3
BC008277	Endothelin receptor type A (Ednra)	Patterning of blood vessels; response to hypoxia; embryonic development; smooth muscle contraction; G-protein coupled receptor protein signaling pathway; adenylate cyclase activation; phospholipase C activation; elevation of cytosolic calcium ion concentration; heart development; respiratory gaseous exchange; blood pressure regulation; cell proliferation; neural crest cell development; glucose transport; negative regulation of cAMP biosynthesis; vasoconstriction	2	2.4	4.7
BG063148	EST	Unknown	3	5.9	17.5
BB522283	EST	Unknown	2.1	3.8	7.9
BI151331	EST	Unknown	1.9	2.5	4.7
NM_007993	Fibrillin 1 (Fbn1)	Chitin metabolism	2.1	2.6	5.4
BM936480	Flavin containing monooxygenase 2 (Fmo2)	Electron transport; oxygen and reactive oxygen species metabolism	2.6	2.8	7.0
BB437522	Forkhead box F1a (Foxf1a)	Vasculogenesis; transcription regulation; organ morphogenesis; extracellular matrix organization and biogenesis; lung and embryonic gut development; establishment of epithelial cell polarity	2.7	2.4	6.4
BB527078	Furry homolog (Fry)	Unknown	2	2.5	5.0
BB083808	G protein-coupled receptor 116 (Gpr116)	G-protein coupled receptor protein signaling pathway; neuropeptide signaling pathway	2.6	3.3	8.5

NM_008122	Gap junction membrane channel protein alpha 7 (Gja7)	Transport; muscle contraction; intercellular junction assembly; cell-cell signaling; synaptic transmission; heart development; visual perception	2	2.0	4.0
U03561	Heat shock protein 1 (Hspb1)	Protein folding; response to unfolded protein	1.8	3.6	6.3
NM_008344	Insulin-like growth factor binding protein 6 (Igfbp6)	Regulation of cell growth; signal transduction; negative regulation of cell proliferation	2.9	3.2	9.0
NM_018826	Iroquois related homeobox 5 (Irx5)	Transcription regulation; cell development	2.1	1.5	3.2
BC010337	Keratin 7 (Krt7)	Cytoskeleton organization and biogenesis	2.2	6.6	14.4
NM_008452	Kruppel-like factor 2 (lung) (Kif21a)	Transcription regulation	2.2	2.8	5.9
BG069413	Kruppel-like factor 4 (gut) (Klf4)	Transcription regulation	2.6	2.4	6.4
BG069413	Kruppel-like factor 4 (gut))Kif4)	Transcription regulation	2.9	6.7	19.2
BG069607	Kruppel-like factor 5 (Klf5)	Transcription regulation	2.3	1.8	4.1
BB187947	Leucine-rich repeat LGI family, member 3 (Lgi3)	Unknown	3.3	1.8	6.0
AV293368	Mcf.2 transforming sequence-like (Mcf2l)	Rho protein signal transduction	2.4	2.8	6.8
AK010278	Myosin, heavy polypeptide 14 (Myh14)	Regulation of cell shape; actin filament-based movement	2.3	1.6	3.6
BB481540	Myosin, heavy polypeptide 6, cardiac muscle, alpha (Myh6)	Embryonic development (sensu Mammalia); cardiac inotropy, chronotropy; striated muscle contraction; adult heart development; visceral muscle development; regulation of heart contraction; blood pressure regulation; actin filament-based movement; myofibril assembly; regulation of ATPase activity; sarcomere organization; cardiac muscle fiber development	3.1	1.8	5.5
AK020249	Naked cuticle 1 homolog (Drosophila) (Nkd1)	In the mouse, Nkd is a Dishevelled-binding protein that functions as a negative regulator of the Wnt-beta-catenin signaling pathway	2.6	1.5	4.0
BM121794	Nebulette (Nebl)	Ion transport	2.3	7.5	17.0
NM_010929	Notch gene homolog 4 (Notch4)	Patterning of blood vessels; cell fate determination; morphogenesis of a branching structure; transcription regulation; Notch signaling pathway; embryonic development; hemopoiesis; cell differentiation; negative regulation of endothelial cell	2.5	2.5	6.2

		differentiation			
BC024556	PDZ and LIM domain 2 (Pdlim2)	Unknown	2.6	3.7	9.5
BG072867	Periaxin (Prx)	Axon ensheathment	2.3	1.9	4.3
NM_008903	Phosphatidic acid phosphatase 2a (Ppap2a)	Protein amino acid dephosphorylation; diacylglycerol biosynthesis; sphingosine metabolism; ceramide metabolism; protein kinase C activation; negative regulation of cell proliferation; regulation of lipid metabolism; androgen receptor signaling pathway; phospholipid dephosphorylation; steroid hormone receptor signaling pathway	2.1	2.1	4.3
NM_011182	Pleckstrin homology, Sec7 and coiled-coil domains 3 (Pscd3)	Vesicle-mediated transport; regulation of ARF protein signal transduction; positive regulation of cell adhesion	2.1	2.1	4.5
NM_010329	Podoplanin (Pdpn)	Lymphangiogenesis; water transport; amino acid transport; cell adhesion; development; regulation of cell shape; folic acid transport; lung development; positive regulation of cell migration; tube morphogenesis; positive regulation of cell motility; cell morphogenesis	2.3	5.1	11.4
BB533892	Potassium inwardly-rectifying channel, subfamily J, member 15 (Kcnj15)	Potassium ion transport	2.6	5.3	13.8
AV366831	Procollagen, type IV, alpha 3 (Col4a3)	Phosphate transport; induction of apoptosis; caspase activation; cell adhesion; cell surface receptor linked signal transduction; cell proliferation; negative regulation of angiogenesis	2.5	3.7	9.2
BB164509	Proline rich Gla (G-carboxyglutamic acid) 3 (transmembrane) (Prrg3)	Unknown	2.1	1.9	4.0
AK009847	Protease, serine, 23 (Prss23)	Proteolysis	2.1	4.6	9.8

AV297026	Protein kinase C, mu (Prkcm)	Protein amino acid phosphorylation; intracellular signaling cascade	2.6	1.7	4.4
BB216074	Protein kinase, cAMP dependent regulatory, type II beta (Prkar2b)	Protein amino acid phosphorylation; fatty acid metabolism; intracellular signaling cascade; learning; cell proliferation; organ morphogenesis; protein amino acid phosphorylation	2.1	1.6	3.3
BB823350	Protein kinase, cGMP-dependent, type II (Prkg2)	Regulation of progression through cell cycle; protein amino acid phosphorylation; signal transduction	2.4	2.0	4.7
NM_133685	RAB31, member RAS oncogene family (Rab31)	Intracellular protein transport; small GTPase mediated signal transduction	1.6	1.6	2.7
AF146523	Receptor (calcitonin) activity modifying protein 2 (Ramp2)	Intracellular protein transport; receptor-mediated endocytosis; regulation of G-protein coupled receptor protein signaling pathway	2.3	2.7	6.1
NM_022886	Sciellin (Scel)	Epidermis development; embryonic development; keratinocyte differentiation	4.4	3.5	15.1
NM_009151	Selectin, platelet (p-selectin) ligand (Selpl)	Cell adhesion	1.7	1.6	2.7
AF080090	Sema domain, immunoglobulin domain (Ig), short basic domain, secreted, (semaphorin) 3 F (Sema3f)	Neural crest cell migration; development; negative regulation of axon extension involved in axon guidance; negative chemotaxis	2.2	2.4	5.2
BM114815	SET domain containing (lysine methyltransferase) 7 (Setd7)	Chromatin modification	1.8	1.8	3.2
NM_015756	Shroom family member 3 (Shroom3)	Cell morphogenesis; pattern specification; cellular pigment accumulation; apical protein localization	1.9	2.1	4.1
NM_011326	Sodium channel, nonvoltage-gated 1 gamma (Scnn1g)	Sodium ion transport; excretion; response to stimulus	2	2.9	5.6
BM237089	Solute carrier organic anion transporter family, member 3a1 (Slco3a1)	Ion transport	2	2.7	5.4
BB827631	Spectrin domain with coiled-coils 1 (Specc1)	Muscle contraction	1.9	1.5	3.0
BG060909	Stearoyl-Coenzyme A desaturase 2 (Scd2)	Lipid metabolism; fatty acid biosynthesis; superoxide metabolism	2.6	6.5	16.9
AW555571	TAF9B RNA polymerase II, TATA box binding protein (TBP)-associated factor (Taf9b)	Transcription initiation; regulation of transcription	2.1	2.0	4.1

NM_027884	Tensin 1 (Tns1)	Cell-substrate junction assembly; intracellular signaling cascade; cell migration	1.9	1.7	3.2
BC007185	Tetraspanin 2 (Tspan2)	Cell motility; cell adhesion; cell proliferation	2.5	3.0	7.4
BI111620	Tissue inhibitor of metalloproteinase 3 (Timp3)	Transmembrane receptor protein tyrosine kinase signaling pathway	2	6.6	12.9
BI111620	Tissue inhibitor of metalloproteinase 3 (Timp3)	Transmembrane receptor protein tyrosine kinase signaling pathway	1.9	6.6	12.5
AI639846	Transcription factor 4 (Tcf4)	Transcription regulation	1.7	1.8	3.1
BB354684	Tribbles homolog 2 (Trib2)	Protein amino acid phosphorylation; regulation of MAPK activity	2.5	2.8	7.0
BC005618	Tumor-associated calcium signal transducer 1 (Tacstd1)	Defense response	2	2.1	4.1
AV238718	UDP-N-acetyl-alpha-D-galactosamine:polypeptide N-acetylgalactosaminyltransferase-like 4 (Galntl4)	Unknown	2.1	3.4	7.2
BC012697	Uncoupling protein 2 (mitochondrial, proton carrier) (Ucp2)	Mitochondrial transport; proton transport	1.9	2.9	5.6
NM_011979	Vanin 3 (Vnn3)	Nitrogen compound metabolism	3.4	1.8	6.3
BC021433	cDNA	Unknown	1.8	25.1	44.4
BF682848	cDNA	Unknown	2.8	9.9	27.1
BM218877	cDNA	Unknown	3.2	3.7	11.9
AK004276	cDNA	Unknown	1.9	3.3	6.4
BB322233	cDNA	Unknown	2.7	3.0	8.0
BB353853	cDNA	Unknown	2.1	2.7	5.7
BB534298	cDNA	Unknown	2.7	2.6	7.0
BC022950	cDNA	Unknown	2.2	2.4	5.3
AK017241	cDNA	Unknown	2.4	1.7	4.0
Down-regulated in Both WT- and Mutant-infected Mice					
BE952632	2-4-dienoyl-Coenzyme A reductase 2, peroxisomal (decr2)	Peroxisome organization and biogenesis; metabolism	-2.9	-2.5	-7.2
AK002295	3-hydroxyanthranilate 3,4-dioxygenase (Haao)	Metabolism	-3	-1.8	-5.5
BF462185	4-aminobutyrate aminotransferase (Abat)	Gamma-aminobutyric acid metabolism; neurotransmitter catabolism; behavioral response to cocaine	-2.6	-2.3	-5.8
BC013343	4-hydroxyphenylpyruvic acid dioxygenase (Hpd)	L-phenylalanine catabolism; tyrosine catabolism; aromatic amino acid family metabolism	-2.3	-1.8	-4.1
BC010837	4-nitrophenylphosphatase domain and non-neuronal SNAP25-like protein homolog 1 (ipsnap1)	Unknown	-3.3	-2.5	-8.3

AV306253	4-nitrophenylphosphatase domain and non-neuronal SNAP25-like protein homolog 1 (ipsnap1)	Unknown	-3.1	-3.2	-10.1
BB718075	Acetyl-Coenzyme A acyltransferase 2 (mitochondrial 3-oxoacyl-Coenzyme A thiolase) (Acaa2)	Lipid metabolism; fatty acid metabolism; cholesterol biosynthesis	-2.2	-1.6	-3.5
AB078618	Acyl-CoA thioesterase 12 (Acot12)	Acetyl-CoA metabolism; lipid metabolism; fatty acid metabolism	-2.1	-3.2	-6.7
NM_016702	Alanine-glyoxylate aminotransferase (Agxt)	Protein targeting to peroxisome; glyoxylate metabolism	-2.6	-2.4	-6.1
BC013477	Alcohol dehydrogenase 1 (class I) (Adh1)	Retinoid metabolism	-4	-2.0	-7.9
BB282093	Aldehyde dehydrogenase 4 family, member A1 (Aldh4a1)	Proline metabolism	-3.7	-2.3	-8.4
BC021607	Aldo-keto reductase family 1, member C20 (Akr1c20)	Steroid biosynthesis	-6	-2.7	-15.8
NM_030611	Aldo-keto reductase family 1, member C6 (Akr1c6)	Steroid biosynthesis	-6.8	-2.0	-13.3
AI891467	Aldo-keto reductase family 1, member D1 (Akr1d1)	Electron transport; lipid metabolism; bile acid metabolism; cholesterol catabolism; digestion; C21-steroid hormone metabolism; androgen metabolism	-11.6	-3.3	-38.0
AK005066	Amidohydrolase domain containing 1 (Amdhd1)	Histidine catabolism to glutamate and formamide	-2.9	-3.5	-10.4
BB797326	Amidohydrolase domain containing 1 (Amdhd1)	Histidine catabolism to glutamate and formamide	-4.2	-2.5	-10.5
BC025904	Angiopoietin-like 6 (Angptl6)	Angiogenesis; signal transduction; development; cell differentiation	-1.7	-2.1	-3.7
D49729	Arginine vasopressin receptor 1A (Avpr1a)	Generation of precursor metabolites and energy; G-protein coupled receptor protein signaling pathway; phospholipase C activation; elevation of cytosolic calcium ion concentration; circulation	-2	-1.7	-3.4
NM_007494	Argininosuccinate synthetase 1 (Ass1)	Urea cycle; arginine biosynthesis; amino acid biosynthesis	-2.9	-1.5	-4.4
NM_023383	Arylacetamide deacetylase (esterase) (Aadac)	Metabolism	-2.5	-1.5	-3.9
AK004030	Arylformamidase (Afmid)	Tryptophan catabolism to kynurenine	-3.2	-3.7	-11.6
NM_027857	Aspartoacylase (aminoacylase) 3 (Acy3)	Metabolism	-2.2	-4.6	-10.0
NM_021022	ATP-binding cassette, sub-family B (MDR/TAP), member (Abcb11)	Transport	-6	-1.9	-11.3

NM_031884	ATP-binding cassette, sub-family G (WHITE), member 5 (Abcg5)	Transport; cholesterol absorption	-2.4	-4.2	-10.1
BC022107	Cadherin 2 (Cdh2)	Cell adhesion; calcium-dependent cell-cell adhesion; cell migration; blood vessel morphogenesis	-2	-1.9	-3.7
BC022107	Cadherin 2 (Cdh2)	Cell adhesion; calcium-dependent cell-cell adhesion; cell migration; blood vessel morphogenesis	-2.1	-1.9	-4.0
NM_007606	Carbonic anhydrase 3 (Car3)	One-carbon compound metabolism	-13.2	-2.4	-31.3
NM_007606	Carbonic anhydrase 3 (Car3)	One-carbon compound metabolism	-10.9	-4.3	-46.2
NM_021456	Carboxylesterase 1 (Ces1)	Detoxification of xenobiotics; activation of ester and amide prodrugs; resonse to toxin	-5.1	-2.3	-11.7
NM_009900	Chloride channel 2 (Clcn2)	Chloride transport	-2.4	-2.2	-5.3
NM_031164	Coagulation factor XIII, beta subunit (F13b)	Blood coagulation	-2.3	-1.6	-3.8
BB765132	Collectin sub-family member 10 (Colec10)	Phosphate transport; defense response; cell adhesion; embryonic development	-3.9	-1.7	-6.9
AF173379	Copper chaperone for superoxide dismutase (Ccs)	Protein folding; superoxide metabolism; copper ion transport; positive regulation of oxidoreductase activity	-2	-1.9	-3.8
NM_010000	Cytochrome P450, family 2, subfamily b, polypeptide 9 (Cyp2b9)	Electron transport	-7.2	-3.4	-24.5
BC025819	Cytochrome P450, family 2, subfamily c, polypeptide 44 (Cyp2c44)	Drug and sterid metabolism	-4.8	-2.7	-13.0
BC010989	Cytochrome P450, family 2, subfamily d, polypeptide 10 (Cyp2d10)	Electron transport	-1.7	-1.6	-2.8
BF783609	Cytochrome P450, family 2, subfamily j, polypeptide 5 (Cyp2j5)	Electron transport	-3.2	-2.0	-6.2
NM_134144	Cytochrome P450, family 2. subfamily c, polypeptide 37 (Cyp2c37)	Electron transport	-4.3	-1.5	-6.6
NM_007820	Cytochrome P450, family 3, subfamily a, polypeptide 16 (Cyp3a16)	Electron transport	-7.5	-1.7	-12.5
BC011228	Cytochrome P450, family 4, subfamily f, polypeptide 14 (Cyp4f14)	Arachidonic acid or fatty acid metabolism	-7.8	-4.5	-35.1
C79957	Desmoglein 2 (Dsg2)	Cell adhesion	-1.9	-1.9	-3.6
AW490997	Dihydropyrimidinase (Dpys)	Nucleobase, nucleoside, nucleotide and nucleic acid metabolism; response to toxin	-6.1	-4.1	-25.0

AK004899	Dihydropyrimidinase (Dpys)	Nucleobase, nucleoside, nucleotide and nucleic acid metabolism; response to toxin	-8.1	-3.3	-26.6
NM_023737	Enoyl-Coenzyme A, hydratase/3-hydroxyacyl Coenzyme A dehydrogenase (Ehhadh)	Generation of precursor metabolites and energy; lipid metabolism; fatty acid beta-oxidation; acyl-CoA metabolism	-4.7	-2.6	-12.0
BB667477	EST	Unknown	-4.7	-3.6	-16.8
AF055573	Fragile histidine triad gene (Fhit)	DNA replication; nucleotide metabolism	-2.1	-1.7	-3.6
BC011139	Glucokinase (Gck)	Glucose metabolism and homeostasis; glycolysis; NADP metabolism; positive regulation of insulin secretion; regulation of potassium ion transport; carbohydrate phosphorylation	-3.8	-2.4	-9.1
NM_026672	Glutathione S-transferase, mu 7 (Gstm7)	Metabolism	-2.6	-2.4	-6.0
NM_008156	Glycosylphosphatidylinositol specific phospholipase D1 (Gpld1)	GPI anchor release	-2.2	-2.6	-5.9
AF015887	Guanidinoacetate methyltransferase (Gamt)	Creatine biosynthesis; muscle contraction; spermatogenesis; organ morphogenesis; regulation of body size	-6.3	-4.0	-25.2
BC019856	Gulonolactone (L-) oxidase (Gulo)	Electron transport; L-ascorbic acid biosynthesis	-3.4	-2.8	-9.4
NM_019545	Hydroxyacid oxidase (glycolate oxidase) 3 (Hao3)	Fatty acid alpha-oxidation; electron transport; protein targeting; fatty acid metabolism	-17.6	-10.0	-175.3
BC026555	Kallikrein B, plasma 1 (Klkb1)	Proteolysis; inflammatory response; blood coagulation; fibrinolysis	-3.7	-4.2	-15.4
NM_029550	Kidney expressed gene 1 (Keg1)	Regulation of progression through cell cycle	-3.9	-6.7	-26.1
BE691552	Lactamase, beta 2 (Lactb2)	Metabolism	-2.3	-1.8	-4.2
AI848705	Lin-7 homolog A (Lin7a)	Protein complex assembly; exocytosis; neurotransmitter secretion; synaptic vesicle transport	-2.2	-2.8	-6.3
AV287586	Lin-7 homolog A (Lin7a)	Protein complex assembly; exocytosis; neurotransmitter secretion; synaptic vesicle transport	-2.6	-2.6	-6.8
NM_008280	Lipase, hepatic (Lipc)	Protein amino acid N-linked glycosylation; lipid metabolism	-2.8	-1.9	-5.4
BC013893	Mannan-binding lectin serine peptidase 2 (Masp2)	Proteolysis; innate immune response; complement activation, classical pathway; cell adhesion; antimicrobial humoral response (sensu	-2.1	-1.6	-3.2

		Vertebrata)			
BC020153	Nitrilase family, member 2 (Nit2)	Nitrogen compound metabolism	-4.4	-3.0	-13.2
NM_013864	N-myc downstream regulated gene 2 (Ndrg2)	Nervous system development; cell differentiation	-2.2	-1.7	-3.7
AK011172	Nudix (nucleoside diphosphate linked moiety X)-type motif 7 (Nudt7)	Coenzyme A catabolism; acetyl-CoA catabolism	-2.1	-2.8	-6.0
AI786408	Ornithine transcarbamylase (Otc)	Urea cycle; amino acid metabolism; arginine biosynthesis	-2.7	-1.5	-4.1
BC023496	Pantothenate kinase 1 (Pank1)	Coenzyme A biosynthesis	-4.4	-1.7	-7.7
NM_023523	Peroxisomal trans-2-enoyl-CoA reductase (Pecr)	Fatty acid biosynthesis; metabolism; lipid biosynthesis; fatty acid elongation	-2.1	-1.7	-3.5
BC019997	Phenazine biosynthesis-like protein domain containing (Pbld)	Interacts with unrip/mawd; Oxidoreductase activity	-3.6	-3.2	-11.4
AW106920	Phenylalanine hydroxylase (Pah)	L-phenylalanine catabolism; aromatic amino acid family metabolism	-8.7	-2.7	-23.7
BC013525	Pipecolic acid oxidase (Pipox)	Electron transport; peroxisome organization and biogenesis; tetrahydrofolate metabolism	-2.8	-2.1	-5.8
NM_008932	Prolactin receptor (Prlr)	Steroid biosynthesis; anti-apoptosis; transmembrane receptor protein tyrosine kinase activation (dimerization); embryo implantation; lactation; regulation of cell adhesion; regulation of epithelial cell differentiation; T cell activation; tyrosine phosphorylation of JAK2 protein	-3.3	-2.7	-8.7
NM_008932	Prolactin receptor (Prlr)	Steroid biosynthesis; anti-apoptosis; transmembrane receptor protein tyrosine kinase activation (dimerization); embryo implantation; lactation; regulation of cell adhesion; regulation of epithelial cell differentiation; T cell activation; tyrosine phosphorylation of JAK2 protein	-4.4	-2.7	-12.0

M22958	Prolactin receptor (Prlr)	Steroid biosynthesis; anti-apoptosis; transmembrane receptor protein tyrosine kinase activation (dimerization); embryo implantation lactation; regulation of cell adhesion; regulation of epithelial cell differentiation; T cell activation; tyrosine phosphorylation of JAK2 protein	-4.5	-3.0	-13.7
AK011039	Proline dehydrogenase (oxidase) 2 (Prodh2)	Glutamate biosynthesis; proline metabolism	-2.7	-2.0	-5.4
BC024104	Protein Z, vitamin K-dependent plasma glycoprotein (Proz)	Proteolysis; blood coagulation	-1.9	-1.6	-2.9
BB667651	Pyruvate kinase liver and red blood cell (Pklr)	Glycolysis; response to other organism	-2	-2.7	-5.3
NM_009040	Retinol dehydrogenase 16 (Rdh16)	Metabolism	-4.6	-2.9	-13.5
BI217574	Sarcosine dehydrogenase (Sardh)	Electron transport; glycine catabolism	-2.7	-2.6	-7.0
NM_029269	Secreted phosphoprotein 2 (Spp2)	Skeletal development; bone remodeling	-2.1	-1.9	-3.9
AF237702	Serine hydroxymethyl transferase 1 (soluble) (Shmt1)	Glycine metabolism; L-serine metabolism; one-carbon compound metabolism	-3.1	-2.7	-8.5
NM_009171	Serine hydroxymethyl transferase 1 (soluble) (Shmt1)	Glycine metabolism; L-serine metabolism; one-carbon compound metabolism	-3.5	-2.9	-10.0
BC021154	Solute carrier family 10 (sodium/bile acid cotransporter family), member 1 (Slc10a1)	Sodium ion transport; organic anion transport	-5	-2.2	-11.0
NM_011387	Solute carrier family 10 (sodium/bile acid cotransporter family), member 1 (Slc10a1)	Sodium ion transport; organic anion transport	-5.1	-2.3	-11.7
AV220703	Solute carrier family 16 (monocarboxylic acid transporters), member 12 (Slc16a12)	Proton-linked monocarboxylate transport	-2.7	-2.0	-5.4
NM_031197	Solute carrier family 2 (facilitated glucose transporter), member 2 (Slc2a2)	Carbohydrate metabolism; carbohydrate transport; glucose transport	-9.4	-2.1	-19.7
AA276202	Solute carrier family 23 (nucleobase transporters), member 1 (Slc23a1)	Sodium ion transport	-2.2	-1.5	-3.3
BC016571	Solute carrier family 25 (mitochondrial carrier, adenine nucleotide translocator), member 13 (Slc25a13)	Aspartate transport	-2.3	-2.0	-4.5
NM_025877	Solute carrier family 25 (mitochondrial carrier; phosphate carrier), member 23 (Slc25a23)	Transport	-2.2	-2.9	-6.3

BC022130	Solute carrier family 26 (sulfate transporter), member 1 (Slc26a1)	Chloride, sulfate, and oxalate transport	-2.7	-3.4	-9.3
BB275387	Solute carrier family 26 (sulfate transporter), member 1 (Slc26a1)	Chloride, sulfate, and oxalate transport	-4.7	-4.8	-22.7
AK003626	Solute carrier family 38, member 4 (Slc38a4)	Sodium ion transport; amino acid transport	-2.7	-1.7	-4.6
AB031813	Solute carrier organic anion transporter family, member 1a1 (Slco1a1)	Organic anion transport	-3.8	-1.7	-6.2
AB031813	Solute carrier organic anion transporter family, member 1a1 (Slco1a1)	Organic anion transport	-6	-2.3	-13.8
AK004939	Transmembrane serine protease 6 (Tmprss6)	Angiogenesis; proteolysis; intracellular signaling cascade; extracellular matrix organization and biogenesis; fibrinolysis	-2.6	-2.2	-5.6
NM_028094	UDP glucuronosyltransferase 2 family, polypeptide A3 (Ugt2a3)	Metabolism	-2.6	-2.1	-5.6
BC028826	UDP glucuronosyltransferase 2 family, polypeptide B34 (Ugt2b34)	Metabolism	-3	-2.4	-7.1
BC018406	cDNA	Unknown	-2	-1.6	-3.2
BC021614	cDNA	Metabolism	-2.5	-1.6	-4.0
AK009249	cDNA	Unknown	-2.3	-1.9	-4.3
BB822342	cDNA	Unknown	-2.4	-1.9	-4.6
AW111847	cDNA	Unknown	-2.2	-2.3	-5.0
BC025830	cDNA	Unknown	-2.5	-2.0	-5.2
AK016432	cDNA	Unknown	-2.8	-2.1	-6.1
BC018263	cDNA	Metabolism	-3.7	-1.7	-6.3
AI789751	cDNA	Unknown	-3	-3.3	-10.0
AK018631	cDNA	Unknown	-6	-1.7	-10.4
AI265721	cDNA	Unknown	-4.7	-2.2	-10.4
BB667865	cDNA	Unknown	-4.1	-2.6	-10.5
NM_026183	cDNA	Unknown	-4	-2.8	-11.3
AB056443	cDNA	Unknown	-4.6	-2.5	-11.4
NM_134256	cDNA	Transport	-5	-2.4	-12.1
AK012685	cDNA	Unknown	-6.6	-2.2	-14.5
AB056443	cDNA	Unknown	-5.9	-2.8	-16.5
Up-regulated in WT-infected Mice (But Not Mutant-infected Mice)					
AK014526	Lipin 1 (Lpin1)	Lipid metabolism; regulation of fat cell differentiation	-1.8	2.3	
Up-regulated in Mutant-infected Mice (But Not WT-infected Mice)					
BG969012	Activin A receptor, type II-like 1 (Acvrl1)	Angiogenesis; protein amino acid phosphorylation; transforming growth factor beta receptor signaling pathway	2.1	-	2.6
NM_007436	Aldehyde dehydrogenase family 3, subfamily A1 (Aldh3a1)	Aldehyde metabolism; electron transport; metabolism	2.8	-	3.3
BC027319	ATPase, Na+/K+ transporting, beta 1 polypeptide (Atp1b1)	Potassium ion transport; sodium ion transport	1.7	-	2.3

NM_007689	Chondroadherin (Chad)	Regulation of cell growth; metabolism	2	-	2.2
AW060797	Coiled-coil domain containing 85A (Ccdc85a)	Unknown	2.2	-	3.2
BB417442	C-type lectin domain family 1, member a (Clec1a)	Cell adhesion	2	-	2.8
BE685667	Cyclin D3 (Ccnd3)	Regulation of progression through cell cycle; signal transduction; T cell proliferation; cell division	2	-	2.1
AA410148	Dedicator of cytokinesis 9 (Dock9)	Guanyl nucleotide exchange factor activity	1.9	-	2.7
BB626288	Gene model 1752 (Gm1752)	Unknown	1.9	-	2.8
NM_016677	Hippocalcin-like 1 (Hpcal1)	May be involved in the calcium-dependent regulation of rhodopsin phosphorylation and may be of relevance for neuronal signalling in the central nervous system	1.7	-	2.3
BG066605	Laminin, gamma 1 (Lamc1)	Protein complex assembly; cell adhesion; endoderm development; positive regulation of epithelial cell proliferation; positive regulation of epithelial cell proliferation	1.8	-	2.1
BG066982	Natriuretic peptide receptor 3 (Npr3)	Skeletal development	5.1	-	6.0
NM_013626	Peptidylglycine alpha-amidating monooxygenase (Pam)	Peptide metabolism	2.1	-	2.2
BQ177191	Prickle-like 2 (Drosophila) (Prickle2)	Unknown	2.6	-	3.5
AF169388	Procollagen, type IV, alpha 4 (Col4a4)	Phosphate transport; cell adhesion	2	-	3.0
BB305930	Protocadherin 17 (Pcdh17)	Cell adhesion	2.2	-	3.0
AF112185	Sodium channel, nonvoltage-gated, type I, alpha (Scnn1a)	Sodium ion transport; response to stimulus	1.8	-	2.6
BC025840	Titin (Ttn)	Carbohydrate metabolism; RNA-dependent DNA replication; protein amino acid phosphorylation; sarcomere alignment; response to oxidative stress; muscle development	1.8	-	2.2
BM122301	Transforming growth factor, beta receptor III (Tgfbr3)	Binds to TGF-beta; could be involved in capturing and retaining TGF-beta for presentation to the signaling receptors	1.8	-	2.6
BG064061	Transmembrane protein 164 (Tmem164)	Unknown	1.8	-	2.3

NM_021883	Tropomodulin 1 (Tmod1)	Cytoskeleton organization and biogenesis; blocks the elongation and depolymerization of the actin filaments at the pointed end	2.3	-	3.3
AK019588	Ubiquitin specific peptidase 54 (Usp54)	Proteolysis; ubiquitin cycle	1.7	-	2.2
BB447914	Zinc finger protein 503 (Zfp503)	Unknown	2	-	2.4
BC003967	cDNA	Unknown	2.2	-	3.3
BB535847	cDNA	Unknown	2.3	-	2.5
BB520487	cDNA	Unknown	1.7	-	2.4
AK006115	cDNA	Unknown	1.6	-	2.3
Down-regulated in Mutant-infected Mice (But Not WT-infected Mice)					
NM_134130	Abhydrolase domain containing 3 (Abhd3)	Unknown	-2.1	-	-2.9
AK017155	Agmatine ureohydrolase (agmatinase) (Agmat)	Arginine catabolism; polyamine biosynthesis	-2.5	-	-3.5
BB155250	Alkylglycerone phosphate synthase (Agps)	Electron transport; lipid metabolism	-2.1	-	-2.9
NM_007493	Asialoglycoprotein receptor 2 (Asgr2)	Endocytosis	-2.2	-	-3.0
NM_016668	Betaine-homocysteine methyltransferase (Bhmt)	Protein amino acid methylation; methionine biosynthesis; regulation of homocysteine metabolism	-2.9	-	-3.5
NM_022884	Betaine-homocysteine methyltransferase 2 (Bhmt2)	Methionine biosynthesis	-1.9	-	-2.4
NM_133960	Carboxylesterase 6 (Ces6)	Unknown	-2.1	-	-2.8
NM_007744	Catechol-O-methyltransferase (Comt)	Catecholamine metabolism; neurotransmitter catabolism; dopamine catabolism	-2.6	-	-2.8
NM_007744	Catechol-O-methyltransferase (Comt)	Catecholamine metabolism; neurotransmitter catabolism; dopamine catabolism	-2.7	-	-3.4
NM_015780	Complement factor H-related 1 (Cfhr1)	Immune response	-2.9	-	-3.4
NM_010003	Cytochrome P450, family 2, subfamily c, polypeptide 39 (Cyp2c39)	Electron transport	-3.1	-	-4.7
BF783609	Cytochrome P450, family 2, subfamily j, polypeptide 5 (Cyp2j5)	Electron transport	-2.9	-	-3.7
AB039380	Cytochrome P450, family 3, subfamily a, polypeptide 44 (Cyp3a44)	Electron transport	-5.8	-	-6.8
BC028831	Dihydropyrimidine dehydrogenase (Dpyd)	Electron transport; 'de novo' pyrimidine base biosynthesis	-2.5	-	-3.0
BC028831	Dihydropyrimidine dehydrogenase (Dpyd)	Electron transport; 'de novo' pyrimidine base biosynthesis	-3.3	-	-4.7
AK004755	Dimethylglycine dehydrogenase precursor (Dmgdh)	Electron transport; glycine catabolism; protein targeting	-1.9	-	-2.2

NM_007954	Esterase 1 (Es1)	Metabolism	-2.2	-	-2.9
NM_010232	Flavin containing monooxygenase 5 (Fmo5)	Electron transport	-2.7	-	-2.7
NM_010232	Flavin containing monooxygenase 5 (Fmo5)	Electron transport	-3	-	-3.1
AI391218	Glutamate-ammonia ligase (glutamine synthetase) (Glul)	Glutamine biosynthesis; nitrogen compound metabolism	-2.1	-	-2.3
AK005016	Heat-responsive protein 12 (Hrsp12)	regulation of translational termination	-2.1	-	-2.9
NM_010517	Insulin-like growth factor binding protein 4 (Igfbp4)	Skeletal development; regulation of cell growth; DNA metabolism; signal transduction; cell proliferation	-2.1	-	-2.5
NM_133809	Kynurenine 3-monooxygenase (kynurenine 3-hydroxylase) (Kmo)	Electron transport; aromatic compound metabolism	-2	-	-2.6
AK018630	Nuclear receptor subfamily 1, group I, member 2 (Nrli2)	Transcription regulation; xenobiotic metabolism; signal transduction; steroid metabolism	-2.3	-	-3.2
NM_008769	Ornithine transcarbamylase (Otc)	Urea cycle; amino acid metabolism; arginine biosynthesis	-2.9	-	-3.9
NM_020573	Oxysterol binding protein-like 1A (Osbpl1a)	Lipid transport; steroid metabolism; cholesterol metabolism; vesicle-mediated transport	-2.1	-	-2.5
AI265463	Phosphoenolpyruvate carboxykinase 1, cytosolic (Pck1)	Gluconeogenesis; lipid metabolism; glycerol biosynthesis from pyruvate	-3.5	-	-3.3
NM_011082	Polymeric immunoglobulin receptor (Pigr)	Protein secretion	-2.1	-	-2.8
BG071940	Protein phosphatase 1, regulatory (inhibitor) subunit 3B (Ppr1r3b)	Glycogen metabolism	-3.5	-	-5.0
NM_011261	Reelin (Reln)	Cellular morphogenesis during differentiation; neuron migration; cell adhesion; development; axon guidance; central nervous system development; brain development; locomotory behavior; glial cell differentiation; peptidyl-tyrosine phosphorylation; spinal cord patterning; positive regulation of protein kinase activity; response to pain; positive regulation of small GTPase mediated signal transduction	-1.8	-	-2.5

NM_009060	Regucalcin (Rgn)	May play a role in the regulation of enzymatic activity in the liver; decrease of rgn leads to the dysregulation of calcium signaling in the aged liver	-3.1	-	-3.9
BB035017	Retinoic acid receptor responder (tazarotene induced) 1 (Rarres1)	Negative regulation of cell proliferation	-3.1	-	-3.2
BC013442	Solute carrier family 27 (fatty acid transporter), member 2 (Slc27a2)	Very-long-chain fatty acid metabolism; lipid metabolism; fatty acid metabolism	-2.6	-	-3.5
NM_008063	Solute carrier family 37 (glycerol-6-phosphate transporter), member 4 (Slc37a4)	Carbohydrate metabolism and transport	-2.6	-	-3.4
AB037192	Solute carrier organic anion transporter family, member 1b2 (Slco1b2)	Ion transport	-2.6	-	-2.4
NM_020495	Solute carrier organic anion transporter family, member 1b2 (Slco1b2)	Ion transport	-2.5	-	-2.7
D87867	UDP glucuronosyltransferase 1 family, polypeptide A2 (Ugt1a2)	Glucuronate metabolism; xenobiotic metabolism; protein folding	-1.8	-	-2.2
NM_053215	UDP glucuronosyltransferase 2 family, polypeptide B37 (Ugt2b37)	Metabolism	-5.1	-	-6.3
BC022133	Urocanase domain containing 1 (Uroc1)	Histidine metabolism	-2.2	-	-3.0
BC025105	V-set and immunoglobulin domain containing 4 (Vsig4)	Negative regulation of murine and human T cell proliferation and IL-2 production	-2.3	-	-2.8
AV030118	cDNA	Unknown	-2.1	-	-2.1
AW488243	cDNA	Unknown	-2.4	-	-2.3
NM_009286	cDNA	Unknown	-2.4	-	-2.7
AV222559	cDNA	Unknown	-2	-	-2.9
AJ132857	cDNA	Transport	-3.1	-	-3.9

Fold-changes (FC) shown were statistically significant (i.e., Benjamini and Hochberg corrected p value <0.05 and absolute consistency across all replicate experiments). A negative sign before values indicates down-regulation. A dash "-" indicates no statistical difference was observed. C = control (uninfected) mice; WT=wild-type *Y. pestis* CO92 infected mice; Mut=Δ*lpp* mutant of *Y. pestis* CO92 infected mice.

Supplementary Table VII. Genes statistically differentially expressed in the lungs of mice infected for 48 hr with a Δ*lpp* mutant of *Y. pestis* CO92, compared to WT bacteria

GenBank ID	Gene Name	Function	WT vs Mut	C vs WT	C vs Mut
				FC	
Up-regulated in Both WT- and Mutant-infected Mice					
BB443585	A disintegrin-like and metalloprotease (reprolysin type) with thrombospondin type 1 motif, 4 (Adamts4)	Skeletal development; integrin-mediated signaling; proteolysis; metallopeptidase activity	4.02	2.2	8.9
BG064671	A disintegrin-like and metalloprotease (reprolysin type) with thrombospondin type 1 motif, 4 (Adamts4)	Skeletal development; integrin-mediated signaling; proteolysis; metallopeptidase activity	6.63	2.6	17.5
BC026209	Arachidonate 5-lipoxygenase activating protein (Alox5ap)	Leukotriene biosynthesis; inflammatory response	1.87	1.6	3
AV002218	Arginase type II (Arg2)	Urea cycle; arginine metabolism; nitric oxide biosynthesis; striated muscle contraction	4.53	2.6	11.9
AK011545	Brain abundant, membrane attached signal protein 1 (Basp1)	Transcription regulation	3.14	1.5	4.8
NM_007609	Caspase 4, apoptosis-related cysteine peptidase (Casp4)	Proteolysis; regulation of apoptosis	2.18	2	4.3
AV006463	cDNA	Unknown	2.73	2.2	6
BB239429	cDNA	Unknown	3.26	5.8	18.9
AF065933	Chemokine (C-C motif) ligand 2 (Ccl2)	Chemotaxis; inflammatory response; immune response; signal transduction	5.05	3.4	17.1
AF099052	Chemokine (C-C motif) ligand 20 (Ccl20)	Chemotaxis; inflammatory response; immune response; signal transduction; cell-cell signaling; antimicrobial humoral response	4.69	1.6	7.5
NM_011337	Chemokine (C-C motif) ligand 3 (Ccl3)	Chemotaxis; inflammatory response; immune response; signal transduction	10.02	4.1	40.8
AF128218	Chemokine (C-C motif) ligand 4 (Ccl4)	Chemotaxis; inflammatory response; immune response; signal transduction	7.92	2.1	16.9

AJ318863	Chemokine (C-C motif) receptor-like 2 (Ccrl2)	G-protein coupled receptor protein signaling pathway	3.75	2.6	9.6
BB554288	Chemokine (C-X-C motif) ligand 1 (Cxcl1)	Regulation of progression through cell cycle; inflammatory response; immune response	3.12	3.6	11.2
BB554288	Chemokine (C-X-C motif) ligand 1 (Cxcl1)	Regulation of progression through cell cycle; inflammatory response; immune response	3.83	4.9	18.7
NM_019494	Chemokine (C-X-C motif) ligand 11 (Cxcl11)	Chemotaxis; inflammatory response; immune response; signal transduction; cell-cell signaling	3.59	10.9	39.2
NM_009140	Chemokine (C-X-C motif) ligand 2 (Cxcl2)	Chemotaxis; inflammatory response; immune response; signal transduction	9.29	18.2	169.1
NM_009141	Chemokine (C-X-C motif) ligand 6 (Cxcl6)	Chemotaxis; inflammatory response; immune response; signal transduction	7.41	9.3	69
NM_009971	Colony stimulating factor 3 (granulocyte) (Csf3)	Immune response; cellular defense response; cell-cell signaling; development; positive regulation of cell proliferation; cytokine and chemokine mediated signaling pathway; granulocyte differentiation	5.7	2.1	11.9
NM_010819	C-type lectin domain family 4, member d (Clec4d)	Immune response	3.48	3.6	12.6
NM_019948	C-type lectin domain family 4, member e (Celc4e)	Immune response; cell adhesion; antimicrobial humoral response	6.67	2.3	15.5
NM_019948	C-type lectin domain family 4, member e (Celc4e)	Immune response; cell adhesion; antimicrobial humoral response	4.21	6.8	28.4

NM_007950	Epiregulin (Ereg)	Regulation of progression through cell cycle; angiogenesis; positive regulation of cytokine production; epidermal growth factor receptor signaling pathway; development; cell proliferation; transcription regulation; organ morphogenesis; cytokine and chemokine mediated signaling pathway; cell differentiation; luteinizing hormone signaling pathway; keratinocyte proliferation; positive regulation of innate immune response; positive regulation of IL-6 biosynthesis; positive regulation of fibroblast proliferation /// primary follicle stage oogenesis (sensu Mammalia) /// positive regulation of smooth muscle cell proliferation /// negative regulation of epithelial cell proliferation; negative regulation of epithelial cell proliferation; negative regulation of smooth muscle cell differentiation	5.74	1.6	9.3
BG067531	EST	Unknown	2.56	2.5	6.4
BG961961	EST	Unknown	6.79	5.5	37.4
AV370830	Free fatty acid receptor 2 (Ffar2(	G-protein coupled receptor protein signaling pathway	1.95	1.5	3
BB829808	Gene model 1960 (Gm1960)	Immune response	9.15	15.9	145.1
U05264	Glycoprotein 49 A (Gp49a); also called leukocyte immunoglobulin-like receptor, subfamily B, member 4 (Lilrb4)	Immune response	2.95	2.4	7.2
BC011246	Hemopexin (Hpxn)	Iron ion homeostasis; heme transport	2.53	2.3	5.8

BM246462	Hypothetical Immunoglobulin-like containing protein	Unknown	2.28	1.9	4.4
NM_030691	Immunoglobulin superfamily, member 6 (Igsf6)	Cell adhesion and immune functions (by inference)	2.83	2.8	7.8
L38281	Immunoresponsive gene 1 (Irg1)	Immune response	7.09	10.9	77.4
NM_019777	Inhibitor of kappaB kinase epsilon (Ikbke)	Protein amino acid phosphorylation; immune response; regulation of I-kappaB kinase/NF-kappaB cascade	2.61	1.8	4.7
NM_011940	Interferon activated gene 202B (Ifi202b)	Immune response	2.92	2.9	8.5
K00083	Interferon gamma (Ifng)	Regulation of cell growth; neutrophil apoptosis; inflammatory cell apoptosis; cell motility; immune response; response to virus; antigen processing and presentation; neutrophil chemotaxis; unfolded protein response; negative regulation of myelination; defense response to bacterium ; positive regulation of chemokine biosynthesis	6.68	4.3	28.4
BC003727	Interleukin 1 alpha (IL-1a)	Rregulation of progression through cell cycle; fever; regulation of apoptosis; chemotaxis; inflammatory response; immune response; cell-cell signaling' regulation of cell proliferation	6.75	1.6	10.5
AY071843	Interleukin 1 family, member 9 (IL1f9)	Immune response; cell-cell signaling	4.45	2.1	9.4
M57525	Interleukin 1 receptor antagonist (Ilrn1)	Lipid metabolism; inflammatory response; immune response; cell surface receptor linked signal transduction;insulin secretion	5.13	3.8	19.7
NM_010555	Interleukin 1 receptor, type II (Il1r2)	Immune response; cell surface receptor linked signal transduction	3.96	5.2	20.7

NM_010552	Interleukin 17A (Il17a)	Protein amino acid glycosylation; apoptosis; inflammatory response; cell-cell signaling; cell death	2.73	1.7	4.5
NM_031168	Interleukin 6 (IL-6)	Neutrophil apoptosis; acute-phase response; humoral immune response; cell surface receptor linked signal transduction; cell-cell signaling; regulation of cell proliferation; regulation of chemokine biosynthesis; positive regulation of T-helper 2 cell differentiation	5.01	34.3	172
AV228493	Interleukin-1 receptor-associated kinase 3 (Irak3)	Protein amino acid phosphorylation; cytokine and chemokine mediated signaling pathway; regulation of apoptosis; regulation of protein complex disassembly; sognal transduction	3.36	1.6	5.3
BC006948	Lymphocyte cytosolic protein 2 (Lcp2)	Immune response; transmembrane receptor protein tyrosine kinase signaling pathway; intracellular signaling cascade; mast cell activation; cytokine secretion	2.62	1.6	4.2
AV110584	MARCKS-like 1	May be involved in coupling the protein kinase c and calmodulin signal transduction systems	3.64	2.8	10.3
NM_010809	Matrix metallopeptidase 3 (Mmp3)	Peptidoglycan metabolism; proteolysis; collagen catabolism	4.51	2	8.9
NM_008611	Matrix metallopeptidase 8 (Mmp8)	Peptidoglycan metabolism; proteolysis; collagen catabolism	2.75	5.6	15.5
NM_013599	Matrix metallopeptidase 9 (Mmp9)	Peptidoglycan metabolism; skeletal development; proteolysis; extracellular matrix organization and biogenesis; macrophage differentiation; collagen catabolism; positive regulation of apoptosis	2.62	1.9	5

AV228517	MAX dimerization protein 1 (Mxd1)	Transcription regulation; development; cell proliferation	3.54	4.5	16
NM_019453	Mediterranean fever (Mefv)	Inflammatory response	2.71	2.6	6.9
AI462015	Nuclear factor of kappa light chain gene enhancer in B-cells inhibitor, alpha (Nfkbia)	Apoptosis; cytoplasmic sequestering of NF-kappaB; regulation of cell proliferation; regulation of NF-kappaB import into nucleus; negative regulation of DNA binding; negative regulation of myeloid cell differentiation; negative regulation of Notch signaling pathway	1.8	2.1	3.8
AK011965	Nuclear factor of kappa light polypeptide gene enhancer in B-cells inhibitor, epsilon (Nfkbie)	Transcription regulation; cytoplasmic sequestering of transcription factor	2.46	1.8	4.4
AB026551	Nuclear factor of kappa light polypeptide gene enhancer in B-cells inhibitor, zeta (Nfkbiz)	Transcription regulation; inflammatory response	4.54	2.1	9.6
AB026551	Nuclear factor of kappa light polypeptide gene enhancer in B-cells inhibitor, zeta (Nfkbiz)	Transcription regulation; inflammatory response	3.18	2.6	8.3
AY061760	Nuclear factor, interleukin 3, regulated (Nfil3)	Transcription regulation	3.07	3	9.3
AV290148	Olfactomedin 4 (Olfm4)	Unknown	3.75	7.3	27.3
NM_008987	Pentraxin related gene (Ptx3)	Response to yeast; inflammatory response; opsonization; positive regulation of nitric oxide biosynthesis; positive regulation of phagocytosis	5.39	3.3	17.8
AV230647	Phosphoinositide-3-kinase, regulatory subunit 5, p101 (Pik3r5)	Signal transduction	1.96	1.5	3
AK005158	Phospholipase A2, group VII (platelet-activating factor acetylhydrolase, plasma) (Pla2g7)	Inflammatory response; lipid catabolism	2.64	2	5.2
AF181829	Pleckstrin (Plek)	Intracellular signaling cascade	3.6	1.7	6.3

M94967	Prostaglandin-endoperoxide synthase 2 (Ptgs2)	Prostaglandin biosynthesis; fatty acid biosynthesis; cell motility; blood pressure regulation; lipid biosynthesis; cyclooxygenase pathway; keratinocyte differentiation; anagen; regulation of inflammatory response	5.64	1.7	9.8
BB223018	Schlafen 1 (Slfn1)	G1/S transition of mitotic cell cycle; cell cycle arrest; negative regulation of cell proliferation	3.19	1.6	5
M72332	Selectin, platelet (Selp)	Inflammatory response; cell adhesion	2.64	2.1	5.5
NM_008871	Serine (or cysteine) peptidase inhibitor, clade E, member 1 (Serpine1)	Blood coagulation; fibrinolysis; regulation of angiogenesis	3.67	4	14.8
NM_011314	Serum amyloid A 1 (Saa1)	Acute-phase response	6.91	5.2	36
NM_009117	Serum amyloid A 1 (Saa1)	Acute-phase response	9.33	7.7	71.9
NM_011314	Serum amyloid A 2 (Saa2)	Acute-phase response	3.68	2.1	7.7
NM_023044	Solute carrier family 15, member 3 (Slc15a3)	Oligopeptide transport	2.65	2.2	5.8
BM209618	Solute carrier family 2 (facilitated glucose transporter), member 1 (Slc2a1)	Carbohydrate transport; glucose transport	3.23	2	6.6
BB399837	Solute carrier family 39 (zinc transporter), member 14 (Slc39a14)	Metal ion transport	2.42	2.4	5.7
AK010399	Sorting nexin 10 (Snx10)	Cell communication; intracellular signaling cascade; protein transport	2.14	1.8	3.8
BB667930	Stefin A2 like 1 (Stfa2l1)	Unknown	3.92	3.4	13.3
NM_013671	Superoxide dismutase 2, mitochondrial (Sod2)	Response to superoxide; age-dependent response to reactive oxygen species; transcription regulation; superoxide metabolism; response to oxidative stress	2.91	1.7	4.9

NM_013671	Superoxide dismutase 2, mitochondrial (Sod2)	Response to superoxide; age-dependent response to reactive oxygen species; transcription regulation; superoxide metabolism; response to oxidative stress	2.58	1.9	4.8
BB277065	Traf2 binding protein (T2bp)	I-kappaB kinase/NF-kappaB cascade	2.5	2.7	6.9
BB784999	Triggering receptor expressed on myeloid cells 1 (Trem1)	Humoral immune response; intracellular signaling cascade	3.67	2	7.2
NM_021406	Triggering receptor expressed on myeloid cells 1 (Trem1)	Humoral immune response; intracellular signaling cascade	5.12	2.5	12.8
BM241351	Tumor necrosis factor, alpha-induced protein 3 (Tnfaip3)	Ubiquitin cycle; regulation of apoptosis; negative regulation of I-kappaB kinase/NF-kappaB cascade	3.5	2.7	9.5
Up-regulated in Mutant-infected Mice (But Not WT-infected Mice)					
BC011338	Baculoviral IAP repeat-containing 3 (Birc3)	Regualtion of apoptosis; cell surface receptor linked signal transduction	1.83	-	2.2
L16462	B-cell leukemia/lymphoma 2 related protein A1a (Bcl2a1a)	Regulation of apoptosis	2.34	-	3.5
NM_007651	CD53 antigen (Cd53)	Signal transduction; antimicrobial humoral response	1.99	-	2.1
AV231983	cDNA	Unknown	3.1	-	4.2
AK016659	cDNA	Lipid metabolism; sphingolipid metabolism; sphingomyelin biosynthesis	2.65	-	2.7
BB531414	cDNA	Unknown	2.12	-	3.1
AF252873	Chemokine (C-X-C motif) ligand 14 (Cxcl14)	Chemotaxis; inflammatory response; immune response; signal transduction; cell-cell signaling	2.71	-	2.7
BC024886	Coagulation factor III (F3)	Immune response; blood coagulation	2.18	-	2.4
NM_007972	Coagulation factor X (F10)	Proteolysis; blood coagulation	2.41	-	3
BM238701	Dual specificity phosphatase 16 (Dusp16)	Inactivation of MAPK activity; protein amino acid dephosphorylation	2.63	-	2.7

NM_007913	Early growth response 1 (Egr1)	Transcription regulation; T cell differentiation	3.03	-	3
BB284358	EGL nine homolog 3 (Egln3)	Apoptosis; protein metabolism	3.81	-	3.6
NM_010119	EH-domain containing 1 (Ehd1)	Eendocytosis; endosome transport	2.18	-	2.5
BM234652	ERO1-like (S. cerevisiae)	electron transport /// protein folding /// protein folding /// transport /// unfolded protein response /// protein biosynthesis /// chaperone cofactor-dependent protein folding	3.59	-	3.7
NM_013521	Formyl peptide receptor 1 (Fpr1)	Activation of MAPK activity; cell motility; chemotaxi; inflammatory response; G-protein signaling, coupled to cAMP; nitric oxide mediated signal transduction	2.47	-	3.6
AV370830	Free fatty acid receptor 2 (Ffar2(	G-protein coupled receptor protein signaling pathway	2.47	-	3.6
AF276917	Glutaredoxin (Glrx)	Electron transport; cell redox homeostasis	2.17	-	2.4
BF683028	Glycerol kinase (Gyk)	Carbohydrate metabolism; glycerol metabolism; glycerol-3-phosphate metabolism	2.37	-	3
NM_013820	Hexokinase 2 (Hk2)	Regulation of progression through cell cycle; glucose metabolism; glycolysis	2.2	-	2.4
BG072171	Histidine decarboxylase (Hdc)	Amino acid and derivative metabolism; histidine and carboxylic acid metabolism; catecholamine biosynthesis	3.12	-	2.9
BB102620	Hypothetical protein	Unknown	4.02	-	5.1
X95580	Hypoxia inducible factor 1, alpha subunit (Hif1a)	Two-component signal transduction system (phosphorelay); angiogenesis; response to hypoxia; neural crest cell migration; heart looping; transcription regulation; cell differentiation; homeostasis	2.23	-	2.6

NM_008332	Interferon-induced protein with tetratricopeptide repeats 2 (Ifit2)	Immune response	3.57	-	4.9
AJ249492	Interleukin 22 (IL22); also called interleukin 10-related T cell-derived inducible factor beta (Iltifb)	Oxygen and reactive oxygen species metabolism; immune response; regulation of tyrosine phosphorylation of Stat3 protein	2.89	-	3.7
BM247104	Kelch-like 6 (Klhl6)	Unknown	1.9	-	2.7
AA270173	Lamin B1 (Lmnb1)	Component of the nuclear lamina; thought to provide a framework for the nuclear envelope; may interact with chromatin; thought to be involved in nuclear stability, chromatin structure and gene expression	3.55	-	4.9
AF065917	Leukemia inhibitory factor (Lif)	Immune response; cell surface receptor linked signal transduction; cell-cell signaling; development; positive regulation of cell proliferation; tyrosine phosphorylation of Stat3 protein; muscle morphogenesis; neuron development	3.36	-	4.7
BB491008	MARCKS-like 1	May be involved in coupling the protein kinase c and calmodulin signal transduction systems	2.96	-	3.6
AK014467	Mucolipin 2 (Mcoln2)	Cation transport	2.52	-	3.6
AI844633	Neutrophil cytosolic factor 1 (Ncf1)	Electron transport; leukotriene metabolism; NADP catabolism; inflammatory response; cellular defense response; cell communication; cell proliferation; response to bacterium	2.68	-	3.3
BB775785	Paired immunoglobin-like type 2 receptor alpha (Pilra)	Signal transduction	2.29	-	2.7
BG793493	Phosphodiesterase 4B, cAMP specific (Pde4b)	Smooth muscle contraction; signal transduction	2.56	-	2.3

BM246564	Phosphodiesterase 4B, cAMP specific (Pde4b)	Smooth muscle contraction; signal transduction	2.15	-	2.5
AI561611	PML-RAR alpha-regulated adaptor molecule 1 (Pram1)	Integrin-mediated signaling pathway; regulation of neutrophil degranulation	1.79	-	2.2
BB730139	Prostaglandin E synthase (Ptges)	Prostaglandin metabolism; signal transduction; antimicrobial humoral response	2.55	-	3
AK008416	RAP2C, member of RAS oncogene family (Rap2c)	Small GTPase mediated signal transduction; protein transport	1.96	-	2.3
BB667823	Ring finger protein 125 (Rnf125)	Ubiquitin cycle; immune response	1.99	-	2.1
T12280	Ring finger protein 149 (Rnf149)	Proteolysis; ubiquitin cycle	2.26	-	2.9
NM_023380	SAM domain, SH3 domain and nuclear localization signals, 1 (Samsn1)	Putative adaptor and scaffold protein	5.27	-	7.1
AV271736	Spermatogenesis associated 13 (Spata13)	Regulation of Rho protein signal transduction	2.2	-	3.1
NM_025288	Stefin A3 (Stfa3); also called cystatin A (Csta)	Cysteine protease inhibition; forms tight complexes with papain and the cathepsins B, H, and L; encodes one of the precursor proteins of cornified cell envelope in keratinocytes and plays a role in epidermal development and maintenance; Stefins have been proposed as prognostic and diagnostic tools for cancer	2.42	-	3.3
BC010465	Testis derived transcript (Tes)	May act as a tumor suppressor	2.25	-	2.1

Fold-changes (FC) shown were statistically significant (i.e., Benjamini and Hochberg corrected p value <0.05 and absolute consistency across all replicate experiments). A negative sign before values indicates down-regulation. A dash "-" indicates no statistical difference was observed. C = control (uninfected) mice; WT=wild-type *Y. pestis* CO92 infected mice; Mut=Δ*lpp* mutant of *Y. pestis* CO92 infected mice.
